# Ultra-High Resolution 3D Imaging of Whole Cells

**DOI:** 10.1016/j.cell.2016.06.016

**Published:** 2016-08-11

**Authors:** Fang Huang, George Sirinakis, Edward S. Allgeyer, Lena K. Schroeder, Whitney C. Duim, Emil B. Kromann, Thomy Phan, Felix E. Rivera-Molina, Jordan R. Myers, Irnov Irnov, Mark Lessard, Yongdeng Zhang, Mary Ann Handel, Christine Jacobs-Wagner, C. Patrick Lusk, James E. Rothman, Derek Toomre, Martin J. Booth, Joerg Bewersdorf

**Affiliations:** 1 Department of Cell Biology, School of Medicine, Yale University, New Haven, CT 06520, USA; 2 Weldon School of Biomedical Engineering, Purdue University, West Lafayette, IN 47907, USA; 3 The Gurdon Institute, University of Cambridge, Cambridge CB2 1QN, UK; 4 Department of Chemistry, Harvey Mudd College, Claremont, CA 91711, USA; 5 Department of Biomedical Engineering, Yale University, CT 06520, USA; 6 Microbial Sciences Institute, Yale University, West Haven, CT 06516, USA; 7 Department of Molecular, Cellular and Developmental Biology, Yale University, New Haven, CT 06520, USA; 8 The Jackson Laboratory, Bar Harbor, ME 04609, USA; 9 Howard Hughes Medical Institute, Yale University, New Haven, CT 06520, USA; 10 Department of Microbial Pathogenesis, Yale School of Medicine, New Haven, CT 06520, USA; 11 Nanobiology Institute, Yale University, West Haven, CT 06516, USA; 12 Department of Engineering Science, University of Oxford, Oxford OX1 3PJ, UK; 13 Centre for Neural Circuits and Behaviour, University of Oxford, Oxford OX1 3SR, UK

## Abstract

Fluorescence nanoscopy, or super-resolution microscopy, has become an important tool in cell biological research. However, because of its usually inferior resolution in the depth direction (50–80 nm) and rapidly deteriorating resolution in thick samples, its practical biological application has been effectively limited to two dimensions and thin samples. Here, we present the development of whole-cell 4Pi single-molecule switching nanoscopy (W-4PiSMSN), an optical nanoscope that allows imaging of three-dimensional (3D) structures at 10- to 20-nm resolution throughout entire mammalian cells. We demonstrate the wide applicability of W-4PiSMSN across diverse research fields by imaging complex molecular architectures ranging from bacteriophages to nuclear pores, cilia, and synaptonemal complexes in large 3D cellular volumes.

## Highlights

• Whole-cell 4Pi single-molecule switching nanoscopy allows 10- to 20-nm 3D resolution
• Refined hardware and new data analysis allow imaging of cells as thick as ∼10 μm
• Using structure-averaging, the 3D shape of a bacteriophage can be resolved
• Wide applicability across diverse research fields is demonstrated

## Graphical Abstract

**Figure:**
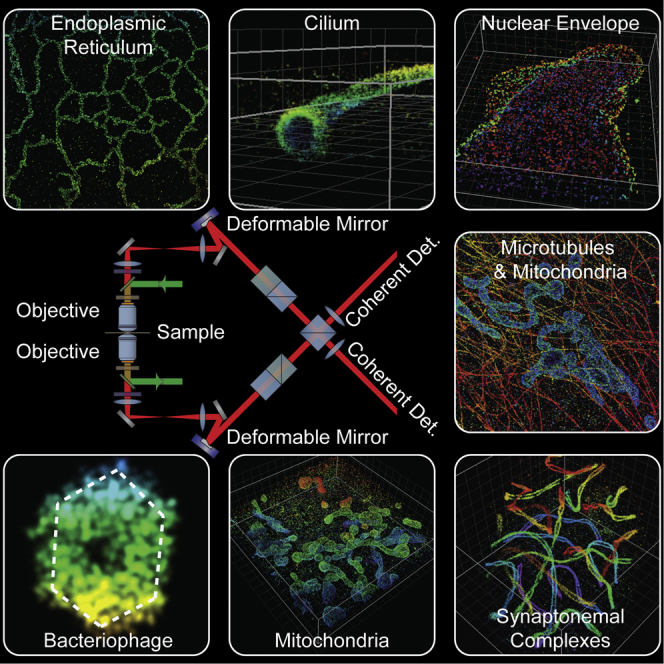

## Teaser

A new super-resolution microscope opens a window deep into cells to image organelles and subcellular structures spanning large volumes.

## Introduction

Major advances in cell biology are tightly linked to innovations in microscopy. The development of fluorescence microscopy, for example, enabled sub-cellular localization of specifically labeled proteins of interest (Lichtman and Conchello, 2005). However, the wave nature of light restricts the resolution of conventional light microscopy to ∼200 nm, making details of subcellular structures and protein assemblies unresolvable (Hell, 2007). The advent of super-resolution fluorescence microscopy, or nanoscopy, techniques such as stimulated emission depletion (STED) (Hell and Wichmann, 1994) and single-molecule switching nanoscopy (SMSN) (Betzig et al., 2006, Hess et al., 2006, Rust et al., 2006) has extended the application range of fluorescence microscopy beyond the diffraction limit, achieving up to 10-fold improvement in resolution (Gould et al., 2012a). These methods are now maturing and offering the opportunity to observe biological phenomena never before seen (Chojnacki et al., 2012, Kanchanawong et al., 2010, Liu et al., 2011, Xu et al., 2013). Nanoscopy techniques share a common principle: they spatially separate unresolvable fluorescent molecules by independently switching their emission “on” and “off” (Hell, 2007). In particular, SMSN methods such as photoactivated localization microscopy (PALM), fluorescence photoactivation localization microscopy (FPALM), and stochastic optical reconstruction microscopy (STORM) use a stochastic approach where only a small subset of fluorescent molecules is switched on at any particular moment in time while the majority remains in a non-fluorescent “dark” or “off” state (Gould et al., 2012a). Super-resolved images are reconstructed from the positions of thousands to millions of single molecules that have been recorded in thousands of camera frames.

This imaging strategy was initially applied to single-objective microscopes in two dimensions (2D) (Betzig et al., 2006, Hess et al., 2006, Rust et al., 2006) and later extended to three dimensions (3D) (Huang et al., 2008, Juette et al., 2008, Pavani et al., 2009). While these instruments achieve 20- to 40-nm resolution in the focal plane (lateral, x-y), the resolution in the depth direction (axial, z) is typically limited to only 50–80 nm. The resolution can, however, be further improved by using a dual-objective “4Pi” detection geometry (Bewersdorf et al., 2006).

Using two objectives doubles the detection efficiency (Xu et al., 2012) and thus improves the localization precision ∼1.4-fold in all three dimensions. Additionally, employing two objectives in a 4Pi geometry allows the creation of a single-molecule emission interference pattern at the detector leading to an ∼7-fold improvement in axial localization precision over single-objective approaches as demonstrated using interferometric PALM (iPALM) (Shtengel et al., 2009) and 4Pi single marker switching nanoscopy (4Pi-SMSN) (Aquino et al., 2011). This improved resolution enabled, for example, the generation of anatomical maps of focal adhesions at ∼10-nm axial resolution (Case et al., 2015, Kanchanawong et al., 2010). However, this method was initially restricted to samples of ∼250 nm in thickness (Shtengel et al., 2009) and more recently to 700–1,000 nm (Aquino et al., 2011, Brown et al., 2011). As the typical thickness of a mammalian cell is 5–10 μm, this has limited optical microscopy at the ∼10-nm isotropic resolution scale to thin sub-volumes of cells, thus precluding the ability to image organelles that can extend over several microns throughout the whole cell.

Here, we present a new implementation of iPALM/4Pi-SMSN, termed whole-cell 4Pi single-molecule switching nanoscopy (W-4PiSMSN), which extends the imaging capabilities of this technology to whole cells without compromising resolution. W-4PiSMSN allows volumetric reconstruction with 10- to 20-nm isotropic resolution of ∼10-μm-thick samples, a 10- to 40-fold improvement in sample thickness over previous iPALM/4Pi-SMSN implementations (Aquino et al., 2011, Brown et al., 2011, Van Engelenburg et al., 2014, Shtengel et al., 2009). Our approach permits ultra-high resolution 3D imaging of virtually any subcellular structure. To demonstrate this, we image the endoplasmic reticulum (ER), bacteriophages, mitochondria, nuclear pore complexes, primary cilia, Golgi-apparatus-associated COPI vesicles, and mouse spermatocyte synaptonemal complexes. By these examples, we show that W-4PiSMSN opens the door to address cell biological questions that were previously unanswerable.

## Results

### Development of W-4PiSMSN

To realize a system that achieves 10- to 20-nm 3D resolution across the thickness of entire mammalian cells, we expanded on previous iPALM and 4Pi-SMSN developments (Aquino et al., 2011, Shtengel et al., 2009). In these systems, fluorescence emission is collected by two opposing objective lenses and combined to interfere (Aquino et al., 2011, von Middendorff et al., 2008, Shtengel et al., 2009). Depending on the axial position of a molecule, the light will interfere constructively or destructively, as indicated by the brightness of the molecule’s image on the detector. However, molecules at axial positions that differ by multiples of half the wavelength of light lead to the same interference pattern and cause ambiguity in determining their axial positions. This localization ambiguity leads to scrambled images that contain axially shifted image artifacts, known as ghost images, in samples thicker than ∼250 nm. This can be avoided by using not only the brightness but also the z-position-dependent shape of the single-molecule images to determine a molecule’s axial position. To address this, a higher-moment based analysis (Aquino et al., 2011) and analysis of the point-spread function (PSF) eccentricity in a hyperbolic mirrors-modified system (Brown et al., 2011) were developed, which extended the image volume thickness to 700–1,000 nm. However, these methods pose significant drawbacks such as poor localization density because of the highly selective computational processes focusing on subtle features of PSFs. The methods are also susceptible to sample-induced optical aberrations, which change the PSF shape when imaging biological structures deeper in the sample (Burke et al., 2015, von Diezmann et al., 2015). As a result, applications have been restricted to thin structures close to the coverslip (Case et al., 2015, Van Engelenburg et al., 2014, Kanchanawong et al., 2010).

**Figure 1:**
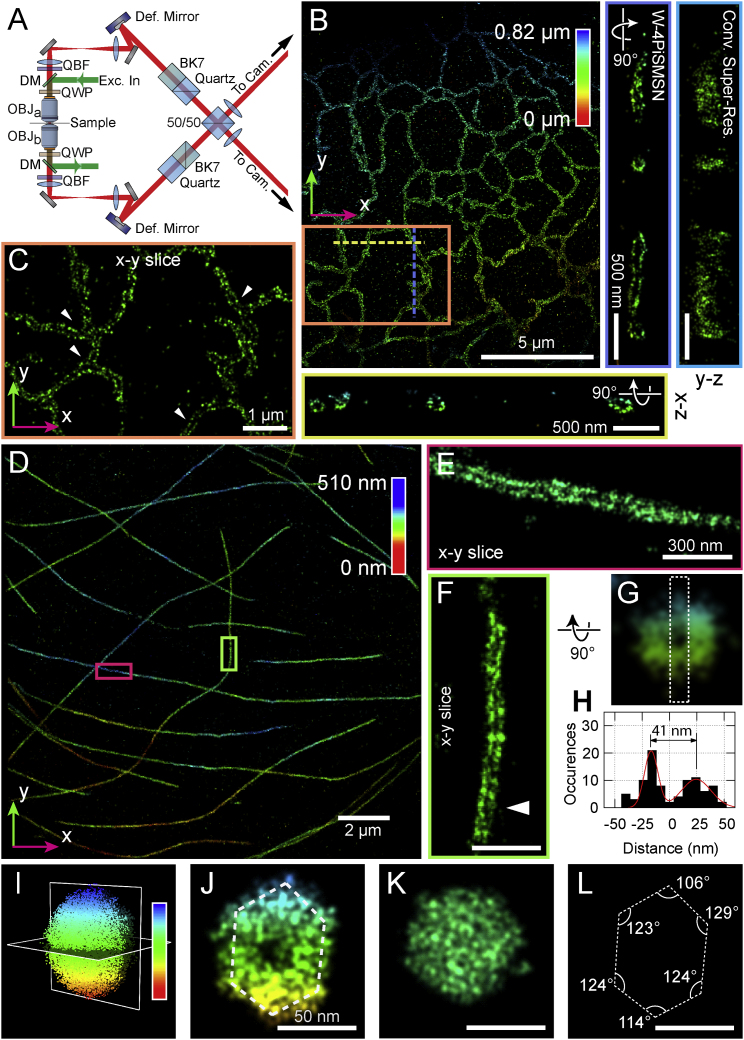
W-4PiSMSN Design and Resolution Demonstrations with ER, Microtubules, and Bacteriophages (A) Simplified optical diagram of W-4PiSMSN. (B) Overview and cross-sections of the ER network in an immunolabeled COS-7 cell. Cross-sections of the W-4PiSMSN reconstruction show clearly separated membranes of the tubular structures, which cannot be resolved with conventional astigmatism-based nanoscopy (light blue frame). (C) x-y slice through the mid-section of the ER network shown in (B) highlights the distinct membrane contour of ER tubules (arrowheads). (D) Overview of immunolabeled microtubules in a COS-7 cell. (E and F) 20-nm-thin x-y slices of the red (E) and green (F) segments shown in (D) demonstrate that microtubules can be easily resolved as hollow cylinders in W-4PiSMSN. (G) A look through a 120-nm-long segment of the microtubule of (F). (H) A histogram showing the number of localizations in a cross-section of the microtubule, white dotted box in (G). (I) A bacteriophage reconstructed from 115 averaged viral particles rendered in 3D. (J and K) 5-nm-thin vertical (J) and horizontal (K) slices through the averaged dataset corresponding to the planes shown in (I). (L) The internal angle measurements of the hexagon shape identified from the viral capsid shown in (J). OBJ, objective; QWP, quarter-wave plate; DM, dichroic mirror; QBF, quad-band band-pass filter; Def. Mirror, deformable mirror; Cam, camera; 50/50, beam splitter cube.

**Figure S1:**
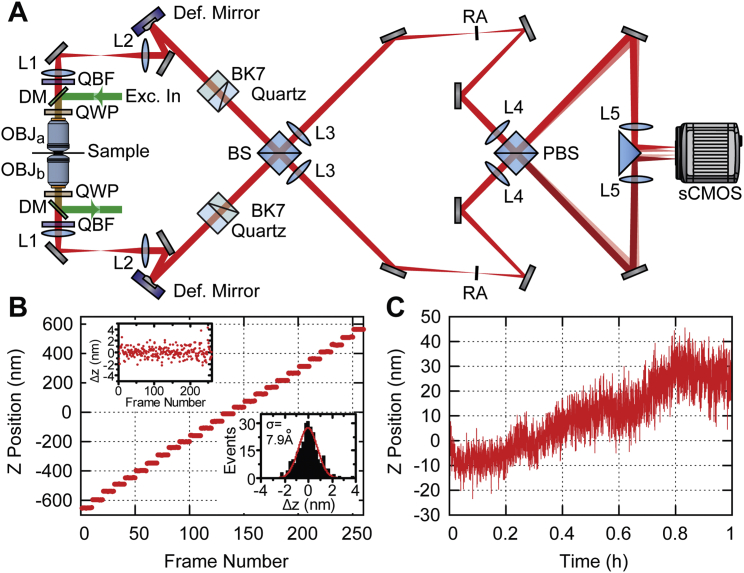
W-4PiSMSN Setup and System Characterization, Related to Figure 1 (A) Simplified system diagram of W-4PiSMSN. (B) Localization results of W-4PiSMSN of a fluorescent bead imaged with 50 nm steps over an axial range of 1.2 μm. Inserts show residual errors displayed for each step and in a histogram. (C) Instrumental drift along the axial direction over 1 hr. L1-L5: Lenses, OBJ: Objective, QWP: Quarter-Wave Plate, DM: Dichroic Mirror, QBF: Quad-Band Bandpass Filter, Def. Mirror: Deformable Mirror, BS: Beam Splitter Cube, PBS: Polarizing Beam Splitter Cube, RA: Rectangular Aperture.

**Figure S2:**
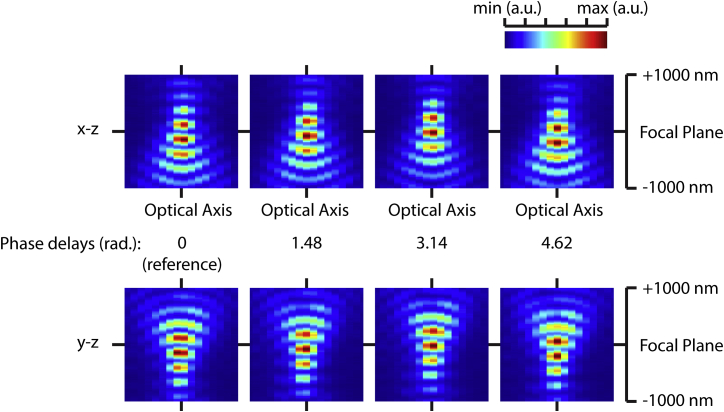
W-4PiSMSN Point Spread Function, Related to Figure 1 Central x-z and y-z sections of W-4PiSMSN point-spread functions in the four images recorded by the sCMOS camera demonstrating interference and astigmatism introduced by the coherent detection cavity and deformable mirrors.

To enable 4Pi-SMSN to probe deeper into the cell and extend the application of this technology to larger cellular features, we have developed W-4PiSMSN. First, expanding on the optical design by Aquino et al. (2011), we included deformable mirrors in both arms of the 4Pi-interferometric cavity (Figure 1A; Figure S1). We use these mirrors to correct for imperfections in the instrument beam path and optimize the PSF quality for samples with various thicknesses (Supplemental Information; Figure S2). Deformable mirrors also allow us to compensate for sample-induced aberration modes, such as spherical aberrations (Burke et al., 2015, Gould et al., 2012b), which vary from sample to sample and with depth. Additionally, we can use these mirrors to introduce astigmatism in both interference arms without adding further complexity to the system (Supplemental Information). Thus, the deformable mirrors enable compromise-free, reproducible PSFs in a depth- and sample-independent manner.

**Figure S3:**
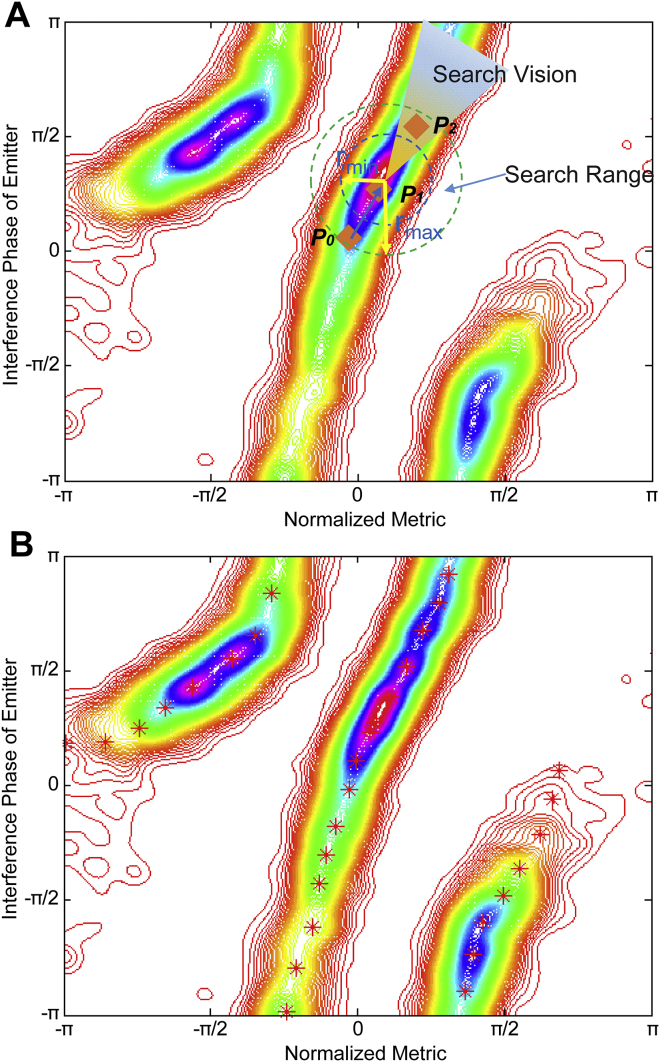
Concept of Ridge-Finding Algorithm, Related to Figure 1 (A) Ridge-finding algorithm concept including demonstrations of vision field, jump range, and directions of the current step. Contour plot of the 2D histogram generated from single-molecule interference phase values and normalized metric values. (B) Identified monotonic ridge of metric versus phase curve before phase unwrapping (red stars).

Second, building on an earlier approach by Brown et al. (2011), we developed an analysis method that combines information from (1) the 4Pi-PSF’s interference phase, which allows for precise axial localization but does not distinguish between different interference peaks, and (2) the eccentricity of the astigmatic 4Pi-PSF, which narrows axial localizations down to individual interference peaks but in itself does not offer the precision of 4Pi interference. Our new analysis algorithm interprets the large number of molecules imaged in each time and z-depth window as an *ensemble measurement* of the concurrent state of the W-4PiSMSN system (Supplemental Information; Figure S3A) and determines the relationship between the eccentricity of the astigmatic PSF and the interference phase of the 4Pi-PSF. Then the axial positions of all corresponding molecules can be assigned with high precision and unambiguously using a monotonic metric designed to describe the overall shape of the PSF and maintain its monotonicity in the presence of moderate amounts of aberrations (Supplemental Information; Figure S3B). Since this analysis is performed for well-defined temporal and axial data subsets, we have generalized it to identify and correct for drift (from both the system and the sample) over the course of imaging. Our method is robust against aberrations and improves the reliability and efficiency of axial position assignment as it automatically adapts to changes in the shape and interference pattern of the 4Pi-PSF.

### Ultra-High Resolution Imaging with W-4PiSMSN

**Figure S4:**
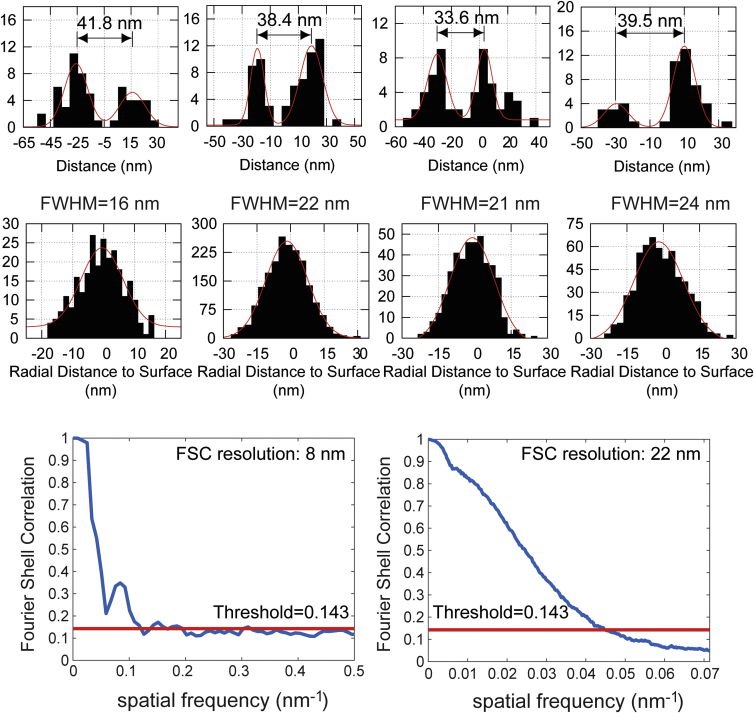
Line Profiles, Residue Plots, and Fourier Shell Correlation Resolution, Related to Figure 1 Top: four line profiles across x-y slices of microtubules shown in Figures 1D–1F. Middle: residual distances from single-molecule localizations to cylinder surface fit to four segments of the microtubule data. Bottom: Fourier shell correlation (FSC) measurement of resolution in a sub-region of ER data (right) (Figures 1B and 1C) and the combined phage data (left) (Figures 1I–1L) based on custom-written software extended to 3D from previously demonstrated Fourier ring correlation on SMSN datasets (Nieuwenhuizen et al., 2013).

To demonstrate the resolution capabilities of our new system, we first imaged the ER. ER membranes were stained using anti-GFP antibodies to the overexpressed transmembrane protein, mEmerald-Sec61β, in COS-7 cells. We visualized the ER as a connected network of hollow tubes with 60–100 nm diameters (Figures 1B and 1C; Movie S1). Both horizontal and vertical cross-sections reveal the 3D membrane contour that was previously resolvable only with electron tomography (Frey and Mannella, 2000). This high 3D resolution is quantitatively supported by a Fourier Shell Correlation value of 22 nm (Figure S4) (Nieuwenhuizen et al., 2013). To test our approach on even smaller structures, we imaged antibody-labeled microtubules in COS-7 cells, a gold standard in SMSN (Figures 1D–1H). Without any detectable imaging artifacts, W-4PiSMSN resolves this 25-nm microtubule filament, which appears in all orientations as a hollow core coated with antibodies (Figures 1E–1H). In addition, the dataset features a high localization density of ∼5.5 localization events per 10 × 10 nm^2^ of surface area. Displaying the localization events by their radial distance from the tubule axis shows a Gaussian peak with a full width at half maximum (FWHM) of 16–24 nm (Figure S4). Considering that the use of primary and secondary antibodies adds uncertainty to the actual position of the imaged dye molecules, we conclude that the 3D resolution of our instrument is well below 20 nm (FWHM).

**Figure S5:**
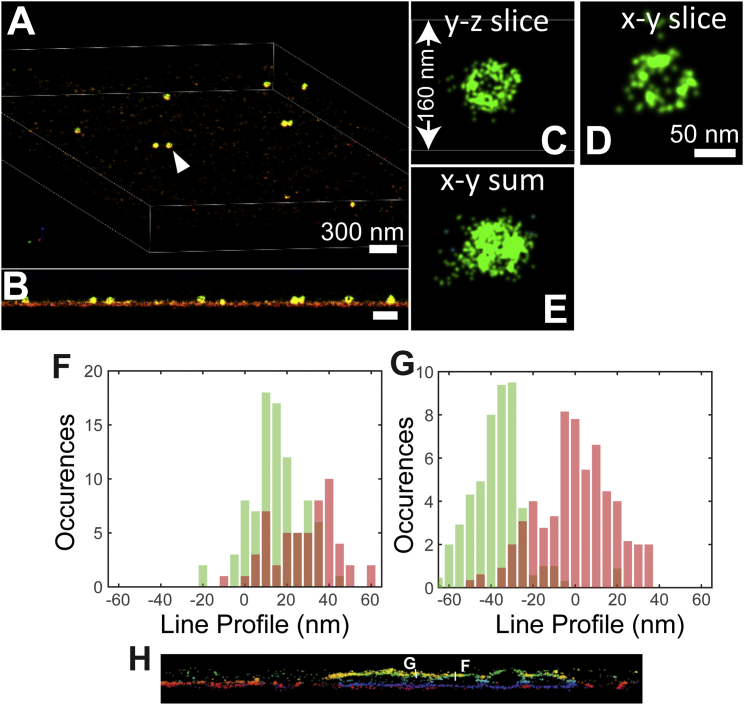
Overview of an Individual Phage and Examples of Line Profiles from Figure 2, Related to Figures 1 and 2 (A) Overview of Alexa Fluor 647 labeled phages imaged by W-4PiSMSN. (B) x-z view of the entire sample showing coverslip surface and individual phages. (C–E) Cross-sections and projection of an isolated phage (arrowhead in A). (F and G) Examples of line profiles (integrated over a width of 200 nm) of the two-color image shown in Figure 2. (H) Line profile positions in Figure 2C.

To demonstrate our approach on another challenging target, we imaged T7 bacteriophages. They feature an icosahedral-shaped capsid of ∼60-nm diameter, which has only been visible by cryo-electron microscopy (cryo-EM) techniques before (Hu et al., 2013). We non-specifically labeled proteins on the surface of purified T7 phages using an Alexa Fluor 647 NHS Ester, which reacts with primary amines, and mounted the phages on a coverslip (Figure S5). Image slices of a single phage in the x-y, y-z, and x-z directions show a hollow center in all orientations. To further refine the details of the detected phage structures, we adapted the tomogram-averaging approach originally developed for cryo-EM (Briggs, 2013, Broeken et al., 2015). By combining 115 T7 images, our averaged reconstruction reveals the icosahedral shape of the T7 phages (Figures 1I–1L; Figure S4). As presented in Figures 1J–1L, a slice perpendicular to the major axis shows the expected pentagonal shape while a slice parallel to the major axis reveals a hexagonal shape. Our approach, however, has yet to clearly resolve the ∼23-nm tail and fiber structures of the T7 phage (Hu et al., 2013). This is likely due to either incomplete labeling of the surface proteins or the flexibility of these structures. Nonetheless, our W-4PiSMSN system has enabled the visualization of the ultrastructure of bacteriophages using light microscopy.

**Figure 2:**
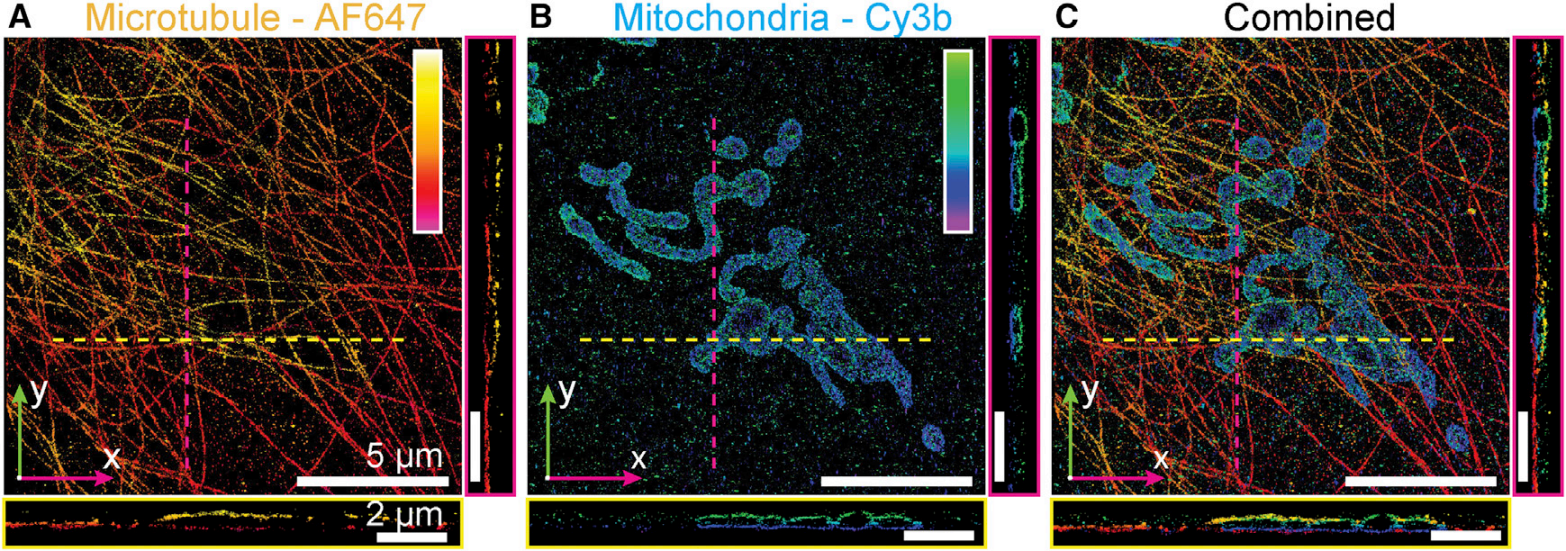
Two-Color Reconstruction of Mitochondria and Microtubules (A and B) W-4PiSMSN reconstruction of microtubules (A) and mitochondria (TOM20) (B) in a COS-7 cell immunolabeled with Alexa Fluor 647 and Cy3B, respectively. An x-y overview and x-z and y-z slices (yellow and magenta lines, respectively) are shown. (C) The combined two-color image reveals microtubules running adjacent to the mitochondria surface.

We tested the two-color imaging capability of W-4PiSMSN by imaging microtubules and mitochondria in a COS-7 cell immunolabeled with Alexa Fluor 647 and Cy3B, respectively. Our reconstructions show microtubules running in close proximity ∼10–20 nm from the mitochondria top and bottom surfaces (Figures 2A–2C; Movie S2; Figure S5). Our system decouples axial localization from the PSF shape, the latter being susceptible to depth-dependent distortions caused by sample-induced optical aberrations (Liu et al., 2013, McGorty et al., 2014). While single-objective systems rely on the PSF shape, the W-4PiSMSN approach uses the relative interference amplitudes to determine the axial location of individual molecules. However, multicolor imaging is challenging because the spatial interference modulation frequency is wavelength dependent and differs between color channels. To address this, we derived the modulation frequency using a pupil-function based approach (Supplemental Information). Our simulation results were verified experimentally by registering two color channels from an affine transformation matrix, which was calibrated using two-color-labeled mitochondria in fixed cells (Supplemental Information).

### Whole-Cell 3D Imaging with W-4PiSMSN

Imaging volumes thicker than ∼1.2 μm requires axial sample scanning, because molecules more than ∼600 nm out of focus cannot be identified and localized efficiently (Huang et al., 2008, Juette et al., 2008). Thus, optical sections must be recorded at different axial sample positions and subsequently merged to obtain the complete cellular volume. Compared to conventional 3D nanoscopes, the superior localization precision of W-4PiSMSN puts high demands on the localization accuracy in each volume section (i.e., avoiding volume distortions) and the merging process. In the section-merging process, small misalignments of neighboring optical sections caused by sample-induced aberrations or drift can lead to significant deterioration of the resolution and distortions of the super-resolved volume (Huang et al., 2008, Mlodzianoski et al., 2011).

**Figure S6:**
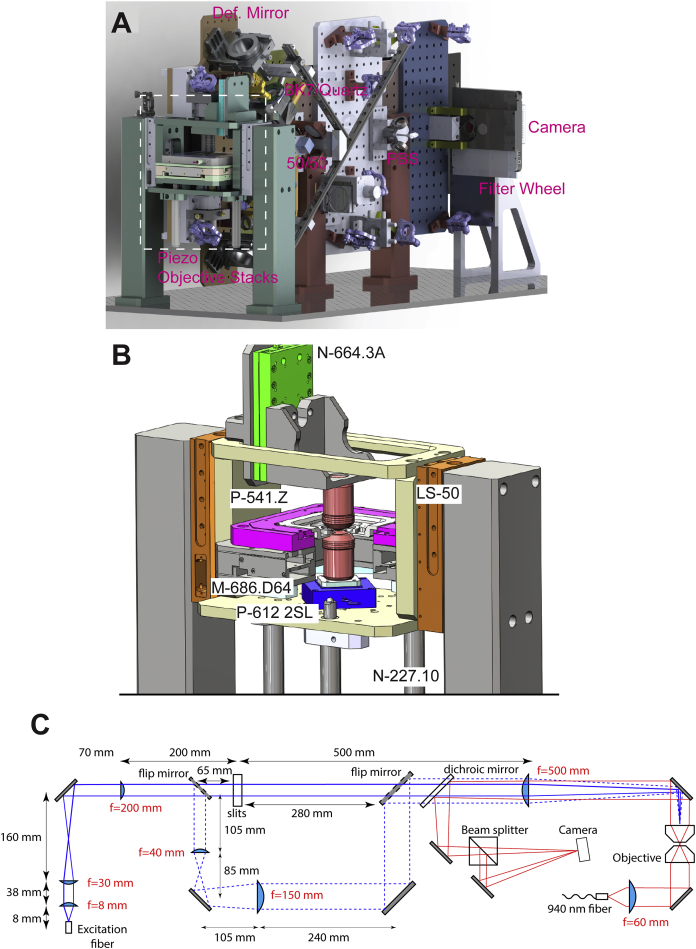
Instrument CAD Renderings and Layout of the Excitation and Diagnostic Beam, Related to Figure 1 (A and B) CAD renderings of the W-4PiSMSN instrument and the piezo-objective stack. 50/50: 50/50 beam splitter cube, PBS: polarizing beam splitter cube, Def. Mirror: deformable mirror. Please see Movie S7 for animation and more details. (C) Excitation and diagnostic beam layout. The excitation light from a polarization-maintaining single-mode fiber (solid blue line) is first collimated by an aspheric lens (f = 8 mm) and further expanded ∼6.6X to a size of ∼12 mm. This beam passes through a pair of square apertures of ∼5x5mm that cropped the center-most uniform part of the beam. An f = 500 mm lens focuses the cropped beam to the back focal plane of the top objective, uniformly illuminating an ∼18x18 μm area in the focal plane. For overview, a pair of flip mirrors route the beam through an alternative path (dashed blue line) that bypasses the apertures. The overview beam is further expanded ∼4X before being focused by the f = 500 mm lens to the back focal plane of the objective and illuminates a ∼100-μm diameter area in the focal plane. To lock the relative position of the two objectives, the laser light from a 940 nm diode laser (red solid line) is collimated by a lens to overfill the back focal plane of the bottom objective, which focuses the light to a spot in the common focal plane. This focus is imaged by the top objective producing a collimated beam propagating in the opposite direction of the excitation light. The f = 500 mm lens focuses the beam through a biplane geometry to a camera.

We designed our system to minimize drift: our instrument design takes advantage of a horizontal symmetry plane coinciding with the common focal planes of the objectives and the beam splitter cube of the interference cavity. This symmetric design desensitizes the interferometric cavity of the microscope to temperature changes leading to approximately equal thermal expansion in both arms of the interference cavity. To compensate for any remaining instrument and sample drift caused by mechanical and thermal fluctuations, we developed a set of hardware and software tools (Figure S6). The objectives are stabilized in 3D relative to each other by focusing a near-infrared laser beam by one objective and detecting the focus with the other objective in a “biplane” configuration (Figure S6) (Juette et al., 2008, Ram et al., 2008). This allows the detection of relative objective movement in 3D, which can then be compensated for via a feedback loop. Furthermore, we cross-correlate 3D volume data segments of 1- to 2-min windows using a redundancy-based drift correction method (Li et al., 2013, Wang et al., 2014) in an extended correlation volume. Within each of these short data segments, an independent relationship between astigmatism and interference phase is established. Any discrepancies between these relationships for different segments are treated as drift (Supplemental Information). The above-described methods enable us to fully compensate for sample and instrument drift and changes in the optical path between the two arms of the interferometric 4Pi cavity due to the axial scanning nature of the measurements.

**Figure 3:**
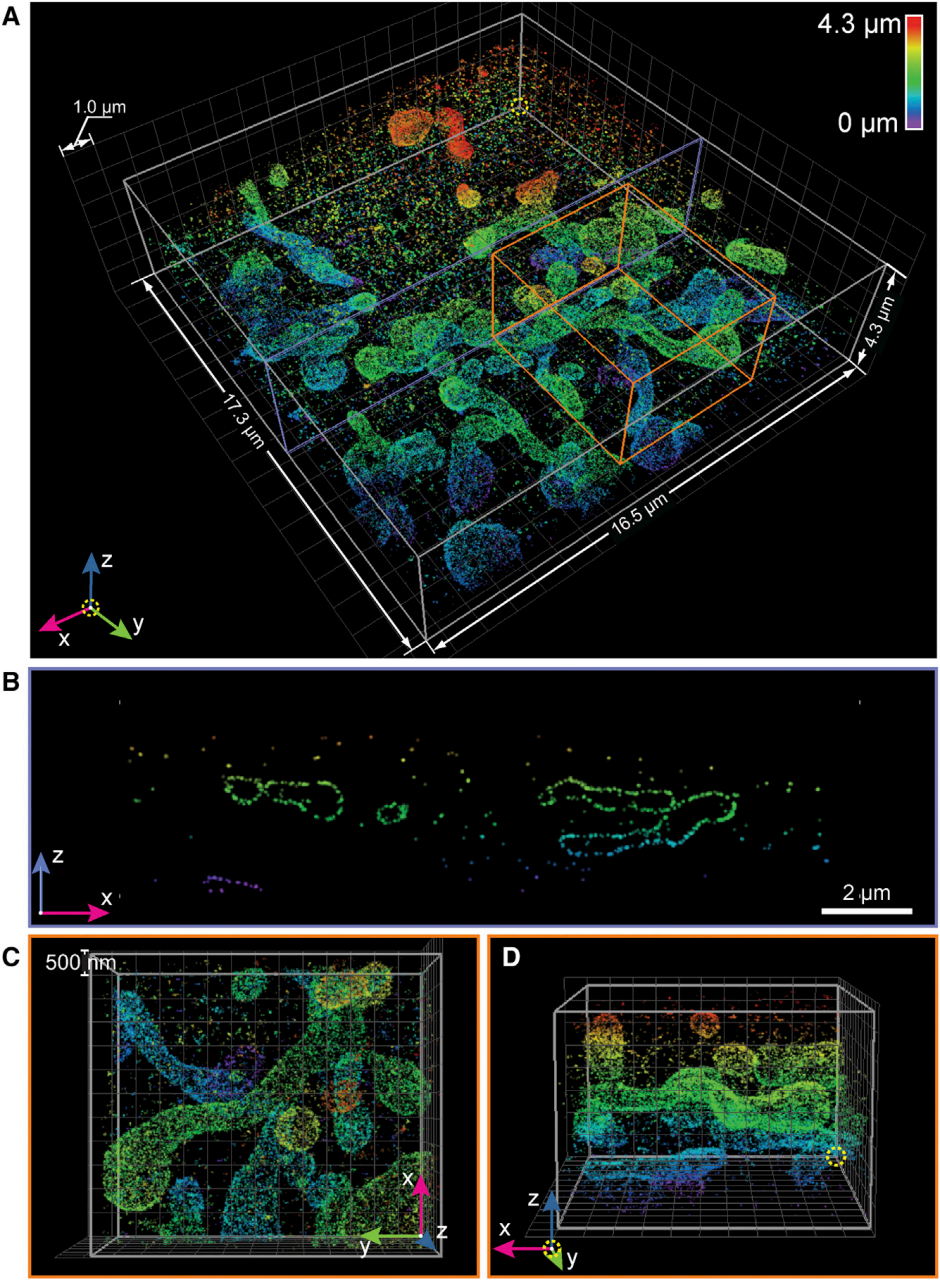
W-4PiSMSN Reconstruction of TOM20 on Mitochondria in COS-7 Cell (A) Overview of the mitochondria network visualized by immunolabeling TOM20 with Alexa Flour 647. The dataset is assembled from 11 optical sections with 500-nm step sizes. (B) x-z cross-section of the purple plane in (A) showing the distribution of TOM20 on the outer mitochondrial membrane. Ghost images are completely negligible. (C and D) Top (C) and side (D) views of the orange box in (A) show the 3D arrangement of the organelle.

To demonstrate the whole-cell imaging capabilities of the W-4PiSMSN system, we imaged mitochondria using antibodies against the outer membrane protein TOM20 over the whole thickness of a COS-7 cell. Figure 3 reveals the outer membrane contour and the remarkably interconnected mitochondrial network over a depth of 4.3 μm (Figures 3B–3D; Movie S3). We were not able to detect any significant ghost images within the volume (Figures 3A–3D).

**Figure 4:**
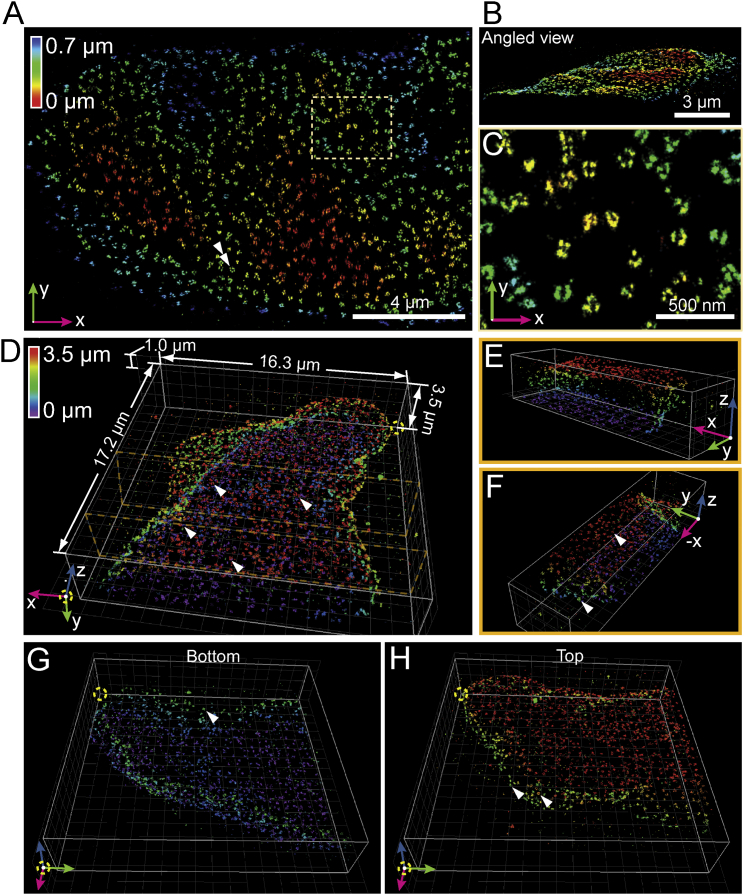
W-4PiSMSN Imaging of Nuclear Pore Complexes over the Thickness of a Cell Nucleus Nup358 was Immunolabeled with Alexa Fluor 647 in hTERT-RPE1 cells. (A) Overview of a region of the nucleus. The axial location of the nuclear pores is color coded. (B) Side view of (A). (C) A subregion indicated by the dashed box in (A) shows a zoomed in view of multiple nuclear pores. (D) Overview of a 3D reconstruction of the nucleus obtained by combining nine optical sections. (E) A section of the reconstruction in (D) confirms that the labeling is largely limited to the nuclear envelope. (F) Different view of the same section. (G and H) Bottom (G) and top (H) half of the nucleus shown in (D). The images reveal ring-like nuclear pores on the top and the bottom nuclear envelope as well as at the sides of the nucleus (arrowheads).

To further demonstrate that image quality is maintained throughout the thickness of whole cells, we imaged nuclear pore complexes (NPCs) on the nuclear envelope. By immunolabeling with an antibody that recognizes a component of the cytoplasmic filaments (Nup358) of NPCs (von Appen et al., 2015), we can reconstruct NPCs on the top, side, and bottom of the nucleus (Figure 4; Movie S4). As with mitochondria, our approach reveals the contours of almost the entire nuclear surface, where both prominent invaginations and subtle undulations (typically visualized only by electron microscopy [EM]) are apparent (Figures 4A and 4B).

### Revealing Golgi-Apparatus-Associated COPI Vesicles

**Figure 5:**
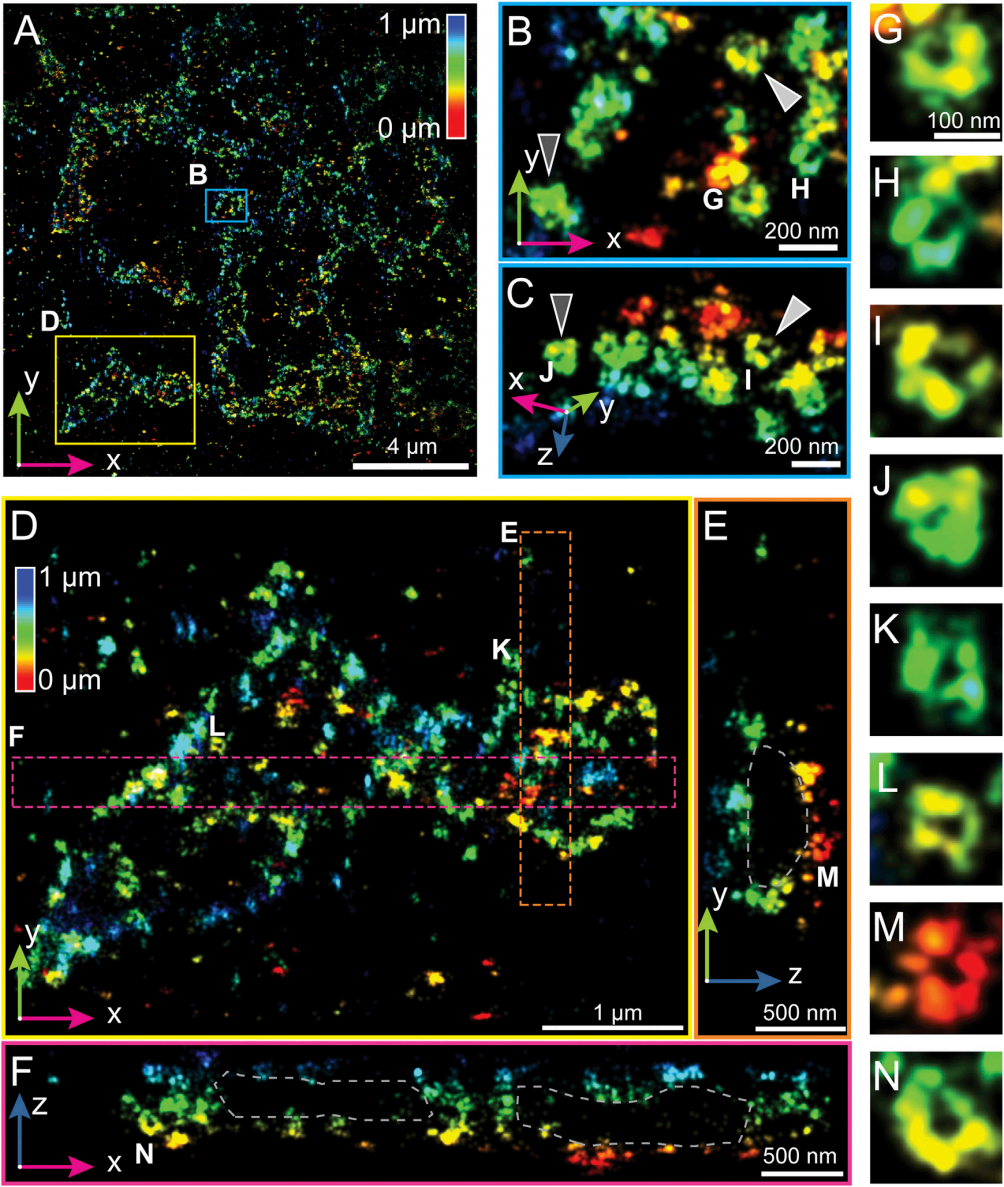
W-4PiSMSN Resolves Individual COPI-Coated Vesicles COPI complexes were immunolabeled with an antibody against β′ COP and imaged with Alexa Fluor 647 in BSC-1 cells. (A) Overview of a region of the field of view, with axial location of molecules color coded. (B and C) Top (B) and side (C) view of the blue-boxed subregion indicated in (A) showing that COPI often forms round and hollow sphere-like structures. Dark gray and light gray arrowheads indicate the same COPI structures. (D) x-y view of the area devoid of COPI as indicated by the yellow box in (A). (E and F) x-z and y-z view of the orange (E) and magenta (F) boxed regions shown in (D) show that COPI surrounds an area presumably containing the Golgi cisternae. (G–N) COPI vesicle structures as indicated by the respective labels in (B)–(F) shown at the same enlarged scale reveal circular structures.

We next imaged COPI vesicles, which have traditionally been resolved only by EM as they have ∼100-nm diameters and are densely packed around the Golgi cisternae (Orci et al., 1997). Moreover, as the Golgi complex is located close to the middle of the cell, recording high-quality data in a central z-plane is a challenging test of the instrument’s 3D resolution capabilities. Figure 5 shows the β′ COP, a protein in the outer COPI complex, immunostained using Alexa Fluor 647 in BSC-1 cells. Strikingly, we visualized distinct hollow COPI-coated spheres within cells (Figures 5B, 5C, 5E, 5F, and 5G–5N). Our 3D images resolve individual COPI vesicles with ∼100-nm diameter, consistent with previous measurements (Pellett et al., 2013). Additionally, a 300-nm-thick section shows that COPI-coated structures are packed around a 500- to 1,000-nm (x and y) by 500-nm (z) area devoid of COPI labeling, presumably containing a Golgi stack (Figures 5D–5F).

### Revealing Ciliary Membrane GPCR Organization

**Figure 6:**
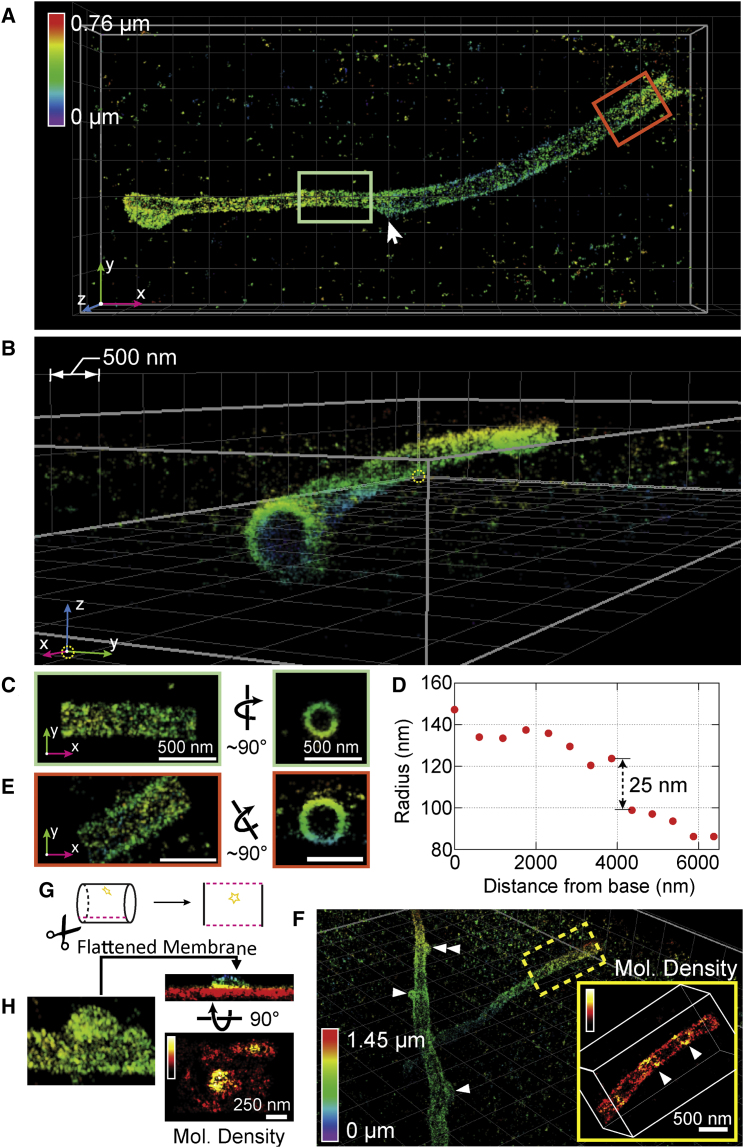
GPCR Smoothened on a Primary Cilium (A and B) Top (A) and side (B) views of a primary cilium on an hTERT-RPE1 cell expressing pH-SMO, which was immunolabeled with Alexa Fluor 647. (C and E) Views of sections close to the tip (C) and the base (E) as indicated by the light green and orange boxes in (A) show the localization of pH-SMO to the cilium membrane. (D) Radius of different sections of the cilium as a function of their distance from the base. (F) Overview of a cilium in another region of the sample, showing vesicle-like buds on the ciliary membrane surface (arrowheads). The inset shows the local density of the boxed region, which suggests a helical stripe organization of pH-SMO (arrowheads in inset). (G and H) A bud-like profile shown in (F) can be unwrapped as depicted in (G), showing the height of the vesicle above the cilium membrane and the high molecular density of pH-SMO at the bud (H).

**Figure S7:**
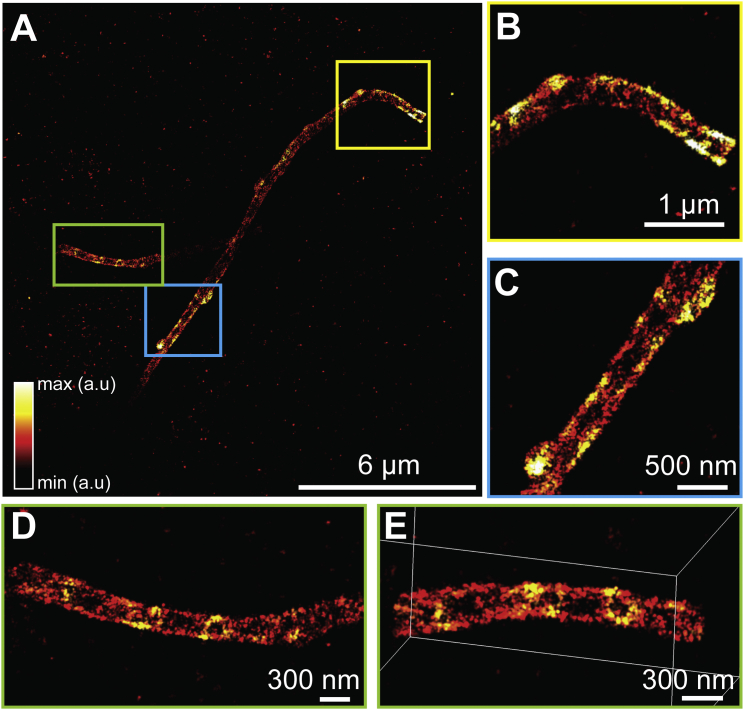
Molecular Density of the Cilia Membrane Protein GPCR Smoothened, Related to Figure 6 (A) Overview of a cilium, color-coded by molecular density. Density was calculated by counting the number of localizations surrounding each localization within a 100-nm radius. (B–E) Zoomed and rotated views show increased molecular density at the base of the cilium (B), at positions with potential budding vesicles (C), and in bands along the length of the cilium (D and E) which suggest potential functional arrangements of SMO.

Most high-resolution studies of the primary cilium, a solitary microtubule-based organelle that protrudes from the cell surface and acts as a cellular antenna, have relied on EM (Wood and Rosenbaum, 2015). A transmission EM image typically shows only a small subsection of a cilium as the sample is a random oblique ∼70- to 100-nm-thick section through the structure, which can be up to 10 μm long and ∼250 nm wide. Scanning EM images can easily show an entire cilium with high resolution; however, these images completely lack information about specific protein localization. Previous nanoscopy studies on cilia relied on inferring the 3D organization from 2D datasets (Yang et al., 2013, Yang et al., 2015). Here, we used W-4PiSMSN to image the G-protein-coupled receptor Smoothened (SMO) on whole primary cilia in hTERT-RPE1 cells with high 3D resolution (Figure 6; Movie S5). SMO was tagged with a pH-sensitive GFP (pH-SMO), which was used as an epitope for antibody labeling with Alexa Fluor 647 (Figure 6; Supplemental Information). We observe that overexpressed pH-SMO localizes to the membranes of cilia, which form hollow cylinders 3–10 μm long (Figure 6) and vary in diameter from ∼160 to 280 nm (Figures 6A–6E). Our W-4PiSMSN images of the ciliary membrane allow us to precisely measure the cilium’s diameter along its entire length. Interestingly, we find that cilia diameters are not always constant. Rather, one example cilium shows an abrupt contraction of ∼50 nm midway along its length (Figures 6C–6E; Movie S5). We speculate this change in diameter may correspond to the thinning of the 9+0 microtubule axoneme, which is known to transition from triplet microtubules, to doublets, and eventually singlets. The ciliary tip is not narrow but has a bulbous shape, consistent with structures observed in EM (He et al., 2014, Wang et al., 2013). The high-resolution reconstruction of the ciliary membrane also allowed us to “unwrap” the membrane tube into a flat surface (Figure 6H; Supplemental Information) permitting data quantification such as cluster analysis and co-localization measurement in a complex geometry. Next, we examined the local density of molecules along the ciliary membrane to identify regions with higher concentrations of pH-SMO. Higher local density is present around the base, on small bulbous protrusions, and on stripes along the cilium length (Figures 6F–6H; Figure S7). These protrusions may be vesicles (diameter ∼150–200 nm) budding from the cilium (Figures 6F–6H), as ectosomes have been reported to bud from some cilia (Wood and Rosenbaum, 2015).

### Resolving Synaptonemal Complexes in Whole-Mouse Spermatocytes

**Figure 7:**
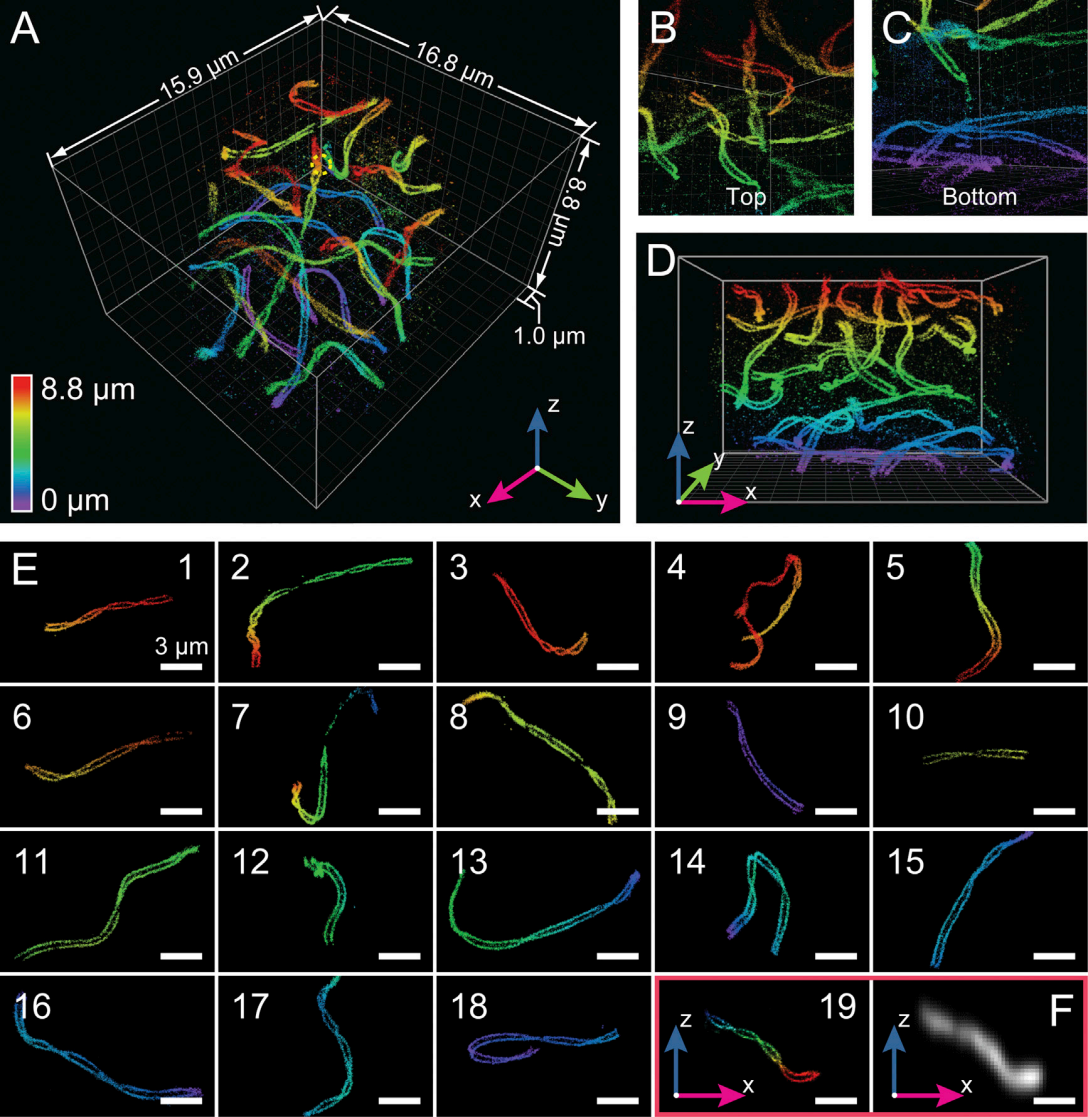
W-4PiSMSN of the Synaptonemal Complexes in a Whole-Mouse Spermatocyte (A) Overview reconstructed from 21 optical sections. Lateral elements of the synaptonemal complex, spaced ∼200 nm apart, are resolved throughout the ∼9-μm depth of the spermatocyte at uniform resolution. (B and C) Different views from locations inside the spermatocyte centered on top (B) and bottom (C) regions of the dataset. (D) x-z view of (A). (E) The 19 synaptonemal complexes from an entire mouse spermatocyte haploid genome were computationally isolated using a Euclidian distance-based clustering algorithm. (F) A conventional image of the 19^th^ synaptonemal complex in x-z view. Scale bars in (E-19) and (F), 2 μm.

As a final demonstration of the capacity of our instrument to image deep into cells as thick as 10 μm, we stained synaptonemal complexes in mouse spermatocyte nuclei in the pachytene phase of meiotic prophase (Figure 7; Movies S6). While synaptonemal complexes have been imaged using structured illumination (Carlton, 2008, Qiao et al., 2012) and 4Pi microscopy at 100- to 200-nm resolution (Fritsche et al., 2012), higher resolution optical images have been limited to chromosome spreads of <1-μm thickness (Schücker et al., 2015). Here, we examined spermatocytes harvested from testes of 17- to 18-day-old mice with W-4PiSMSN and imaged the twisting band of the paired lateral elements of autosomal synaptonemal complexes, highlighted by immunolabeling synaptonemal complex protein 3 (SYCP3), a constituent component of the lateral elements (Page and Hawley, 2004). Reconstructed from a total of 126 optical sections (21 depth positions imaged in six repetition cycles), the entire 3D image spanned nearly 9 μm in depth and resolved SYCP3 substructure of the individual autosomal synaptonemal complexes with unprecedented clarity independent of their orientation or depth (Figures 7A–7E; Movie S6). Furthermore, 19 synaptonemal complexes representing pairs of individual autosomal homologs could be isolated using a Euclidian metric-based clustering algorithm on the individual single-molecule localizations (Supplemental Information). Thus, our approach promises the capacity to visualize the nanoscale spatial organization of chromosomal scaffolds in the context of architectural elements of the nucleus, many of which are lost in commonly used spread chromatin preparations.

## Discussion

Through a confluence of several technological innovations, we have demonstrated that W-4PiSMSN provides unprecedented access to the ultrastructure of cells with ∼10- to 20-nm isotropic resolution throughout their entire volume. This resolution is 20–50 times higher than conventional microscopy with imaging depth improved ∼10-fold over state-of-the-art iPALM and 4Pi-SMSN. This development extends the application range of 4Pi-based SMSN dramatically: imaging is no longer limited to features within small sub-volumes of cells. Instead, we are capable of imaging organelles that span large volumes, exemplified by the mitochondrial network, the nuclear envelope, and synaptonemal complexes, which we capture in virtual entirety. Thus, W-4PiSMSN is a versatile and powerful tool that promises a new perspective on how proteins distribute across entire organelles throughout whole cells, a key unmet challenge in cell biology.

Is there room to further increase the spatial resolution of SMS nanoscopy? First, SMS resolution depends on the precision with which one can localize blinking molecules. The precision is approximately proportional to the sharpness of the PSF and, for negligible background noise, is inversely proportional to the square root of the number of detected photons. Our approach has focused mainly on creating the sharpest PSF and detecting as much of the emitted fluorescence light as possible. Recently, there have been promising developments that increase the number of emitted photons per molecule (Klehs et al., 2014, Ong et al., 2015, Vaughan et al., 2012), which we have not exploited here. Unfortunately, these advances have so far come at the expense of an increase in recording time. We anticipate, however, that with new generations of fluorophores or refined imaging buffers, these approaches can further improve image quality.

Second, image quality, or spatial resolution, of SMSN images depends on the density of localized molecules (Patterson et al., 2010, Shroff et al., 2008). The application examples we presented demonstrate that small features such as cylinder-shaped immunolabeled microtubules (∼40-nm diameter) or ER tubules (∼60-nm diameter) can now be easily resolved in 3D using light microscopy. This image interpretation is aided by the fact that the observer fills the gaps in the distribution of molecules along a tubule by mentally extrapolating from the expected tubular structure. We utilized several computational image processing techniques, including particle averaging of the bacteriophage data (Figure 1), clustering of the synaptonemal complexes (Figure 7; Movie S6), and “unwrapping” of cilia (Figure 6), to reconstruct complex structures. These approaches, which add constraints to data interpretation (e.g., the fact that cilia are tubular) and can be tailored to the cell biological question at hand, allow us to extract structural details, which are more subtle than the labeling density suggests. Ultimately, labeling density is limited by the density of probe targets, usually proteins. The development of new labeling approaches that allow membrane targeting (Erdmann et al., 2014) overcome this restriction and additionally offer the possibility of revealing the membrane boundaries of individual organelles. Complementary approaches utilizing stochastic transient chemical binding can further address the limited pool of fluorescent labels and theoretically allow unlimited numbers of localized molecules (Giannone et al., 2010, Jungmann et al., 2014, Lew et al., 2011, Sharonov and Hochstrasser, 2006). However, even with the use of conventional labeling techniques, W-4PiSMSN is capable of visualizing otherwise inaccessible structures in a multitude of settings as demonstrated by the large range of presented applications.

In conclusion, we believe that the development of W-4PiSMSN represents the culmination of more than a decade’s research on high-resolution fluorescence imaging techniques and establishes 3D biological imaging with molecular specificity and resolution in the 10-nm range as a general imaging technique.

## Experimental Procedures

### Microscope Setup

A detailed description is provided as Supplemental Information. In brief, the 4Pi cavity of the system was set up vertically around two opposing high-NA objective lenses (Movie S7). Excitation light from three laser lines at wavelengths of 642, 561, and 405 nm was coupled into the upper objective for wide-field illumination. Following the concept by Aquino et al. (2011), fluorescence was collected by both objectives and passed through quarter wave plates, which enforced equal fractions of *s* and *p*-polarized light independent of the dipole emitter orientation. A custom Babinet-Soleil compensator corrected for dispersion and allowed adjusting the phase delay between the upper and lower interferometer arm independently for the two polarization components before the light was combined at a 50/50 beam splitter cube. We added deformable mirrors (Boston Micromachines, Multi-5.5) in planes conjugate to the back pupils of the objectives, which allowed for aberration correction, optimization of the PSF and introduction of astigmatism for artifact-free 3D localization. *s* and *p*-polarized fluorescence exiting the beam splitter cube at two sides was split with a polarizing beam splitter cube into four images featuring different interference phases. The four images were recorded simultaneously by a scientific complementary metal-oxide semiconductor (sCMOS) camera (Hamamatsu, ORCA-Flash 4.0v2). The entire system was controlled by a custom-written program in LabVIEW.

### Aberration Correction Using Deformable Mirrors

Two deformable mirrors were independently adjusted as follows. For each interfering arm, starting from the flat voltage map (provided by the manufacturer), 28 theoretically generated membrane modes were applied sequentially with ten different voltage amplitudes per mode. The detected peak signals (0^th^ moment Gaussian weighted sum) of a single fluorescent bead in focus were extracted for all amplitudes of the applied modes. The optimal amplitude for each mode was determined as the value that gave the maximum signal level by fitting a quadratic function to the measurements. These newly obtained amplitudes were added to the flat voltage map and serve as a starting point of another iteration. We used five iterations to achieve optimal system aberration correction. Details are provided the Supplemental Information.

### Biological Sample Preparation

A complete description of cell culture, fluorescence labeling, coverslip, and buffer preparation is included in sections 15 to 26 of the Supplemental Information.

In short, 25-mm-diameter coverslips were cleaned by sonication in 1 M KOH for 15 min before use. Fluorescent 100-nm-diameter crimson beads were attached to the coverslip surface using poly-L-lysine. Cultured mammalian cells were grown on coverslips for 24–48 hr before fixation. COS-7 cells were used for microtubule, ER, and mitochondria samples. RPE-hTERT cells were used for nuclear pore complex and cilia samples. BSC1 cells were used for COPI samples. For synaptonemal complex samples, spermatocytes were isolated from the testes of mice and settled on coverslips before being fixed. T7 bacteriophages were isolated from *E. coli* cultures and labeled with Alexa Fluor 647 NHS Ester before being placed on coverslips.

Microtubule, mitochondria, and ER samples were fixed with 3% paraformaldehyde (PFA) + 0.1% glutaraldehyde before antibody labeling. A saponin pre-extraction step preceded fixation when only microtubules were labeled. Nuclear Pore Complex samples were fixed in −20°C methanol. COPI and synaptonemal samples were fixed in 4% PFA.

Antibodies against endogenous proteins were used to label microtubules (anti-α-tubulin), mitochondria (anti-TOM20), nuclear pore complexes (anti-Nup358), COPI (anti-β′ COP), and synaptonemal complexes (anti-SYCP3). Overexpressed proteins were labeled with antibodies in ER samples (mEmerald-Sec61β using anti-GFP) and Cilia samples (pHlourin-mSmoothened using anti-GFP).

All one-color samples were labeled with Alexa Fluor 647, either using a commercial secondary antibody or an NHS ester. Two-color microtubule and mitochondria samples were labeled with Alexa Flour 647 and Cy3B, respectively. Cy3B-labeled secondary antibodies were made by conjugating reactive Cy3B with unlabeled antibodies. After secondary antibody labeling, COS-7 and BSC1 cell samples were post-fixed with 3% PFA + 0.1% GA.

Labeled biological samples were placed in an aluminum sample frame and covered with a second cleaned coverslip. A thin spacer and imaging buffer was placed between the two coverslips. The coverslips were held in the sample frame using an addition-curing silicone. The imaging buffer was either conventional or COT containing thiol buffer (Supplemental Information). Samples were imaged immediately after the silicone solidified.

### W-4PiSMSN Data Acquisition

Four phase images are arranged along the splitting line of the sCMOS camera’s upper and lower readout region. 50,000 to 320,000 camera frames were recorded at 50 or 100 fps, resulting in 10 min to 1.5 hr total acquisition time per dataset. For sample volumes thinner than 1.2 μm, the sample stage was not translated during data acquisition. For thicker samples, the stage was moved axially in 500-nm steps every 1,000–3,000 frames until the whole targeted imaging volume was covered, resulting in up to 21 z-steps and imaging volume depths of up to 9 μm. This axial scan was automatically repeated six to 19 times, and the data from the scans were combined. The laser intensity was manually adjusted during each experiment to optimize the emitter density per frame and to maximize detectable emissions starting at intensities as high as 35 kW/cm^2^ to transfer emitters efficiently into dark states and decreasing to typically 5 kW/cm^2^ near the end of the acquisition when the pool of blinking molecules had declined (Lin et al., 2015). Additionally, the laser intensity of the 405-nm laser was manually controlled over the course of imaging to optimize the active emitter density.

### Single-Molecule Interference Phase Estimation

Raw camera frames were first isolated into four different phase images. The four phase images were then merged into a single image using a transformation matrix obtained from a combination of algorithms using log-polar and affine transformations (Supplemental Information). Single-molecule candidates from the merged frames were isolated and fitted with an elliptical Gaussian model using a maximum likelihood estimator accounting for the camera-specific noise associated with sCMOS cameras (Huang et al., 2013) (Supplemental Information). Estimates of single-molecule positions, width, total number of detected photons, background, and log-likelihood ratio were obtained. Intensities of the 0^th^ moment Gaussian (Supplemental Information) were calculated by a weighted least-square fitting of a Gaussian with the amplitude being the only fitting parameter and weighted to take the sCMOS noise into account (Huang et al., 2013). Subsequently, the reduced moments of each polarization were extracted using a previously described approach (Aquino et al., 2011). For two-color imaging, phase shifts between *s* and *p* polarization for the two color channels differ by 0.3 radians. These phase shift differences were measured independently from images of fluorescent beads recorded in two detection channels. With the obtained phase differences between the *s* and *p* polarization, the phase shifts between the four different phase images are known. Subsequently, the interference phases of the detected molecules were obtained by solving a set of equations describing the 0^th^ moment intensities in *s* and *p* polarization channels as functions of an unknown offset and their relative phase shifts as characterized above (Supplemental Information).

### Axial Position Analysis with Ridge-Finding Algorithm

We developed a metric *m*: $\left(\sigma_{x}^{3}/\sigma_{y}\right)-\left(\sigma_{y}^{3}/\sigma_{x}\right)$, where $\sigma_{x}$ and $\sigma_{y}$ are the estimated SDs of the 2D Gaussian for a single-molecule emission pattern. The metric preserves its monotonicity in the presence of aberrations, and we used it to estimate an unambiguous (but still rough) position of each single emitter, before the phase estimate was used to pinpoint the exact axial position of a molecule.

For every 3,000–5,000 camera frames, we generated a 2D histogram image from phase estimations and the metric *m* from all detected single molecules. As *m* is monotonic and the single-molecule phase is periodic with a period of 2π, the resulting 2D histogram looks like a pattern of tilted repeating stripes (Figure S3). We developed a ridge-finding algorithm to determine a series of connected vectors defining the correspondence between the determined phase values and the values of *m*. This allowed us to unwrap the periodic phase for unambiguous axial localization. A detailed description of the algorithm is provided in the Supplemental Information.

### Drift Correction

3D drift correction was performed by first calculating the distance pairs between image segments (3,000–5,000 frames), and subsequently forming three sets of equations for x, y, and z, respectively (Li et al., 2013, Wang et al., 2014). A least-square solution that minimizes the overall error of the set of equations was obtained and back-substituted into all equations. Errors can be calculated from each of these equations and a specific equation within the set is removed from the stack if its error is larger than 7 nm. This process was repeated until no error was larger than 7 nm or the matrix was no longer at its full rank. This allowed us to correct system and sample induced drift in 3D with short segments of data (Supplemental Information).

### Optical Segment Alignment

To image thick samples, optical sections were recorded at different axial positions of the sample by axially translating the z-piezo holding the sample stage. The localization data contain x, y, and z position estimates of different optical sections and must be aligned/stitched seamlessly to support the high precision obtained in W-4PiSMSN. Previous methods (Huang et al., 2008) that shifted each optical section by a constant in the axial direction have been prone to introduce misalignment of the optical sections and subsequently deteriorate the resolution achievable in thick samples. Here, we developed an optical alignment method based on 3D cross-correlation. In the W-4PiSMSN system, optical sections are ∼1.2 μm thick. Whole-cell samples were scanned in the axial direction with 500-nm step sizes. This allowed for abundant overlapping regions between adjacent optical sections and provided critical information for precise optical section alignment using the developed 3D cross-correlation methods as described in Supplemental Information.

## Author Contributions

F.H., G.S., E.S.A., M.J.B., and J.B. designed the microscope. F.H., G.S., E.S.A., T.P., M.J.B., and J.B. built the instrument and developed the computer code. F.H., G.S., E.S.A., L.K.S., W.C.D., E.B.K., Y.Z., and J.B. optimized and tested the microscope. L.K.S., W.C.D., F.E.R.-M., J.R.M., I.I., M.L., M.A.H., C.J.-W., C.P.L., J.E.R., D.T., and J.B. designed biological experiments. F.H., G.S., L.K.S., W.C.D., F.E.R.-M., J.R.M., I.I., and M.L. optimized sample preparation protocols and prepared samples. F.H., G.S., E.S.A., L.K.S., E.B.K., T.P., and J.B. visualized the data. All authors contributed to writing the manuscript.

## Supplemental Information

Document S1. Supplemental Experimental Procedures

Movie S1. Endoplasmic Reticulum Immunolabeled Overexpressed mEmerald-Sec61β in a COS-7 Cell, Related to Figure 1

Movie S2. Mitochondria and Microtubules Immunolabeled TOM20 and α-Tubulin in a COS-7 Cell, Related to Figure 2

Movie S3. Mitochondria Immunolabeled TOM20 in a COS-7 Cell, Related to Figure 3

Movie S4. Nucleus Immunolabeled Nuclear Pore Complexes in an hTERTRPE1 Cell, Related to Figure 4

Movie S5. Primary Cilium Immunolabeled pHluorin-Smoothened Expressed in an hTERT-RPE1 Cell, Related to Figure 6

Movie S6. Synaptonemal Complex Immunolabeled Synaptonemal Complex Protein 3 in a Mouse Spermatocyte, Related to Figure 7

Movie S7. Animation of the W-4PiSMSN Setup, Related to Figure 1 and Experimental Procedures

Document S2. Article plus Supplemental Information
