# Supplementary material for: Ultra-High Resolution 3D Imaging of Whole Cells
Source: Cell. 2016 Aug 11;166(4):1028–40. doi: 10.1016/j.cell.2016.06.016 (PMC5005454; doi:10.1016/j.cell.2016.06.016)
Supplement: Document S1. Supplemental Experimental Procedures [file mmc1.pdf]

Cell, Volume *166*

Supplemental Information

Ultra-High Resolution 3D Imaging of Whole Cells

Fang Huang, George Sirinakis, Edward S. Allgeyer, Lena K. Schroeder, Whitney C. Duim, Emil B. Kromann, Thomy Phan, Felix E. Rivera-Molina, Jordan R. Myers, Irnov Irnov, Mark Lessard, Yongdeng Zhang, Mary Ann Handel, Christine Jacobs-Wagner, C. Patrick Lusk, James E. Rothman, Derek Toomre, Martin J. Booth, and Joerg Bewersdorf

Supplemental Materials

ULTRA-HIGH RESOLUTION 3D IMAGING OF WHOLE CELLS

Fang Huang[1,2,†], George Sirinakis[1,3†], Edward S. Allgeyer[1], Lena K. Schroeder[1], Whitney C. Duim[1,4], Emil B. Kromann[1,5], Thomy Phan[1], Felix E. Rivera-Molina[1], Jordan R. Myers[1], Irnov Irnov[6,7], Mark Lessard[8], Yongdeng Zhang[1], Mary Ann Handel[8], Christine Jacobs-Wagner[6,7,9,10], C. Patrick Lusk[1], James E. Rothman[1,11], Derek Toomre[1,11], Martin J. Booth[12,13], Joerg Bewersdorf[1,5,11,*]

1. Department of Cell Biology, School of Medicine, Yale University, New Haven, CT 06520, USA
2. Weldon School of Biomedical Engineering, Purdue University, West Lafayette, IN 47907, USA
3. The Gurdon Institute, University of Cambridge, CB2 1QN, UK
4. Department of Chemistry, Harvey Mudd College, Claremont, CA 91711, USA
5. Department of Biomedical Engineering, Yale University, CT 06520, USA
6. Microbial Sciences Institute, Yale University, West Haven, CT 06477, USA
7. Department of Molecular, Cellular and Developmental Biology, Yale University, New Haven, CT 06520, USA
8. The Jackson Laboratory, Bar Harbor, ME 04609, USA
9. Howard Hughes Medical Institute, Yale University, New Haven, CT 06520, USA
10. Department of Microbial Pathogenesis, Yale School of Medicine, New Haven, CT 06520, USA
11. Nanobiology Institute, Yale University, West Haven, CT 06477, USA
12. Department of Engineering Science, University of Oxford, Oxford, OX1 3PJ, UK
13. Centre for Neural Circuits and Behaviour, University of Oxford, OX1 3SR, UK

† These authors contributed equally to the work

*To whom correspondence should be addressed:
Joerg.Bewersdorf@yale.edu

Supplemental Experimental Procedures

1. Microscope Setup

A summary of the used components is provided at the end of this section. The microscope was built around a vertical bread board and a piezo/linear stage assembly (the white box in **Supplemental Figure S6A**) mounted on an air damped optical table (1200 by 1800 by 300 mm, 784 Performance Series, Technical Manufacturing Corporation). The piezo/linear stage assembly forms the first module of the interference cavity and holds the sample and both objective lenses and allows the sample position, interference cavity path length, and objective alignment to be adjusted remotely via computer control independent of the detection beam path (**Supplemental Figure S6**, **Supplemental Movie S7**). The vertical breadboard holds the second module of the interference cavity, deformable mirrors, and detection optics on its front surface and the excitation beam path on the back. Three excitation laser lines at wavelengths of 642 nm (MPB Communications, 2W), initially 561 nm (Coherent Genesis MX 56, 500 mW), later 560 nm (MPB communications, 2W) and 405 nm (Coherent OBIS 405 LX, 50 mW) reside on the optical table and are coupled into a polarization-maintaining single-mode fiber after passing through an acousto-optical tunable filter for wavelength selection and power modulation. The fiber delivers the excitation light to the back surface of the vertical breadboard where one of two optical paths may be selected via a pair of computer-controlled motorized mirrors (**Supplemental Figure S6C**). The first optical path illuminates an ~18 by 18 µm square area in the sample plane with nearly uniform illumination over the excitation field via an over-illuminated adjustable rectangular aperture. The fiber tip is conjugated to the objective back focal plane of the top objective lens and can be translated sideways for switching between epi-illumination and highly inclined and laminated optical sheet (HILO) imaging modalities. The second excitation path illuminates a ~100-µm diameter area for overview imaging and sample positioning.

Imaging is performed with two opposing high-NA oil immersion objective lenses (Olympus UPLSAPO 100XO PSF 1.4NA). The objective residing below the sample is mounted on a two-axis piezo stage (**Supplemental Figure S6B, blue stage**) (Physik Instrumente, P-612 2SL) which allows for lateral objective alignment with 5 nm resolution. The axial position of the upper objective is controlled with a nanopositioning stage (**Supplemental Figure S6B, green stage**) (Physik Instrumente, N-664.3A) with 0.5-nm resolution over a 16-mm travel range. Thus, the upper objective may be axially translated away from the lower objective for sample loading and then returned to the co-focal position with nanometer-accuracy. Additionally, a 940-nm diagnostic laser line is passed through the objective pair to facilitate real-time monitoring and correction of the axial and lateral objective alignment.

The axial position of the sample is controlled by a piezo stage (**Supplemental Figure S6B, magenta stage**) (Physik Instrumente, P-541.Z) with 0.5-nm resolution for precise and repeatable imaging of optical sections at various depths. This stage is, in turn, supported by a piezo-driven XY translation stage (**Supplemental Figure S6B, gray stage**) (Physik Instrumente, M-686.D64) with 100-nm resolution for coarse lateral sample positioning. The axial and XY sample stages are resting on a custom plate supported at three points of contact by three DC-Mike linear actuators (**Supplemental Figure S6B, gray columns**) (Physik Instrumente, N-227.10) with 100-nm repeatability for coarse axial positioning and tip/tilt sample adjustment. Two linear stages (**Supplemental Figure S6B, orange stages**) (ASI, LS-50) translate the entire objective-sample stage stack described above along the optical axis with a resolution of 10 nm allowing the optical path lengths of the two interference arms to be adjusted and maintained via computer control without affecting alignment in other areas of the system. **Supplemental Movie S7** shows an animation of the entire objective-sample stage stack assembly.

The back pupil planes of the upper and lower objectives are imaged onto two respective deformable mirrors (Boston Micromachines, Multi-5.5). 0.94x telescopes reduce the size of the objective pupils to match the active areas of the deformable mirrors. The deformable mirrors allow independent aberration compensation through both detection arms and facilitate aberration-free W-4PiSMSN imaging by correcting system and sample-induced aberrations.

A custom-made Babinet–Soleil compensator (UVisIR, custom BK7 and quartz blocks and wedges) allows system-level dispersion compensation and independent adjustment of the relative phase between the s- and p-polarizations in the two arms of the interference cavity (Aquino et al., 2011). The upper arm of the interference cavity includes a BK7 window bonded to a quartz wedge. A second quartz wedge is mounted adjacent to the first one on a motorized linear translation stage, allowing for relative phase adjustment between the two polarizations in the two cavity arms. The lower cavity arm includes a complementary quartz window bonded to a BK7 wedge. A matching BK7 wedge is also mounted on a motorized stage for dispersion compensation across the visible spectrum. Both cavity arms include electronically controlled shutters to allow recording PSFs through a single objective (either top or bottom) for independent aberration measurement and compensation. The final element in the interference cavity is a 50/50 beam splitter cube which is mounted on a motorized goniometer and rotation stage allowing the cube's reflective surface to be tipped and tilted without translation. This is critical for achieving uniform interference across the entire field of view and allows the beam splitter (BS) cube to be adjusted without perturbing alignment in other areas. In an arrangement similar to Aquino et al. (2011), relay optics direct the fluorescence exiting the 50/50 beam splitter cube along two separate paths to a single camera, in our case an sCMOS camera (Hamamatsu, ORCA-Flash 4.0v2) capable of recording 800 frames per second at 2048 x 256 pixels. This design allows us to project the four images (with $\sim\pi/2$ phase delays between the images) along the center splitting line of the upper and lower sCMOS rolling readout regions and therefore allows uncompromised camera frame rates. A motorized filter wheel (84889, Edmund Optics) was mounted in front of the camera to allow sequential two-color imaging.

In addition, a motorized flip mirror and an overview camera (PCO, pco.pixelfly usb) provide ~100 µm diameter field of view overview images of the sample, which help in finding and positioning areas of interest in the sample. Table below lists vendors and part numbers of the major components.

Part	Vendor	Part number/names
561 nm Laser	MPB Communications	2RU-VFL-P-2000-560-B1R
560 nm Laser	Coherent	Genesis MX 56 500 mW
642 nm Laser	MPB Communications	2RU-VFL-P-2000-642-B1R
405 nm Laser	Coherent	OBIS 405nm LX 50mW Laser
Deformable Mirrors	Boston Micromachines	Multi-5.5
Quartz and BK7 window	UVISIR	Custom made (call #: W-4PiSMSN)
AOTF	AA OPTO-ELECTRONIC	AOTFnC-400.650-TN
Detection filters	Semrock	FF01-607/70-25
Detection filters	Chroma	ET700/75m
Piezo stages	PI	M-686.D64 XY Stage

Piezo stages	PI	P-541.ZCD Z Stage
Piezo stages	PI	P-612.2SL XY Stage
Piezo stages	PI	N-664.3A Linear Stage
Linear Actuator	PI	M-227.10
Vertical Translational Stage	ASI	LS-50 (FTP mode)
Camera	Hamamatsu	ORCA-Flash4.0 V2 Digital CMOS Camera 22CU
Quad bandpass filter	Semrock	FF01-446/523/600/677-25
Dichroic mirrors	Semrock	405/488/561/635 BrightLine Laser Dichroic
Objectives	Olympus	UPLSAPO 100XO
Analysis software	MathWorks	MATLAB
GPU	Nvidia	GeForce GTX 580
Visualization software	Multiple	Vutara (Bruker), Avizo (FEI Software), Maya 2015 (Autodesk)

2. Characterization of Deformable Mirrors

Deformation of the reflective membrane on the BMC Multi-5.5 deformable mirror (DM) is induced by a set of 140 actuators positioned in a grid-patterned array beneath the membrane. Each actuator adds a degree of freedom to the shape of the deformable mirror. As previously shown (Wang and Booth, 2009), the possible mirror shapes can be decomposed into an orthogonal set of so-called mirror deformation eigenmodes (in short, **mirror modes**), resembling Zernike modes while accounting for the stiffness of the membrane and the spatial distribution of the actuators. One mirror mode is essentially a set of displacements applied to the actuator array. To establish the actual shape of the mirror when applying a given mirror mode, a DM characterization process was followed as detailed previously (Burke et al., 2015). This process relies on a phase-retrieval scheme (Hanser et al., 2004) (using the Gerchberg-Saxton algorithm) and takes as input a set of images of a point emitter at known axial positions near the objective focal plane. We used a sub-diffraction sized fluorescent bead (100 nm crimson, Life Technologies) and imaged it at five known axial positions: z = -1 µm, -0.5 µm, 0 µm, 0.5 µm and 1 µm, where z = 0 µm corresponds to the objective focal plane. Given one such three-dimensional image set, the phase retrieval scheme (Hanser et al., 2004) provides an estimate of the pupil function, i.e. the wave-front shape at the objective pupil plane.

For each mirror mode, we retrieved pupil functions for five different mirror mode amplitudes. Subsequently, the pupil functions were each decomposed into a set of Zernike modes. By fitting a first-order polynomial through the Zernike mode coefficients as a function of mirror mode amplitudes, we determined the first 55 Zernike mode coefficients for each of the applied 28 mirror modes. The resulting coefficients were used to form an underdetermined system of 28 linear equations each describing the Zernike mode constituents of a single mirror mode. By solving this system of linear equations in the least square sense, each Zernike mode can be expressed as a linear combination (weighted sum) of mirror modes. This calibration process was carried out separately for the top and bottom interference arms.

3. System Aberration Correction

System aberrations for the upper and lower beam paths were corrected separately. The corresponding deformable mirrors were independently adjusted as follows. For each interference arm, starting from the flat voltage map (provided by the manufacturer) of the deformable mirror, 28 mirror modes (Wang and Booth, 2009) were applied sequentially. For each mirror mode, 10 different amplitudes were applied while recording the corresponding fluorescence signal from a 100-nm crimson bead sample. To extract the fluorescence signal from individual beads, the symmetry center of each imaged bead was obtained using the radial symmetry method (Parthasarathy, 2012). Subsequently, a symmetric 2D Gaussian was generated at the symmetry center and was multiplied by the isolated emission pattern from the fluorescent bead, generating a Gaussian-masked image, and then the total intensity of the masked image was calculated to extract the center peak signal of the beads in focus. For each mirror mode, images of the bead were acquired at ten different mirror mode amplitudes and the corresponding center peak signals of the bead were extracted as described above. The optimal amplitude (i.e. the amplitude providing the highest center peak signal from the beads) was determined from a quadratic fit of these ten signal measurements vs. mirror mode amplitudes. After identifying optimal amplitudes for each of the 28 modes, these amplitudes were added to the flat voltage map (provided by the manufacturer), serving as a starting point for another iteration. This iterative process was repeated five times to achieve optimal system aberration correction.

4. Channel Registration

Each sCMOS camera frame contains four images (arranged next to each other) that represent the same field of view of the sample at different interference phase delays (Aquino et al., 2011). In the following, we refer to these images as ***phase images***. One of the phase images was taken as the reference, and all the other three phase images were merged (added) into the reference image using three affine transformations (one for each phase image) where the transformations were obtained from a calibration bead data set taken prior or after the imaging session (200 frames of approximately 10-15 beads in focus imaged with a single objective). To ensure accurate merging of the four phase images, our estimation of each transformation matrix (including magnification, translation and rotation) followed the following steps: first, a Fourier-Mellin transform (implemented as "fmmatch" using the MATLAB dipimage toolbox, www.diplib.org) was used to obtain an initial estimation of the transform. Second, the affine transform was then obtained using the previous result from Fourier-Mellin transform as an initial guess (implemented as "find_affine_trans" using the dipimage toolbox). These two sequential steps took advantage of the fact that the log-polar transformation is invariant with translation in the image and the least-square approach to find affine transformations works well when the relative shifts between two images are small.

5. Lateral Position Localization

To estimate the sCMOS camera characteristics (including readout noise, offset and gain for each pixel) in the combined frame described in the section above, noise statistics maps from all four phase images were merged in the same way as the phase images and subsequently used as the noise map in the subsequent fitting process (Huang et al., 2013).

As described previously (Huang et al., 2013), a series of uniform and maximum filters were used to find isolated single molecules and sub-regions were cropped around these fitting candidates. The isolated sub-regions were then fit using the Maximum Likelihood Estimator (MLE) with the sCMOS noise model (Huang et al., 2013) to an elliptical 2D Gaussian (Huang et al., 2008) and estimates of single-molecule positions (x, y), standard deviations (σ_x, σ_y), total number of detected photons, background photon counts and log-likelihood ratio values were obtained. The threshold of the log-likelihood ratio metric (Huang et

al., 2011) (used as the goodness of fit test) was set to a relatively large value with the intention to filter out sub-regions containing multiple emitters.

6. Extraction of Single-molecule Phase

To estimate the phase of the single-molecule interference (a prerequisite to determine the z-position of the molecule), the estimated lateral single-molecule positions (x, y) were inverse-transformed (affine) back to the four separate phase images. Each of these positions pinpoints the center location of the single molecule in the four phase images in W-4PiSMSN. Using these center locations of single molecules in the four phase images, the 0th moment intensities (Aquino et al., 2011) were calculated by a weighted least-square fit of a Gaussian. As the center location of the molecule is already known, the weighted least-square fit was used to estimate the amplitude. Due to the pixel-dependent readout noise of sCMOS cameras, the weight for each pixel i is assigned as $D_i + \frac{\sigma_i^2}{g_i^2}$, where D_i is the pixel count, and σ_i^2 and g_i are the pixel-dependent readout noise variance and gain of the sCMOS sensor, respectively. In this way, we take the sCMOS-specific pixel-dependent noise into account (Huang et al., 2013).

The phase shift between s and p-polarization (Aquino et al., 2011) was adjusted such that it is close to π/2 for both detection channels. However, due to dispersion, this phase shift differed by ~0.3 radians in our system for our two imaging channels (607 nm and 700 nm center wavelengths). We calibrated the phase shifts for the two color channels independently using a bead sample which could be observed in both color channels (100 nm crimson beads).

To extract the interference phase of each single molecule, similar to the method previously described by Aquino et al., 2011, we extracted the 0th moment amplitudes of each single molecule from the four phase images and subsequently the reduced moments (Aquino et al., 2011), RM_s and RM_p, were calculated. Using the previously calibrated phase shifts, we obtained the phase of the single-molecule interference PSF by solving the set of equations (1).

$$\begin{cases} A_0 \cos(\varphi_0) - RM_s = 0 \\ A_0 \cos(\varphi_0 + \varphi_{shift\_ch}) - RM_p = 0 \end{cases} \qquad \text{Eq. 1}$$

A_0 and φ_0 are the unknowns representing the amplitude and single molecule phase, respectively. φ_{shift_ch} was previously obtained through the calibration using the bead sample for a specific channel and RM_s and RM_p are the reduced moments of 0th order (Aquino et al., 2011). For two-color imaging, the values of φ_{shift_ch} were different for the two different wavelength channels and were obtained for each color channel through the calibration.

7. Axial Localization Based on Monotonic Metric and Local Ridge Detection

Accurate and precise axial position estimation can be challenging when based only on astigmatism because even a small amount of sample-induced aberrations creates image distortions and artifacts which cause the PSF to deviate from the calibration curve (Liu et al., 2013; McGorty et al., 2014). This effect is increasingly problematic when imaging deep into a sample. Single-molecule interference, however, only relies on the path-length differences between the two interference arms and thus provides the opportunity for accurate large volume super-resolution imaging. Due to these concerns, we did not use the astigmatic shape information for our axial localization, but solely to unwrap the single-molecule phase and thus avoid distortions and artifacts as described below.

We started with a segment of W-4PiSMSN data (usually 3,000-5,000 frames, 10-200 segments per dataset) containing single-molecule emissions events. For each single-molecule emission, the localization methods described above allowed us to extract the interference phase φ_0 and standard deviations of the

2D Gaussian σ_x and σ_y. Thus, we obtained a list of these values for all single-molecule detection events in the data segment. Next, we introduced a metric, $m = \frac{{\sigma_x}^3}{\sigma_y} - \frac{{\sigma_y}^3}{\sigma_x}$, which describes the overall shape of the emission events and preserves its monotonicity in the presence of a small amount of aberrations. For numerical convenience, we normalized $\boldsymbol{m}$ by 2π/40. From this list of $\{m\}$ and $\{\varphi_0\}$ values for all single-molecule detection events in the data segment, we generated a 2D histogram image. **Supplemental Figure S3 A** shows a contour plot of such a histogram. As m is monotonic against the axial position and φ_0 is periodic with a period of 2π, the resulting 2D histogram resembles tilting stripes which are repeated over the range of the (normalized) metric m (**Supplemental Figure S3 A**).

Using the histogram, the problem is now reduced to a phase unwrapping problem. We seek to find a continuous phase variation, φ_0, with respect to m in an ensemble collection of single-molecule detection events**.** To do this, we developed a ridge finding algorithm that incrementally follows the peak of these stripes with the following three properties: first, it self-adapts to the shape and curvature of the stripes (**Supplemental Figure S3 B**). These shapes and curvatures are sample and depth-dependent and also vary between data segments within a single dataset. Second, the algorithm finds a continuous ridge through the histogram without jumping to the adjacent ridge (**Supplemental Figure S3 B**). This is important because errors in this aspect cause errors in the unwrapping step that lead to localization artifacts and ghost images in the final image (**see paragraph below**). Third, the algorithm is monotonic in the sense that no two points on the growing path share the same value of m to ensure unambiguous position assignment of single molecules.

To find the ridge along the wrapping histogram stripes, we started with an initial peak-finding process: the maximum peak spot was first identified (P_0) and the second peak (P_1) was then identified by searching within a radius range defined by r_{min} and r_{max} (**Supplemental Figure S3A**) from P_0. The search range restriction was used to allow the generation of an initial vector ($\overrightarrow{P_0P_1}$) that points along the direction of the running ridge (**Supplemental Figure S3A**). Next, we searched for the next peak starting from P_1. We assigned the search radius range as r_{min} and r_{max} centering around P_1 and also assigned the direction (or vision) of the search (**Supplemental Figure S3A**) in the direction of $\overrightarrow{P_0P_1}$ with ~0.2 rad angle width (adjustable parameter) forming a cone shaped "search vision". The next peak, P_2, was identified by finding the pixel with maximum value on the 2D histogram within the search range and search vision centered around P_1. Subsequently, P_2 served as the starting point for the next search, and so on. In this way, the path grew up to P_n where the value of the 2D histogram at the next peak P_{n+1} was less than a threshold. Therefore, the path $P_0P_1 \ldots P_n$ identified half of the ridge of the histogram. Next, we used the same approach to find the path in the opposite direction (starting with P_0 with a initial direction of ($\overrightarrow{P_1P_0}$)) and generated the other half of the path $P_1P_0 \ldots P_{-k}$. The two paths (forward and backward) were subsequently combined ($P_{-k} \ldots P_0P_1 \ldots P_n$) providing a piece-wise monotonic, self-adapting and unambiguous curve through the phase-metric plot.

With the piece-wise monotonic path, we can now un-wrap it (Judge and Bryanston-Cross, 1994) into a monotonic path (implemented using "unwrap" in MATLAB). For each single-molecule detection event, with a corresponding m and φ_0 pair, we determined the point P_{path} on the piece-wise monotonic path that gives the minimum distance to point (m, φ_0). Subsequently, we unwrapped the (m, φ_0) together with P_{path} by adding the same integer multiplication of 2π to (m, φ_0) as it was added during the unwrapping process for P_{path}. Therefore, with the help of our shape metric m, this process unwrapped $\{\varphi_0\}$ and resolved the ambiguity due to its periodicity. To allow accurate translation of phase-unwrapped values $\{\varphi_0\}$ to axial position estimates $\{z_{est}\}$, we simulated W-4PiSMSN PSFs using a pupil function-based approach (Hanser et al., 2004). W-4PiSMSN PSFs were simulated for both detection channels (607 nm and 700 nm). Modulation frequencies (unit: radians (φ_0, phase) per nm (z_{est}, axial position)) were

identified in these PSFs and were used to translate the phase into axial positions in both single-color and two-color measurements.

8. 3D Drift Correction with Redundancy

Based on the idea of the previously published redundancy-based drift correction method in 2D (Li et al., 2013; Wang et al., 2014), we developed a 3D drift correction. First, similar to other drift correction algorithms (Mlodzianoski et al., 2011), the entire dataset was split into n segments (usually 3,000-5,000 frames each, resulting in n = 10 to 200 segments per dataset). For each data segment, a volume image with (25 nm)^3 voxel size was reconstructed as a 3D histogram where the count for each pixel in the histogram equals the number of localization estimates falling into the voxel. 3D cross-correlation was then used to calculate the shift distance between each pair of data segment volumes. To find the correlation peak from the 3D cross-correlation image, Fourier interpolation was used to identify the peak with an effective pixel size of 1.2 nm in the final interpolated image. This process pinpoints the shift distance $(x_{shift}^{i\to j}, y_{shift}^{i\to j}, z_{shift}^{i\to j})$ between two data segment volumes (i and j). For a total of n data segments, there are $\frac{1}{2}n(n+1)$ of such shift measurements (which are not independent) forming an overdetermined system to determine independent shifts between adjacent data segments, $\{(x_{shift}^{0\to 1}, y_{shift}^{0\to 1}, z_{shift}^{0\to 1}), \dots, (x_{shift}^{i\to i+1}, y_{shift}^{i\to i+1}, z_{shift}^{i\to i+1}), \dots, (x_{shift}^{n-1\to n}, y_{shift}^{n-1\to n}, z_{shift}^{n-1\to n})\}$, including a total of $n-1$ unknown independent shifts. For each coordinate (x, y and z), a system of linear equations was established as described in Li et al., 2013. Least-square solutions that minimize the overall error of the equation stacks were calculated and substituted back into all equations. Residual errors can be calculated for each of these equations and an equation is removed from the system of linear equations if its error is larger than 7 nm. This process was repeated until no single equation gave an error larger than 7 nm or the system of linear equations was no-longer at its full rank.

9. Data Analysis for Multi-optical Section Data

To image thick samples, optical sections were recorded at different axial positions of the sample by axially translating the z-piezo holding the sample stage. The localization data contains x, y, and z position estimates of different optical sections and must be aligned/stitched seamlessly to support the high precision obtained in W-4PiSMSN. Previous methods (Huang et al., 2008) that shift each optical section by a constant in the axial direction have been prone to introduce misalignment of the optical sections and subsequently deteriorate the resolution achievable in thick samples. Here, we developed an optical alignment method based on 3D cross-correlation. In the W-4PiSMSN system, optical sections are ~1.2 μm thick. Whole-cell samples were scanned in the axial direction with 500-nm step sizes which allowed for abundant overlapping regions between adjacent optical sections. This overlapping information between optical sections is critical for precise optical section alignment using the cross-correlation methods described below.

Similar to **Supplemental Information 8**, for each data segment (an optical section in this case), a volume image with (25 nm)^3 voxel size was reconstructed as a 3D histogram where the count for each pixel in the histogram equals the number of localization estimates within the voxel. Given a 500-nm axial step size of the sample stage, we observed an effective shift of only ~400 nm between adjacent reconstructed optical sections. This inconsistency is explained by the index of refraction mismatch between the sample (~1.33-1.37) and the immersion media (~1.51). Therefore, expecting a peak around 400 nm in our correlation volume (generated as described in **Supplemental Information 8**), we isolated a small 3D sub-volume around this expected center and determined the local peak within this sub-volume. In this way, we isolated the desired peak from the noise-induced peaks that are usually located in the center of the correlation volume. The noise induced center peak is especially strong and thus problematic when two images (in our case volumes) only partially overlap, which is here the case. As described above, to find

the local correlation peak from the 3D cross-correlation sub volume, Fourier interpolation was used to identify the peak with an effective pixel size of 1.2 nm in the final interpolated image (Li et al., 2013). The resulting peak identifies shifts between the two adjacent optical sections.

10. Multi-color Data Alignment

Two-color imaging was performed sequentially, first in the Alexa Fluor 647 (AL647) channel and then the Cy3B channel. Specifically for two-color imaging, we shifted all the AL647 data segment volumes in the drift correction step (**Supplemental Information 8**) to align with the *last* data volume at the end of the AL647 imaging session. For the Cy3B channel, we aligned all Cy3B data segment volumes with the *first* data segment volume in the beginning of the Cy3B imaging session. There was a 2-4 s pause between the recording of the two color channels and we assumed system drift and sample drift during this interval to be negligible.

To align 3D volumes after drift correction from one color channel to another, we obtained a 3D affine transformation from a training dataset using double-stained (AL647 and Cy3B) mitochondria (implemented as "imregtform" in MATLAB) from 3D histogram image as described in **Supplemental Information 8** and **9** with a voxel size of $(25\ \text{nm})^3$. The obtained registration matrix (affine) was then applied to all single-molecule position estimates from one channel to allow 3D color registration of two color volumes.

11. Cilia Membrane Flattening

First, a manually selected region of interest (ROI) is fit (least square) with a cylinder model. Then the region was aligned such that the cylinder axis became the z-axis and the ROI was centered at the origin of the coordinate system. Based on the new coordinate system, each single-molecule localization was transformed into a modified version of cylindrical coordinates (ρ, φ, z). Surface plots were obtained by generating a 2D histogram of the cylindrical coordinates of single molecules as $\rho - r$ and φ, where r is the radius of the fitted cylinder model.

12. Template-Free Point Cloud Registration for T7 Phage Data

A total of 115 T7 phages, each represented by a point cloud, were automatically identified from 14 W-4PiSMSN datasets. The orientation and the structural center were obtained by fitting the point cloud to a straight line and sphere, respectively. All phages were then centered at the origin of a Cartesian coordinate system and rotated such that their orientation (icosahedron capsid) aligned with the z-axis. These pre-aligned phages were then aligned to each other using Gaussian kernel correlation registration (Tsin and Kanade, 2004), with a simplified version of the pyramid scheme described previously (Broeken et al., 2015), by allowing it to rotate around the z-axis. This second alignment step created an initial guess for the final phage averaged structure. The final averaged structure was obtained by aligning all original phage results to the initial guess. To improve performance of the registration algorithm, a fast Gauss transform (Jian and Vemuri, 2011) was implemented.

13. Point Cloud Clustering Algorithm to Isolate Synaptonemal Complexes

Paired strands of synaptonemal complexes (**Figure 7**) were isolated using a clustering algorithm (Klasing et al., 2008) where single-molecule localization estimates located within a 500-nm distance from each other were assigned to the same cluster. The algorithm terminated when all points were processed and assigned to clusters. To avoid over-counting caused by unspecific labeling, final clusters with fewer than 750 points were discarded.

14. Noise Reduction

To allow robust and precise feature detection and alignment in point cloud data, the point clouds were first processed through a noise filter where all points whose number of neighbor points within a given distance was below a user-defined threshold were discarded. While this noise filter was only used to improve feature detection and model construction, the raw single-molecule localization results (without de-noising) were used after the alignment process to provide quantifiable final results. This method was applied to help feature extraction in phage, cilia and synaptonemal complex datasets and to generate the shown phage averaged reconstruction (**Figure 1I-L**) and images of isolated strands of synaptonemal complex (**Figure 7 E and F**).

15. Coverslip Preparation and Cell Culture

25 mm diameter round precision glass cover slips (Bioscience Tools, San Diego, CA) were immersed in 1M KOH and sonicated for 15 min in an ultrasonic cleaner (2510 Branson, Richmond, VA). The glass was then generously rinsed with Milli-Q water (EMD Millipore, Billerica, MA) and sterilized with 70% ethanol. The glass was dried and poly-L-lysine coated before 100-nm Crimson beads (Life Technologies, Grand Island, NY) were attached to the top surface. Before cells were plated on the beads, the surface was rinsed three times with Phosphate-buffered saline (**PBS**). Cells were grown on coverslips for 2-24 hours before fixation.

COS-7 cells (ATCC, CRL-1651) were grown in DMEM (Gibco, 21063-045) with 10% Fetal Bovine Serum (FBS) and 1% Penicillin-Streptomycin (Gibco, 15140-122) at 37 °C with 5% CO_2. BSC1 cells (ATCC, CCL-26) were grown in DMEM (Gibco, 21063-045) with 10% FBS at 37 °C and 5% CO_2. RPE-hTERT cells were grown in DMEM/F12 (Gibco 11330-032) with 10% FBS and 1% Antibiotic-Antimycotic (Gibco, 15240-062) at 37 °C and 5% CO_2.

16. Secondary Antibody Labeling

Except where noted otherwise, primary antibodies were labeled with Alexa Fluor 647-conjugated goat anti-mouse or goat anti-rabbit secondary antibodies (Thermo Fisher Scientific, A21236, A21245, Waltham, MA). Secondary antibodies labeled with Cy3B were made by reacting Cy3B NHS Esters (GE Healthcare, Malborough, MA) with unlabeled secondary antibodies (Jackson ImmunoResearch Laboratories, Inc., West Grove, PA) according to the manufacturer's protocol. Free dye was separated from labeled antibody by gel filtration using an illustra NAP-5 column (GE Healthcare). Samples were labeled with secondary antibodies at a dilution between 1:1000 and 1:200 for 30 to 60 min at room temperature. Where noted, a post-fixation step of 3% paraformaldehyde (PFA, Electron Microscopy Sciences, 15710, Hatfield, PA) + 0.1% glutaraldehyde (GA, Electron Microscopy Sciences, 16019, Hatfield, PA) was performed after secondary antibody labeling. Samples were rinsed three times with PBS and stored in PBS until they were imaged.

17. Endoplasmic Reticulum Samples

COS-7 cells were grown on prepared coverslips and then transfected with *mEmerald-Sec61-C-18*, a gift from Michael Davidson (Addgene plasmid # 54249), using Lipofectamine2000 (Thermo Fisher Scientific). 12-24 h later, cells were fixed using 3% PFA + 0.1% GA in PBS for 15 min. Cells were permeabilized for 3 min at room temperature with 0.3% IGEPAL-630 (Sigma-Aldrich) + 0.05% Triton X-100 (Sigma-Aldrich) + 0.1% BSA in PBS. Samples were blocked with blocking buffer (5% normal Goat serum, 0.05% IGEPAL-630, 0.05% Triton X-100 in PBS). Rabbit anti-GFP (Thermo Fisher Scientific, A-11122) was used at 1:500 to label mEmerald-Sec61β overnight at 4 °C. Antibodies were diluted in blocking buffer. Samples were washed in wash buffer (WB, 0.2% BSA, 0.05% IGEPAL-630, 0.05% Triton X-100 in PBS) for 5 min three times before labeling with secondary antibody for 1 h at

room temperature. Samples were then washed again in WB for 5-min incubations three times before they were post-fixed with 3%PFA+0.1%GA.

18. Microtubule Samples

Microtubule samples were prepared similar to our previous report (Huang et al. 2013). COS-7 cells were grown on prepared coverslips. Cells were rinsed three times with 37 °C PBS before a 1-min pre-extraction incubation in pre-warmed 0.2% saponin in cytoskeleton buffer (CBS, 10 mM MES pH 6.1, 138 mM NaCl, 3 mM $MgCl_2$, 2 mM EGTA, 320 mM sucrose) to remove tubulin monomers from the cell cytoplasm. Immediately following the pre-extraction step, the cells were fixed for 15 min at room temperature in 3% PFA and 0.1% GA diluted in CBS. After fixation, the cells were rinsed three times in PBS before being permeabilized and blocked in blocking buffer (3% bovine serum albumin (BSA, Jackson ImmunoResearch) and 0.2% Triton X-100 in PBS) for 30 min at room temperature. Mouse anti-α-tubulin antibody (Sigma-Aldrich, T5168, St. Louis, MO) was used at 1:1000 dilution for a 4 °C overnight incubation. Antibodies were diluted in 1% BSA and 0.2% Triton X-100 in PBS. Cells were washed three times for 5 min each in wash buffer (WB, 0.05% Triton X-100 in PBS). Secondary antibodies were used to label cells for 1 h at room temperature. Cells were washed again in WB for 5-min incubations three times and then post-fixed with 3% PFA + 0.1% GA for 10 min. Samples were rinsed three times with PBS.

19. T7 Bacteriophage Samples

T7 bacteriophage lysate was prepared from 100 ml of *E. coli* MG1655 cultures grown in liquid broth (LB) at 30 °C. The phage lysate was subjected to PEG precipitation and cesium chloride-gradient centrifugation as described in Chan et al., 2005 with some modifications. Following the PEG precipitation step, ~300 µl of phage in borate buffer (50 mM borate, pH 8.5) was incubated with 100 µg of Alexa Fluor 647 NHS Ester (4 µg/µl in DMSO; Thermo Fisher Scientific) for 30 min at room temperature. The labeled phage particles were first purified using a Bio-Spin P30 column (Bio-Rad Laboratories, Hercules, CA) to remove most of the free dye and then subjected to cesium chloride gradient centrifugation. Cesium chloride was removed using a Bio-Spin P30 column and phage particles were eluted in T7 storage buffer (10 mM Tris pH 7.5, 10 mM $MgCl_2$).

Coverslips were cleaned as described in (Lim et al., 2014). Briefly, the coverslips were cleaned by sonication in 1M KOH, double-distilled H_2O, and 70% ethanol for 15 min each at room temperature. 0.1% poly-L-lysine was added to the cleaned coverslip, incubated for 30 min at room temperature, washed with T7 storage buffer, and then dried with pressured air. For imaging, 20 µl of the phage lysate was spotted onto cleaned coverslip, incubated for 1 min at room temperature, and then washed extensively with T7 storage buffer. The coverslip was air-dried before imaging.

20. Mitochondria Samples and Two-color Mitochondria & Microtubule Samples

COS-7 cells were grown on prepared coverslips and fixed using 3% PFA + 0.1% GA in PBS for 15 min. Cells were permeabilized for 3 min at room temperature with 0.3% IGEPAL-630 + 0.05% Triton X-100 + 0.1% BSA in PBS. Samples were blocked with blocking buffer (5% normal Goat serum, 0.05% IGEPAL-630, 0.05% Triton X-100 in PBS). Rabbit anti-TOM20 (Santa Cruz Biotechnology sc-11415, Dallas, TX) was used at 1:500 and mouse anti-α-tubulin (Sigma-Aldrich, T5168) was used at 1:1000 and they were incubated with samples overnight at 4 °C. Antibodies were diluted in blocking buffer. Cells were washed in wash buffer (WB, 0.2% BSA, 0.05% IGEPAL-630, 0.05% Triton X-100 in PBS) for 5-min incubations three times. Cells were labeled with secondary antibodies for 1 h at room temperature. Then samples were washed again with WB for 5 min three times before they were post-fixed with 3% PFA + 0.1% GA.

21. Nuclear Pore Complexes Samples

hTERT-RPE1 cells were grown to 100 percent confluence on prepared coverslips. Cells were pre-permeabilized with pre-warmed 0.1% Saponin in PBS for 1 min. Cells were then rinsed with pre-warmed PBS and fixed with methanol at -20 °C for 5 min. Samples were blocked with 5% BSA + 0.1% Triton X-100 in PBS. After blocking, cells were labeled with goat anti-rabbit Nup358 for 1 h at room temperature. Antibodies were diluted in 1% BSA + 0.1% Triton X-100 in PBS. Cells were washed three times with PBS for 10 min each. Primary antibodies were labeled with secondary antibodies for 1 h at room temperature. Cells were washed three times in PBS for 10 min each. After washing, cells were post-fixed with 2% PFA in PBS for 2 minutes.

22. COPI Samples

BSC1 cells were grown on prepared coverslips and fixed with 4% paraformaldehyde in PBS for 15 min. Cells were permeabilized for 3 min at room temperature with 0.3% IGEPAL-630 + 0.05% Triton X-100 + 0.1% BSA in PBS. Samples were blocked in blocking buffer (5% normal Goat serum, 0.05% IGEPAL-630, 0.05% Triton X-100 in PBS). Mouse anti- ’ COP (Palmer et al., 1993) was used to label COPI at 1:2000 dilution in blocking buffer overnight at 4 °C. Cells were washed in wash buffer (WB, 0.2% BSA, 0.05% IGEPAL-630, 0.05% Triton X-100 in PBS) three times for 5 min each before labeling with secondary antibody for 1 h at room temperature. Samples were then washed with WB for 5 min three times before being post-fixed with 3% PFA + 0.1% GA.

23. Cilia Samples

hTERT-RPE1 cells that stably express pHlourin-Smoothened (pH-SMO) were grown on prepared coverslips. To induce ciliogenesis, the cells were incubated in DMEM/F12 media with 0.5% FBS and 100 nm Cytochalasin D for 48 h.

After ciliogenesis induction, cells were washed twice in PBS and fixed for 10 min with 4% PFA + 0.2% GA + 0.1% Triton X-100 in PBS. Fixed cells were then washed twice with PBS + 0.05% Tween20 followed by a 30 min incubation in blocking buffer (5% BSA + 0.05% Tween20 in PBS). Cells were incubated with the primary rabbit anti-GFP antibody (Thermo Fisher Scientific, A11122) at 1:500 dilution in blocking buffer for 1 h at room temperature. Then cells were washed with three 5 min incubations in wash buffer (0.05% Tween20 in PBS). Cells were labeled with secondary antibody diluted in blocking buffer for 30 min at room temperature. Samples were washed three times for 5 min each in wash buffer, and followed by two rinses in PBS.

24. Synaptonemal Complex Samples

Testes were removed from 17-18 day old euthanized mice. The protocols for the care and use of mice at suitable ages were approved by the Institutional Animal Care and Use Committee (IACUC) of The Jackson Laboratory. Each testis was disrupted in PBS supplemented with protease inhibitors using a razor blade. The cell pellet was collected after centrifugation at 9,000 rpm for 10 min. The cells were resuspended and allowed to settle on prepared coverslips. They were then fixed with 4% PFA for 15 min, rinsed with PBS three times, and permeabilized with 0.5% Triton X-100 in PBS for 10 min. Before incubating with primary antibody, spermatocytes were treated with Image-iT signal Enhancer (Thermo Fisher Scientific) and blocked with MAXblock (Active Motif). Cells were stained with anti-SYCP3 (Abcam, ab15093, Cambridge, MA) overnight at 4 °C. Cells were then washed three times for 5-min incubations in wash buffer (WB, 0.1% Triton X-100 in PBS) before labeling with secondary antibodies for 3 h at 37 °C. Cells were washed again three times for 5 min each in WB.

25. Imaging Buffers

Two different imaging buffers were used.

The conventional β-mercaptoethanol imaging buffer was prepared as previously reported (Huang et al 2013). The imaging buffer was made immediately before use where catalase and glucose oxidase were diluted in base buffer (50 mM Tris pH 8.0, 50 mM NaCl, 10% glucose).

The imaging buffer containing cyclooctatetraene (COT) was prepared according to a previously published report (Olivier et al., 2013). Mercaptoethylamine (MEA, Sigma-Aldrich, 30070) was dissolved in deionized water as 1M stock solution, and then adjusted to pH 8 by glacial acetic acid (Avantor Performance Materials). The stock solution was stored at 4 °C and used within a week. β-mercaptoethanol (BME, Sigma-Aldrich, 63689) was used without dilution as 14.3 M solution. Cyclooctatetraene (COT, Sigma-Aldrich, 138924) was diluted in DMSO as 200 mM stock solution and stored at 4 °C. Protocatechuic acid (PCA, Sigma-Aldrich, 37580) was dissolved in deionized water as 100 mM stock solution, then adjusted to pH 9 by KOH aq. The stock solution was stored at 4 °C and used within a month. Protocatechuate 3,4-dioxygenase from *Pseudomonas* sp. (PCD, Sigma-Aldrich, P8279) was dissolved in 100 mM Tris-HCl (pH 8) containing 50 mM KCl, 1 mM EDTA and 50% glycerol as 5 μM stock solution, and stored at −20 °C. The imaging buffer consists of base buffer (50 mM Tris pH 8.0, 50 mM NaCl, 10% glucose) with the addition of 10 mM MEA, 50 mM BME, 2 mM COT, 2.5 mM PCA and 50 nM PCD. The buffer was prepared immediately before use.

26. Sample-mounting in W-4PiSMSN

Prepared sample coverslips were drained and subsequently mounted on a custom-designed sample holder. A custom-made spacer ring (9513K111, McMaster-Carr, Princeton, NJ) was put on top of the sample coverslip and then 50 μL imaging buffer as described above, was added to the center of the coverslip. Another coverslip was put on top and excess imaging buffer was drained. The samples were then sealed with two-component silicone putty (Picodent Twinsil, Picodent, Wipperfürth, Germany). After solidification of the silicone, the samples were transferred to the W-4PiSMSN microscope for imaging.

Supplemental References

Aquino, D., Schönle, A., Geisler, C., Middendorff, C. V, Wurm, C.A., Okamura, Y., Lang, T., Hell, S.W., and Egner, A. (2011). Two-color nanoscopy of three-dimensional volumes by 4Pi detection of stochastically switched fluorophores. Nat. Methods *8*, 353–359.

Broeken, J., Johnson, H., Lidke, D.S., Liu, S., Nieuwenhuizen, R.P.J., Stallinga, S., Lidke, K.A., and Rieger, B. (2015). Resolution improvement by 3D particle averaging in localization microscopy. Methods Appl. Fluoresc. *3*, 014003.

Burke, D., Patton, B., Huang, F., Bewersdorf, J., and Booth, M.J. (2015). Adaptive optics correction of specimen-induced aberrations in single-molecule switching microscopy. Optica *2*, 177.

Chan, L.Y., Kosuri, S., and Endy, D. (2005). Refactoring bacteriophage T7. Mol. Syst. Biol. *1*, 2005.0018.

Hanser, B.M., Gustafsson, M.G.L., Agard, D.A., and Sedat, J.W. (2004). Phase-retrieved pupil functions in wide-field fluorescence microscopy. J. Microsc. *216*, 32–48.

Huang, B., Jones, S.A., Brandenburg, B., and Zhuang, X. (2008). Whole-cell 3D STORM reveals interactions between cellular structures with nanometer-scale resolution. Nat. Methods *5*, 1047–1052.

Huang, F., Schwartz, S.L., Byars, J.M., and Lidke, K.A. (2011). Simultaneous multiple-emitter fitting for single molecule super-resolution imaging. Biomed. Opt. Express *2*, 1377–1393.

Huang, F., Hartwich, T.M.P., Rivera-Molina, F.E., Lin, Y., Duim, W.C., Long, J.J., Uchil, P.D., Myers, J.R., Baird, M.A., Mothes, W., et al. (2013). Video-rate nanoscopy using sCMOS camera-specific single-molecule localization algorithms. Nat. Methods *10*, 653–658.

Jian, B., and Vemuri, B.C. (2011). Robust point set registration using Gaussian mixture models. IEEE Trans. Pattern Anal. Mach. Intell. *33*, 1633–1645.

Judge, T.R., and Bryanston-Cross, P.J. (1994). A review of phase unwrapping techniques in fringe analysis. Opt. Lasers Eng. *21*, 199–239.

Klasing, K., Wollherr, D., and Buss, M. (2008). A clustering method for efficient segmentation of 3D laser data. In 2008 IEEE International Conference on Robotics and Automation, (IEEE), pp. 4043–4048.

Li, X., Mooney, P., Zheng, S., Booth, C.R., Braunfeld, M.B., Gubbens, S., Agard, D.A., and Cheng, Y. (2013). Electron counting and beam-induced motion correction enable near-atomic-resolution single-particle cryo-EM. Nat. Methods *10*, 584–590.

Lim, H.C., Surovtsev, I. V., Beltran, B.G., Huang, F., Bewersdorf, J., and Jacobs-Wagner, C. (2014). Evidence for a DNA-relay mechanism in ParABS-mediated chromosome segregation. Elife *2014*.

Liu, S., Kromann, E.B., Krueger, W.D., Bewersdorf, J., and Lidke, K.A. (2013). Three dimensional single molecule localization using a phase retrieved pupil function. Opt Express *21*, 29462–29487.

McGorty, R., Schnitzbauer, J., Zhang, W., and Huang, B. (2014). Correction of depth-dependent aberrations in 3D single-molecule localization and super-resolution microscopy. Opt. Lett. *39*, 275–278.

Mlodzianoski, M.J., Schreiner, J.M., Callahan, S.P., Smolková, K., Dlasková, A., Šantorová, J., Ježek, P., and Bewersdorf, J. (2011). Sample drift correction in 3D fluorescence photoactivation localization microscopy. Opt. Express *19*, 15009.

Nieuwenhuizen, R.P.J., Lidke, K.A., Bates, M., Puig, D.L., Grünwald, D., Stallinga, S., and Rieger, B. (2013). Measuring image resolution in optical nanoscopy. Nat. Methods *10*, 557–562.

Olivier, N., Keller, D., Gönczy, P., and Manley, S. (2013). Resolution Doubling in 3D-STORM Imaging through Improved Buffers. PLoS One *8*.

Palmer, D.J., Helms, J.B., Beckers, C.J.M., Orci, L., and Rothman, J.E. (1993). Binding of coatomer to Golgi membranes requires ADP-ribosylation factor. J. Biol. Chem. *268*, 12083–12089.

Parthasarathy, R. (2012). Rapid, accurate particle tracking by calculation of radial symmetry centers. Nat. Methods *9*, 724–726.

Tsin, Y., and Kanade, T. (2004). A correlation-based approach to robust point set registration. In Computer Vision-ECCV 2004, (Springer), pp. 558–569.

Wang, B., and Booth, M.J. (2009). Optimum deformable mirror modes for sensorless adaptive optics. Opt. Commun. *282*, 4467–4474.

Wang, Y., Schnitzbauer, J., Hu, Z., Li, X., Cheng, Y., Huang, Z.-L., and Huang, B. (2014). Localization events-based sample drift correction for localization microscopy with redundant cross-correlation algorithm. Opt. Express *22*, 15982–15991.
