# Supplementary figures and images for: Ultra-High Resolution 3D Imaging of Whole Cells
Source: Cell. 2016 Aug 11;166(4):1028–40. doi: 10.1016/j.cell.2016.06.016 (PMC5005454; doi:10.1016/j.cell.2016.06.016)

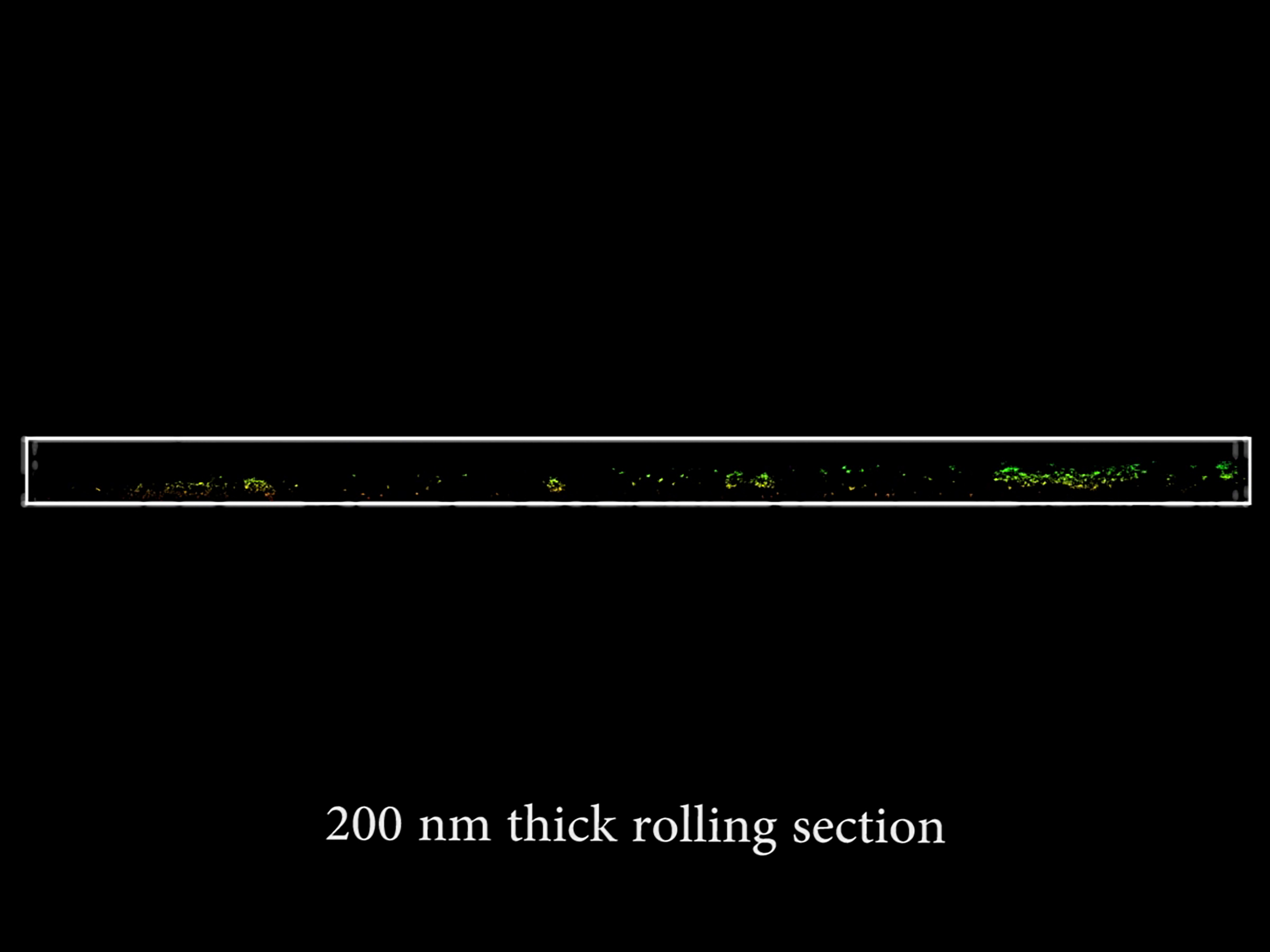

Supplement: Movie S1. Endoplasmic Reticulum Immunolabeled Overexpressed mEmerald-Sec61β in a COS-7 Cell, Related to Figure 1 [file mmc2.jpg]

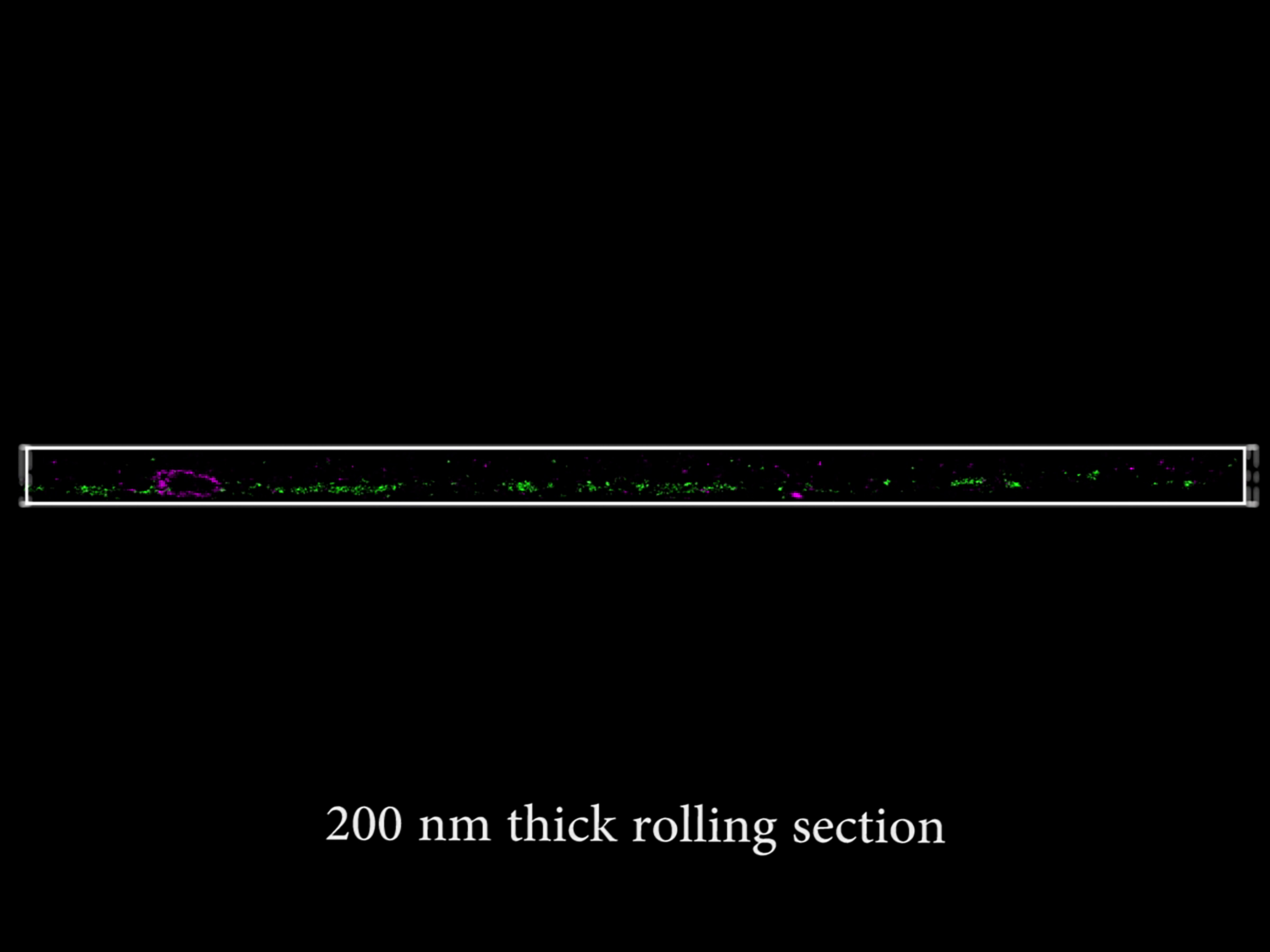

Supplement: Movie S2. Mitochondria and Microtubules Immunolabeled TOM20 and α-Tubulin in a COS-7 Cell, Related to Figure 2 [file mmc3.jpg]

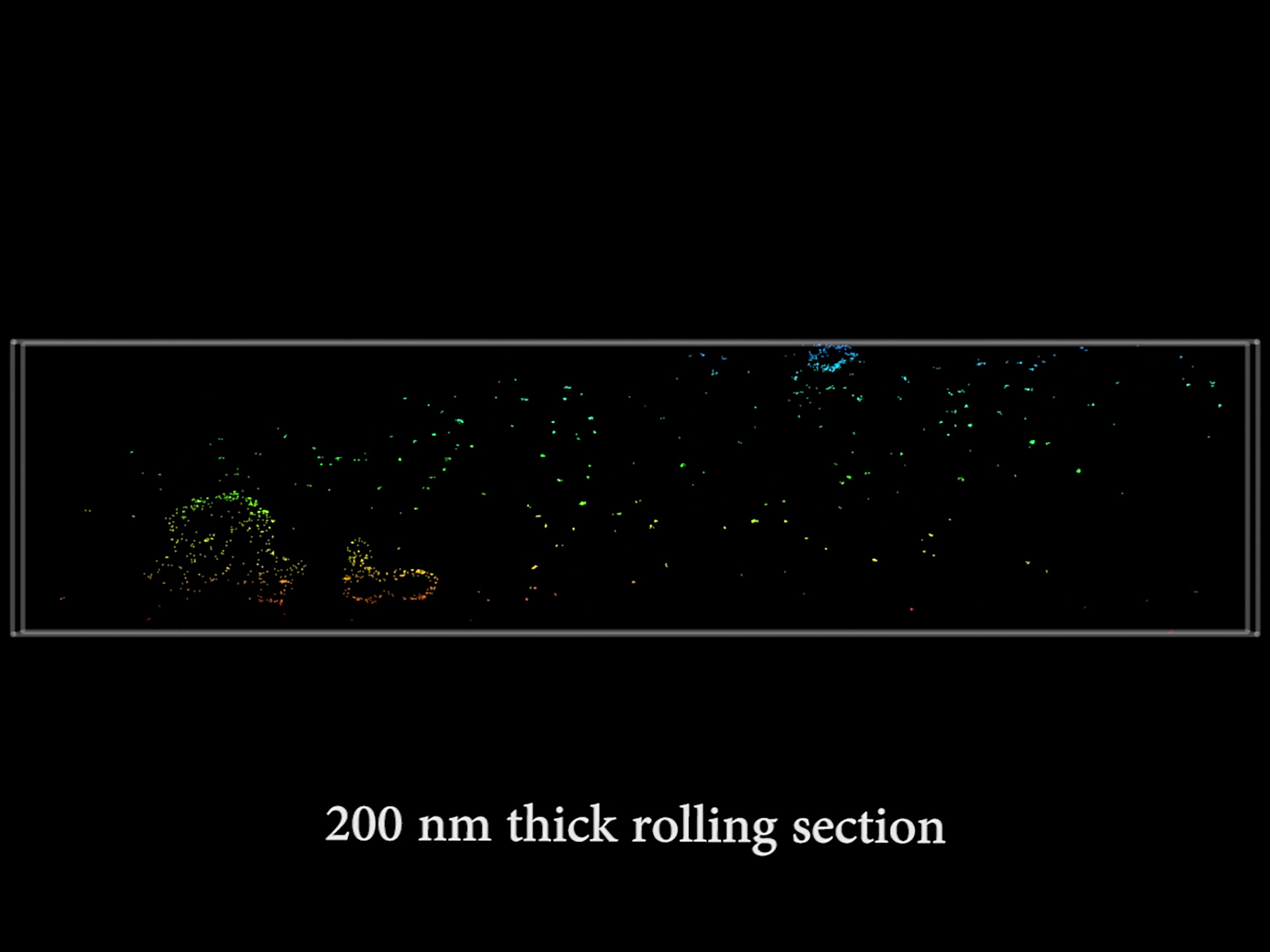

Supplement: Movie S3. Mitochondria Immunolabeled TOM20 in a COS-7 Cell, Related to Figure 3 [file mmc4.jpg]

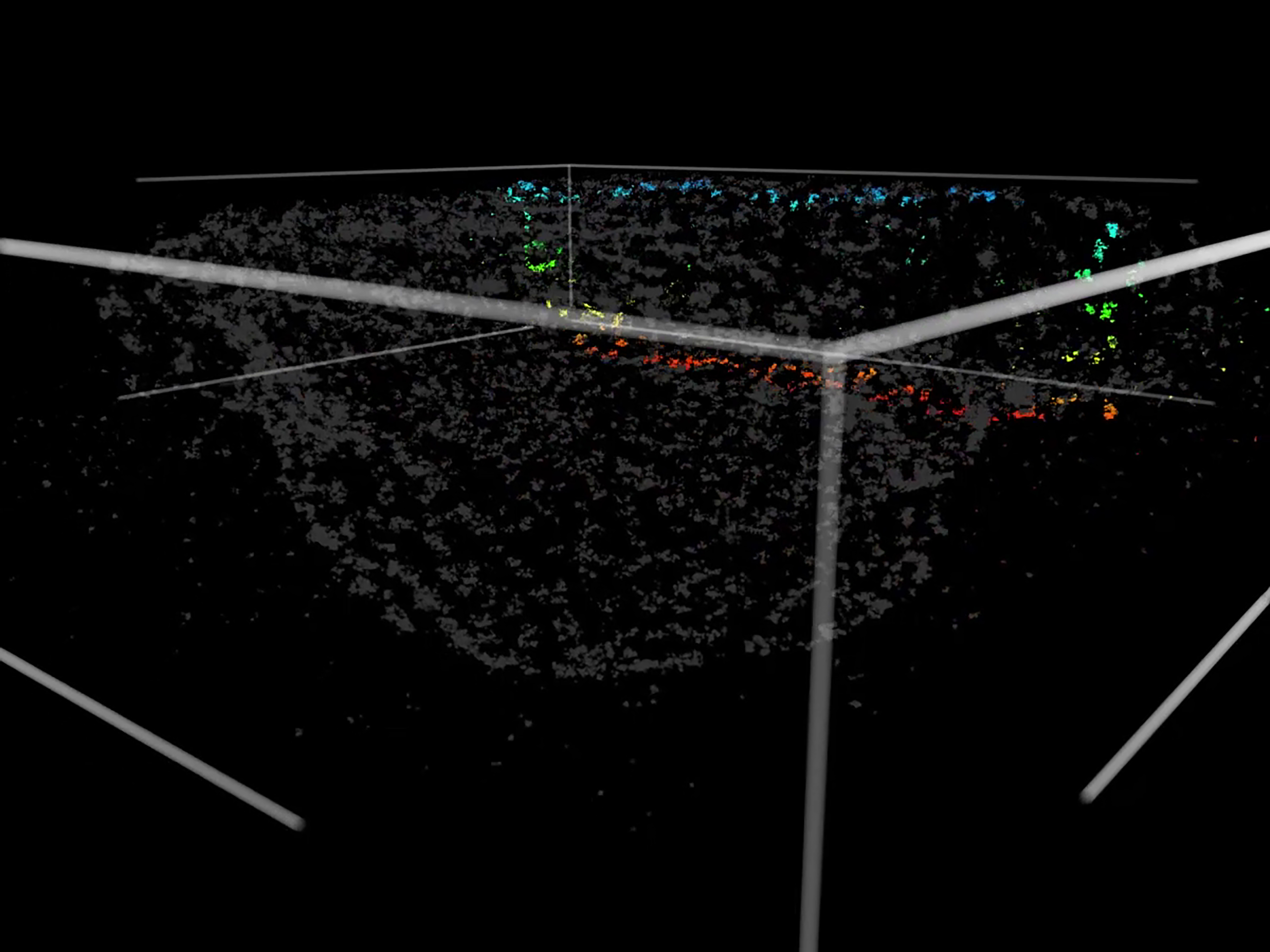

Supplement: Movie S4. Nucleus Immunolabeled Nuclear Pore Complexes in an hTERTRPE1 Cell, Related to Figure 4 [file mmc5.jpg]

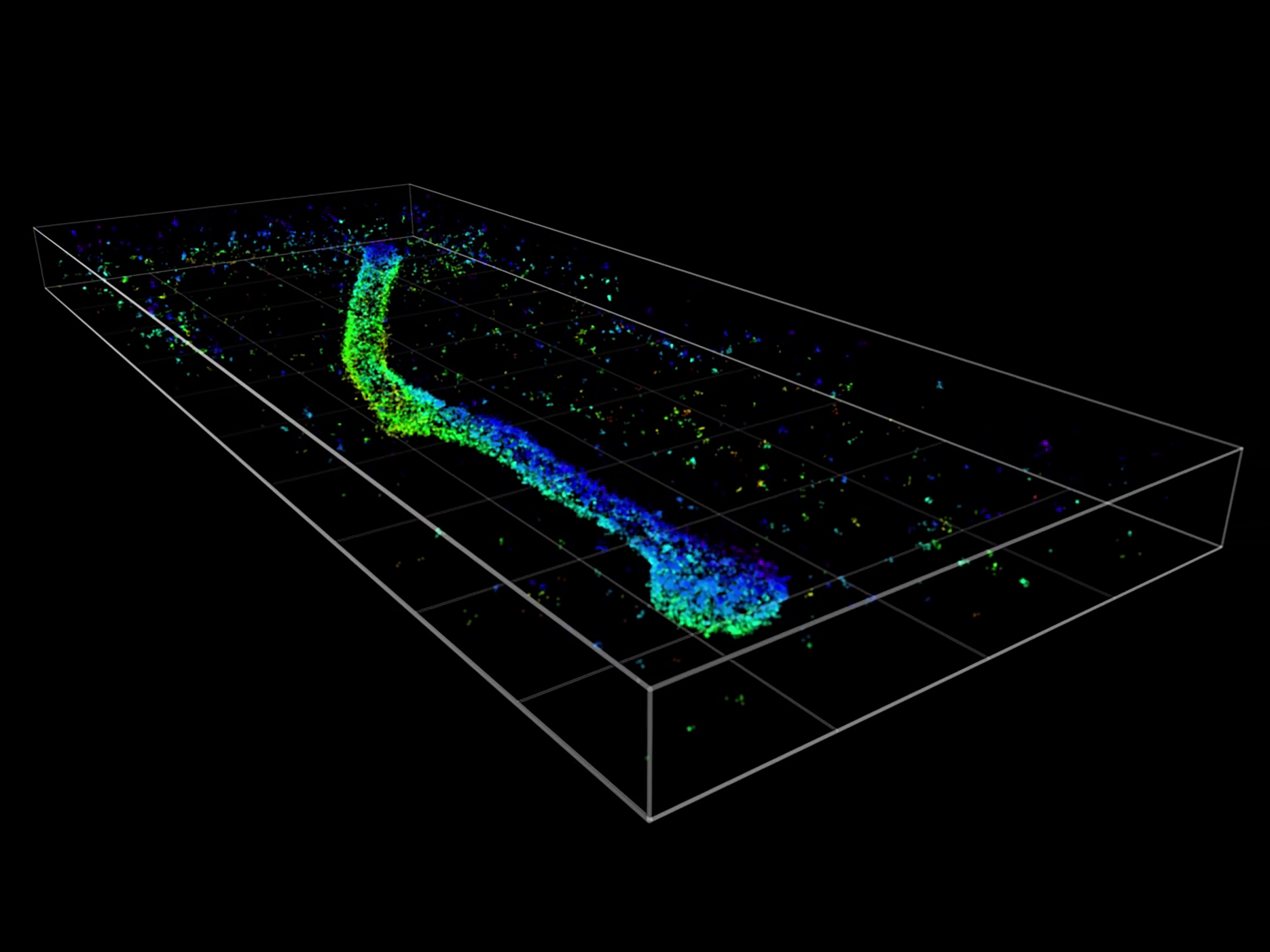

Supplement: Movie S5. Primary Cilium Immunolabeled pHluorin-Smoothened Expressed in an hTERT-RPE1 Cell, Related to Figure 6 [file mmc6.jpg]

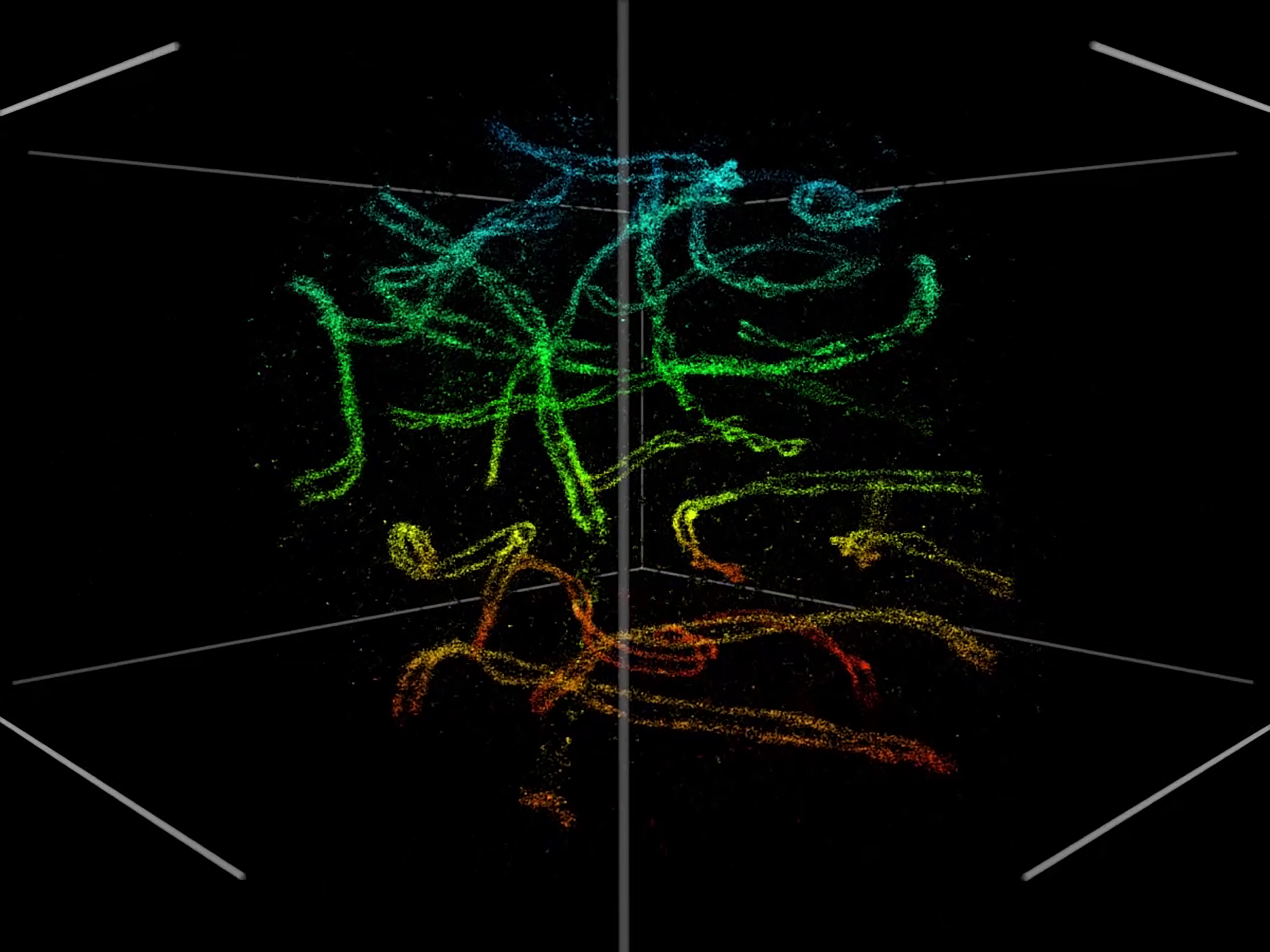

Supplement: Movie S6. Synaptonemal Complex Immunolabeled Synaptonemal Complex Protein 3 in a Mouse Spermatocyte, Related to Figure 7 [file mmc7.jpg]

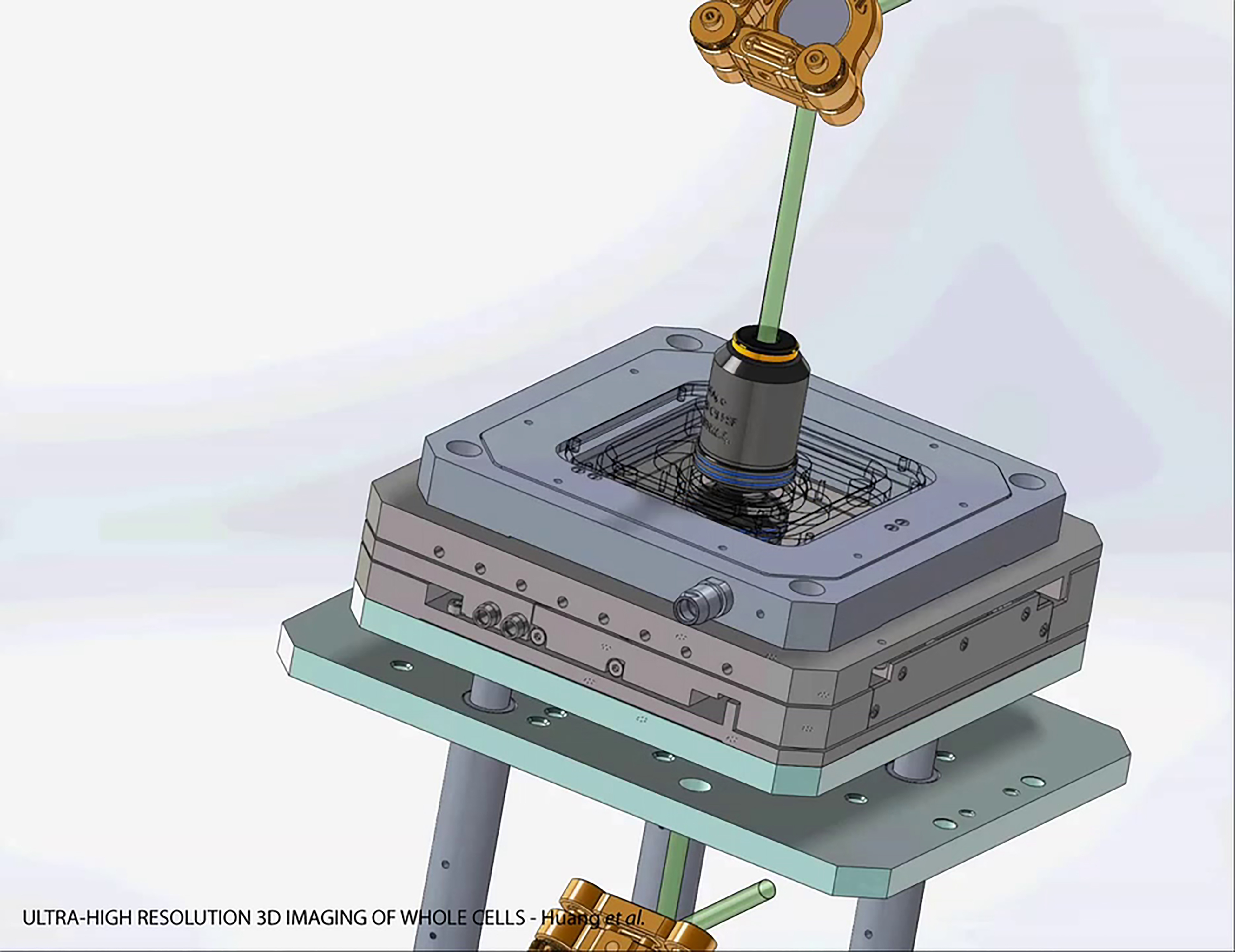

Supplement: Movie S7. Animation of the W-4PiSMSN Setup, Related to Figure 1 and Experimental Procedures [file mmc8.jpg]
