# Supplementary material for: Ultra-High Resolution 3D Imaging of Whole Cells
Source: Cell. 2016 Aug 11;166(4):1028–40. doi: 10.1016/j.cell.2016.06.016 (PMC5005454; doi:10.1016/j.cell.2016.06.016)
Supplement: Document S2. Article plus Supplemental Information [file mmc9.pdf]

Cell

Resource

Ultra-High Resolution 3D Imaging of Whole Cells

Graphical Abstract

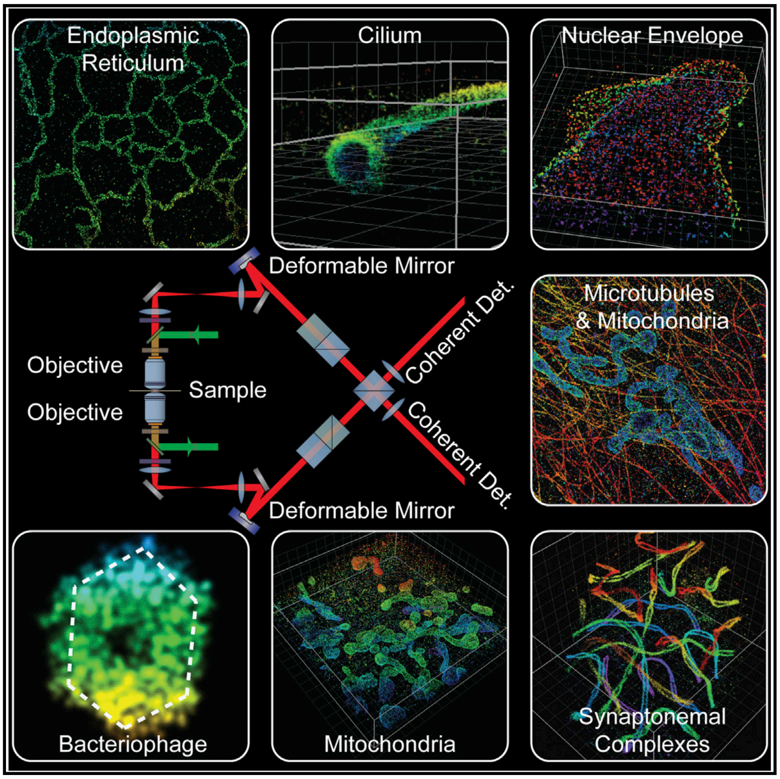

Highlights

- Whole-cell 4Pi single-molecule switching nanoscopy allows 10- to 20-nm 3D resolution
- Refined hardware and new data analysis allow imaging of cells as thick as ~10 μm
- Using structure-averaging, the 3D shape of a bacteriophage can be resolved
- Wide applicability across diverse research fields is demonstrated

Authors

Fang Huang, George Sirinakis, Edward S. Allgeyer, ..., Derek Toomre, Martin J. Booth, Joerg Bewersdorf

Correspondence

joerg.bewersdorf@yale.edu

In Brief

A new super-resolution microscope opens a window deep into cells to image organelles and subcellular structures spanning large volumes.

Huang et al., 2016, Cell *166*, 1028–1040
August 11, 2016 © 2016 The Authors. Published by Elsevier Inc.
http://dx.doi.org/10.1016/j.cell.2016.06.016

Resource

Ultra-High Resolution 3D Imaging of Whole Cells

Fang Huang,[1,2,14] George Sirinakis,[1,3,14] Edward S. Allgeyer,[1,3] Lena K. Schroeder,[1] Whitney C. Duim,[1,4] Emil B. Kromann,[1,5] Thomy Phan,[1] Felix E. Rivera-Molina,[1] Jordan R. Myers,[1] Irnov Irnov,[6,7] Mark Lessard,[8] Yongdeng Zhang,[1] Mary Ann Handel,[8] Christine Jacobs-Wagner,[6,7,9,10] C. Patrick Lusk,[1] James E. Rothman,[1,11] Derek Toomre,[1,11] Martin J. Booth,[12,13] and Joerg Bewersdorf[1,5,11,*]

[1]Department of Cell Biology, School of Medicine, Yale University, New Haven, CT 06520, USA
[2]Weldon School of Biomedical Engineering, Purdue University, West Lafayette, IN 47907, USA
[3]The Gurdon Institute, University of Cambridge, Cambridge CB2 1QN, UK
[4]Department of Chemistry, Harvey Mudd College, Claremont, CA 91711, USA
[5]Department of Biomedical Engineering, Yale University, CT 06520, USA
[6]Microbial Sciences Institute, Yale University, West Haven, CT 06516, USA
[7]Department of Molecular, Cellular and Developmental Biology, Yale University, New Haven, CT 06520, USA
[8]The Jackson Laboratory, Bar Harbor, ME 04609, USA
[9]Howard Hughes Medical Institute, Yale University, New Haven, CT 06520, USA
[10]Department of Microbial Pathogenesis, Yale School of Medicine, New Haven, CT 06520, USA
[11]Nanobiology Institute, Yale University, West Haven, CT 06516, USA
[12]Department of Engineering Science, University of Oxford, Oxford OX1 3PJ, UK
[13]Centre for Neural Circuits and Behaviour, University of Oxford, Oxford OX1 3SR, UK
[14]Co-first author
*Correspondence: joerg.bewersdorf@yale.edu
http://dx.doi.org/10.1016/j.cell.2016.06.016

SUMMARY

Fluorescence nanoscopy, or super-resolution microscopy, has become an important tool in cell biological research. However, because of its usually inferior resolution in the depth direction (50–80 nm) and rapidly deteriorating resolution in thick samples, its practical biological application has been effectively limited to two dimensions and thin samples. Here, we present the development of whole-cell 4Pi single-molecule switching nanoscopy (W-4PiSMSN), an optical nanoscope that allows imaging of three-dimensional (3D) structures at 10- to 20-nm resolution throughout entire mammalian cells. We demonstrate the wide applicability of W-4PiSMSN across diverse research fields by imaging complex molecular architectures ranging from bacteriophages to nuclear pores, cilia, and synaptonemal complexes in large 3D cellular volumes.

INTRODUCTION

Major advances in cell biology are tightly linked to innovations in microscopy. The development of fluorescence microscopy, for example, enabled sub-cellular localization of specifically labeled proteins of interest (Lichtman and Conchello, 2005). However, the wave nature of light restricts the resolution of conventional light microscopy to ~200 nm, making details of subcellular structures and protein assemblies unresolvable (Hell, 2007). The advent of super-resolution fluorescence microscopy, or nanoscopy, techniques such as stimulated emission depletion (STED) (Hell and Wichmann, 1994) and single-molecule switching nanoscopy (SMSN) (Betzig et al., 2006; Hess et al., 2006; Rust et al., 2006) has extended the application range of fluorescence microscopy beyond the diffraction limit, achieving up to 10-fold improvement in resolution (Gould et al., 2012a). These methods are now maturing and offering the opportunity to observe biological phenomena never before seen (Chojnacki et al., 2012; Kanchanawong et al., 2010; Liu et al., 2011; Xu et al., 2013). Nanoscopy techniques share a common principle: they spatially separate unresolvable fluorescent molecules by independently switching their emission "on" and "off" (Hell, 2007). In particular, SMSN methods such as photoactivated localization microscopy (PALM), fluorescence photoactivation localization microscopy (FPALM), and stochastic optical reconstruction microscopy (STORM) use a stochastic approach where only a small subset of fluorescent molecules is switched on at any particular moment in time while the majority remains in a non-fluorescent "dark" or "off" state (Gould et al., 2012a). Super-resolved images are reconstructed from the positions of thousands to millions of single molecules that have been recorded in thousands of camera frames.

This imaging strategy was initially applied to single-objective microscopes in two dimensions (2D) (Betzig et al., 2006; Hess et al., 2006; Rust et al., 2006) and later extended to three dimensions (3D) (Huang et al., 2008; Juette et al., 2008; Pavani et al., 2009). While these instruments achieve 20- to 40-nm resolution in the focal plane (lateral, x-y), the resolution in the depth direction (axial, z) is typically limited to only 50–80 nm. The resolution can, however, be further improved by using a dual-objective "4Pi" detection geometry (Bewersdorf et al., 2006).

Using two objectives doubles the detection efficiency (Xu et al., 2012) and thus improves the localization precision ~1.4-fold in all three dimensions. Additionally, employing two objectives in a 4Pi geometry allows the creation of a single-molecule emission interference pattern at the detector leading to an

 © 2016 The Authors. Published by Elsevier Inc.
This is an open access article under the CC BY license (http://creativecommons.org/licenses/by/4.0/).

~7-fold improvement in axial localization precision over single-objective approaches as demonstrated using interferometric PALM (iPALM) (Shtengel et al., 2009) and 4Pi single marker switching nanoscopy (4Pi-SMSN) (Aquino et al., 2011). This improved resolution enabled, for example, the generation of anatomical maps of focal adhesions at ~10-nm axial resolution (Case et al., 2015; Kanchanawong et al., 2010). However, this method was initially restricted to samples of ~250 nm in thickness (Shtengel et al., 2009) and more recently to 700–1,000 nm (Aquino et al., 2011; Brown et al., 2011). As the typical thickness of a mammalian cell is 5–10 μm, this has limited optical microscopy at the ~10-nm isotropic resolution scale to thin sub-volumes of cells, thus precluding the ability to image organelles that can extend over several microns throughout the whole cell.

Here, we present a new implementation of iPALM/4Pi-SMSN, termed whole-cell 4Pi single-molecule switching nanoscopy (W-4PiSMSN), which extends the imaging capabilities of this technology to whole cells without compromising resolution. W-4PiSMSN allows volumetric reconstruction with 10- to 20-nm isotropic resolution of ~10-μm-thick samples, a 10- to 40-fold improvement in sample thickness over previous iPALM/4Pi-SMSN implementations (Aquino et al., 2011; Brown et al., 2011; Van Engelenburg et al., 2014; Shtengel et al., 2009). Our approach permits ultra-high resolution 3D imaging of virtually any subcellular structure. To demonstrate this, we image the endoplasmic reticulum (ER), bacteriophages, mitochondria, nuclear pore complexes, primary cilia, Golgi-apparatus-associated COPI vesicles, and mouse spermatocyte synaptonemal complexes. By these examples, we show that W-4PiSMSN opens the door to address cell biological questions that were previously unanswerable.

RESULTS

Development of W-4PiSMSN

To realize a system that achieves 10- to 20-nm 3D resolution across the thickness of entire mammalian cells, we expanded on previous iPALM and 4Pi-SMSN developments (Aquino et al., 2011; Shtengel et al., 2009). In these systems, fluorescence emission is collected by two opposing objective lenses and combined to interfere (Aquino et al., 2011; von Middendorff et al., 2008; Shtengel et al., 2009). Depending on the axial position of a molecule, the light will interfere constructively or destructively, as indicated by the brightness of the molecule's image on the detector. However, molecules at axial positions that differ by multiples of half the wavelength of light lead to the same interference pattern and cause ambiguity in determining their axial positions. This localization ambiguity leads to scrambled images that contain axially shifted image artifacts, known as ghost images, in samples thicker than ~250 nm. This can be avoided by using not only the brightness but also the z-position-dependent shape of the single-molecule images to determine a molecule's axial position. To address this, a higher-moment based analysis (Aquino et al., 2011) and analysis of the point-spread function (PSF) eccentricity in a hyperbolic mirrors-modified system (Brown et al., 2011) were developed, which extended the image volume thickness to 700–1,000 nm. However, these methods pose significant drawbacks such as poor localization density because of the highly selective computational processes focusing on subtle features of PSFs. The methods are also susceptible to sample-induced optical aberrations, which change the PSF shape when imaging biological structures deeper in the sample (Burke et al., 2015; von Diezmann et al., 2015). As a result, applications have been restricted to thin structures close to the coverslip (Case et al., 2015; Van Engelenburg et al., 2014; Kanchanawong et al., 2010).

To enable 4Pi-SMSN to probe deeper into the cell and extend the application of this technology to larger cellular features, we have developed W-4PiSMSN. First, expanding on the optical design by Aquino et al. (2011), we included deformable mirrors in both arms of the 4Pi-interferometric cavity (Figure 1A; Figure S1). We use these mirrors to correct for imperfections in the instrument beam path and optimize the PSF quality for samples with various thicknesses (Supplemental Information; Figure S2). Deformable mirrors also allow us to compensate for sample-induced aberration modes, such as spherical aberrations (Burke et al., 2015; Gould et al., 2012b), which vary from sample to sample and with depth. Additionally, we can use these mirrors to introduce astigmatism in both interference arms without adding further complexity to the system (Supplemental Information). Thus, the deformable mirrors enable compromise-free, reproducible PSFs in a depth- and sample-independent manner.

Second, building on an earlier approach by Brown et al. (2011), we developed an analysis method that combines information from (1) the 4Pi-PSF's interference phase, which allows for precise axial localization but does not distinguish between different interference peaks, and (2) the eccentricity of the astigmatic 4Pi-PSF, which narrows axial localizations down to individual interference peaks but in itself does not offer the precision of 4Pi interference. Our new analysis algorithm interprets the large number of molecules imaged in each time and z-depth window as an *ensemble measurement* of the concurrent state of the W-4PiSMSN system (Supplemental Information; Figure S3A) and determines the relationship between the eccentricity of the astigmatic PSF and the interference phase of the 4Pi-PSF. Then the axial positions of all corresponding molecules can be assigned with high precision and unambiguously using a monotonic metric designed to describe the overall shape of the PSF and maintain its monotonicity in the presence of moderate amounts of aberrations (Supplemental Information; Figure S3B). Since this analysis is performed for well-defined temporal and axial data subsets, we have generalized it to identify and correct for drift (from both the system and the sample) over the course of imaging. Our method is robust against aberrations and improves the reliability and efficiency of axial position assignment as it automatically adapts to changes in the shape and interference pattern of the 4Pi-PSF.

Ultra-High Resolution Imaging with W-4PiSMSN

To demonstrate the resolution capabilities of our new system, we first imaged the ER. ER membranes were stained using anti-GFP antibodies to the overexpressed transmembrane protein, mEmerald-Sec61β, in COS-7 cells. We visualized the ER as a connected network of hollow tubes with 60–100 nm diameters (Figures 1B and 1C; Movie S1). Both horizontal and vertical

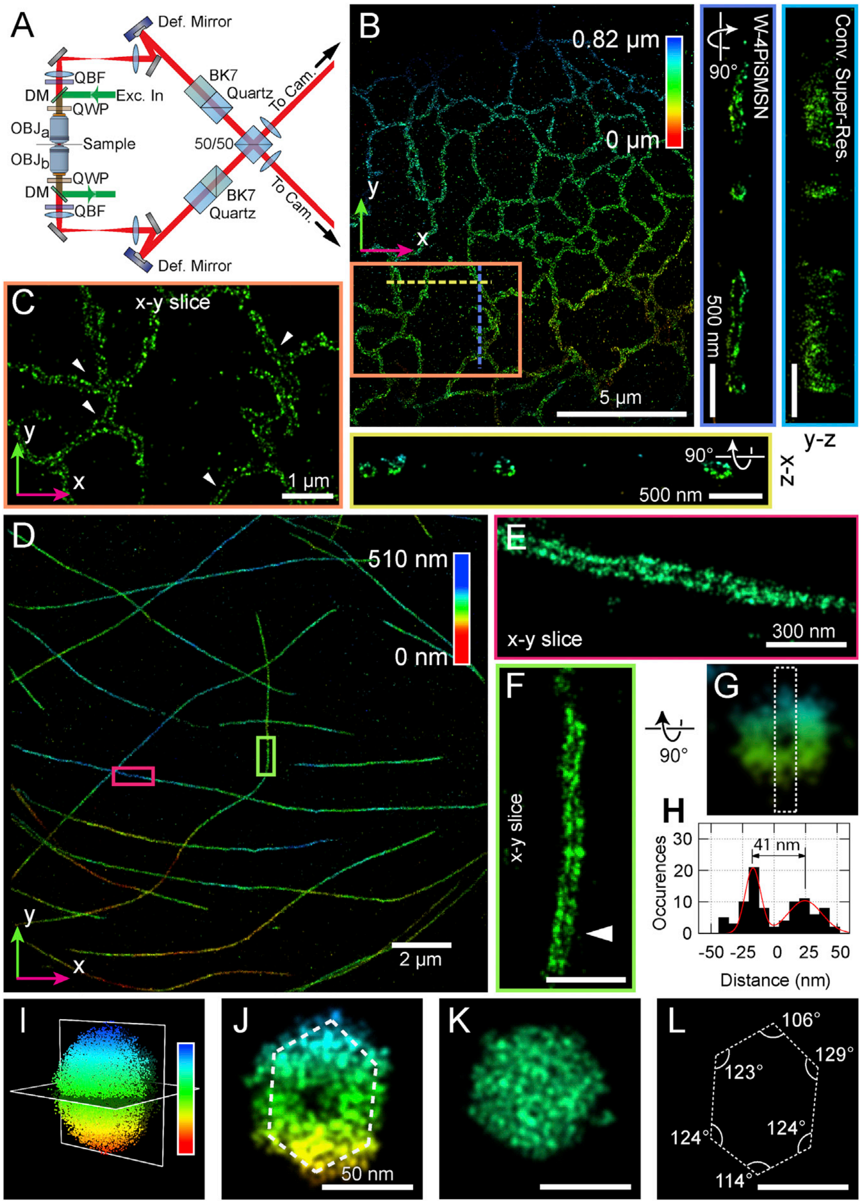

Figure 1. W-4PiSMSN Design and Resolution Demonstrations with ER, Microtubules, and Bacteriophages

(A) Simplified optical diagram of W-4PiSMSN.

(B) Overview and cross-sections of the ER network in an immunolabeled COS-7 cell. Cross-sections of the W-4PiSMSN reconstruction show clearly separated membranes of the tubular structures, which cannot be resolved with conventional astigmatism-based nanoscopy (light blue frame).

(C) x-y slice through the mid-section of the ER network shown in (B) highlights the distinct membrane contour of ER tubules (arrowheads).

(D) Overview of immunolabeled microtubules in a COS-7 cell.

(E and F) 20-nm-thin x-y slices of the red (E) and green (F) segments shown in (D) demonstrate that microtubules can be easily resolved as hollow cylinders in W-4PiSMSN.

(G) A look through a 120-nm-long segment of the microtubule of (F).

(H) A histogram showing the number of localizations in a cross-section of the microtubule, white dotted box in (G).

(I) A bacteriophage reconstructed from 115 averaged viral particles rendered in 3D.

(J and K) 5-nm-thin vertical (J) and horizontal (K) slices through the averaged dataset corresponding to the planes shown in (I).

(L) The internal angle measurements of the hexagon shape identified from the viral capsid shown in (J).

OBJ, objective; QWP, quarter-wave plate; DM, dichroic mirror; QBF, quad-band band-pass filter; Def. Mirror, deformable mirror; Cam, camera; 50/50, beam splitter cube.

cross-sections reveal the 3D membrane contour that was previously resolvable only with electron tomography (Frey and Mannella, 2000). This high 3D resolution is quantitatively supported by a Fourier Shell Correlation value of 22 nm (Figure S4) (Nieuwenhuizen et al., 2013). To test our approach on even smaller structures, we imaged antibody-labeled microtubules in COS-7 cells, a gold standard in SMSN (Figures 1D–1H). Without any detectable imaging artifacts, W-4PiSMSN resolves this 25-nm microtubule filament, which appears in all orientations as a hollow core coated with antibodies (Figures 1E–1H). In addition, the dataset features a high localization density of ~5.5 localization events per 10 × 10 nm^2 of surface area. Displaying the localization events by their radial distance from the tubule axis shows a Gaussian peak with a full width at half maximum (FWHM) of 16–24 nm (Figure S4). Considering that the use of primary and secondary antibodies adds uncertainty to the actual position of the imaged dye molecules, we conclude that the 3D resolution of our instrument is well below 20 nm (FWHM).

To demonstrate our approach on another challenging target, we imaged T7 bacteriophages. They feature an icosahedral-shaped capsid of ~60-nm diameter, which has only been visible by cryo-electron microscopy (cryo-EM) techniques before (Hu et al., 2013). We non-specifically labeled proteins on the surface of purified T7 phages using an Alexa Fluor 647 NHS Ester, which reacts with primary amines, and mounted the phages on a coverslip (Figure S5). Image slices of a single phage in the x-y, y-z, and x-z directions show a hollow center in all orientations. To further refine the details of the detected phage structures, we adapted the tomogram-averaging approach originally developed for cryo-EM (Briggs, 2013; Broeken et al., 2015). By combining 115 T7 images, our averaged reconstruction reveals the icosahedral shape of the T7 phages (Figures 1I–1L; Figure S4). As presented in Figures 1J–1L, a slice perpendicular to the major axis shows the expected pentagonal shape while a slice parallel to the major axis reveals a hexagonal shape. Our approach, however, has yet to clearly resolve the

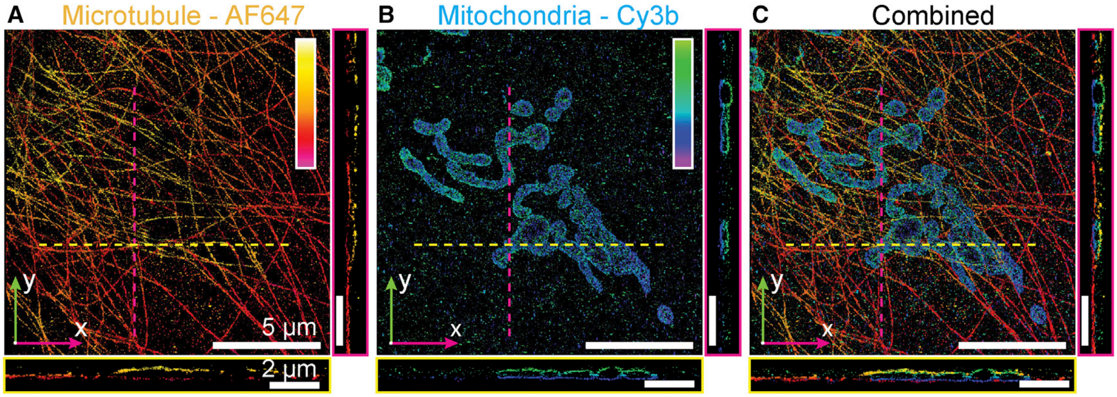

Figure 2. Two-Color Reconstruction of Mitochondria and Microtubules

(A and B) W-4PiSMSN reconstruction of microtubules (A) and mitochondria (TOM20) (B) in a COS-7 cell immunolabeled with Alexa Fluor 647 and Cy3B, respectively. An x-y overview and x-z and y-z slices (yellow and magenta lines, respectively) are shown.

(C) The combined two-color image reveals microtubules running adjacent to the mitochondria surface.

~23-nm tail and fiber structures of the T7 phage (Hu et al., 2013). This is likely due to either incomplete labeling of the surface proteins or the flexibility of these structures. Nonetheless, our W-4PiSMSN system has enabled the visualization of the ultrastructure of bacteriophages using light microscopy.

We tested the two-color imaging capability of W-4PiSMSN by imaging microtubules and mitochondria in a COS-7 cell immunolabeled with Alexa Fluor 647 and Cy3B, respectively. Our reconstructions show microtubules running in close proximity ~10–20 nm from the mitochondria top and bottom surfaces (Figures 2A–2C; Movie S2; Figure S5). Our system decouples axial localization from the PSF shape, the latter being susceptible to depth-dependent distortions caused by sample-induced optical aberrations (Liu et al., 2013; McGorty et al., 2014). While single-objective systems rely on the PSF shape, the W-4PiSMSN approach uses the relative interference amplitudes to determine the axial location of individual molecules. However, multicolor imaging is challenging because the spatial interference modulation frequency is wavelength dependent and differs between color channels. To address this, we derived the modulation frequency using a pupil-function based approach (Supplemental Information). Our simulation results were verified experimentally by registering two color channels from an affine transformation matrix, which was calibrated using two-color-labeled mitochondria in fixed cells (Supplemental Information).

Whole-Cell 3D Imaging with W-4PiSMSN

Imaging volumes thicker than ~1.2 µm requires axial sample scanning, because molecules more than ~600 nm out of focus cannot be identified and localized efficiently (Huang et al., 2008; Juette et al., 2008). Thus, optical sections must be recorded at different axial sample positions and subsequently merged to obtain the complete cellular volume. Compared to conventional 3D nanoscopes, the superior localization precision of W-4PiSMSN puts high demands on the localization accuracy in each volume section (i.e., avoiding volume distortions) and the merging process. In the section-merging process, small misalignments of neighboring optical sections caused by sample-induced aberrations or drift can lead to significant deterioration of the resolution and distortions of the super-resolved volume (Huang et al., 2008; Mlodzianoski et al., 2011).

We designed our system to minimize drift: our instrument design takes advantage of a horizontal symmetry plane coinciding with the common focal planes of the objectives and the beam splitter cube of the interference cavity. This symmetric design desensitizes the interferometric cavity of the microscope to temperature changes leading to approximately equal thermal expansion in both arms of the interference cavity. To compensate for any remaining instrument and sample drift caused by mechanical and thermal fluctuations, we developed a set of hardware and software tools (Figure S6). The objectives are stabilized in 3D relative to each other by focusing a near-infrared laser beam by one objective and detecting the focus with the other objective in a "biplane" configuration (Figure S6) (Juette et al., 2008; Ram et al., 2008). This allows the detection of relative objective movement in 3D, which can then be compensated for via a feedback loop. Furthermore, we cross-correlate 3D volume data segments of 1- to 2-min windows using a redundancy-based drift correction method (Li et al., 2013; Wang et al., 2014) in an extended correlation volume. Within each of these short data segments, an independent relationship between astigmatism and interference phase is established. Any discrepancies between these relationships for different segments are treated as drift (Supplemental Information). The above-described methods enable us to fully compensate for sample and instrument drift and changes in the optical path between the two arms of the interferometric 4Pi cavity due to the axial scanning nature of the measurements.

To demonstrate the whole-cell imaging capabilities of the W-4PiSMSN system, we imaged mitochondria using antibodies against the outer membrane protein TOM20 over the whole thickness of a COS-7 cell. Figure 3 reveals the outer membrane contour and the remarkably interconnected mitochondrial network over a depth of 4.3 µm (Figures 3B–3D; Movie S3). We were not able to detect any significant ghost images within the volume (Figures 3A–3D).

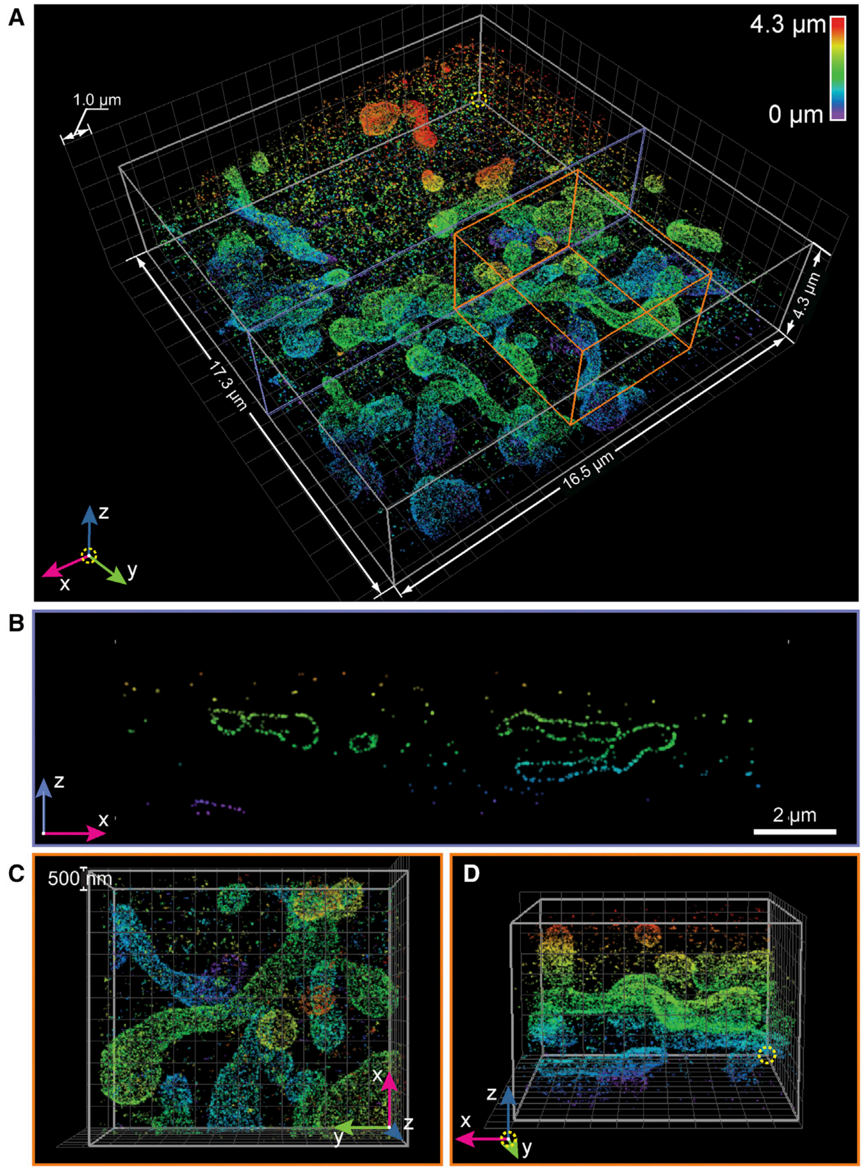

Figure 3. W-4PiSMSN Reconstruction of TOM20 on Mitochondria in COS-7 Cell

(A) Overview of the mitochondria network visualized by immunolabeling TOM20 with Alexa Flour 647. The dataset is assembled from 11 optical sections with 500-nm step sizes.

(B) x-z cross-section of the purple plane in (A) showing the distribution of TOM20 on the outer mitochondrial membrane. Ghost images are completely negligible.

(C and D) Top (C) and side (D) views of the orange box in (A) show the 3D arrangement of the organelle.

To further demonstrate that image quality is maintained throughout the thickness of whole cells, we imaged nuclear pore complexes (NPCs) on the nuclear envelope. By immunolabeling with an antibody that recognizes a component of the cytoplasmic filaments (Nup358) of NPCs (von Appen et al., 2015), we can reconstruct NPCs on the top, side, and bottom of the nucleus (Figure 4; Movie S4). As with mitochondria, our approach reveals the contours of almost the entire nuclear surface, where both prominent invaginations and subtle undulations (typically visualized only by electron microscopy [EM]) are apparent (Figures 4A and 4B).

Revealing Golgi-Apparatus-Associated COPI Vesicles

We next imaged COPI vesicles, which have traditionally been resolved only by EM as they have ~100-nm diameters and are densely packed around the Golgi cisternae (Orci et al., 1997). Moreover, as the Golgi complex is located close to the middle of the cell, recording high-quality data in a central z-plane is a challenging test of the instrument's 3D resolution capabilities. Figure 5 shows the β' COP, a protein in the outer COPI complex, immunostained using Alexa Fluor 647 in BSC-1 cells. Strikingly, we visualized distinct hollow COPI-coated spheres within cells (Figures 5B, 5C, 5E, 5F, and 5G–5N). Our 3D images resolve individual COPI vesicles with ~100-nm diameter, consistent with previous measurements (Pellett et al., 2013). Additionally, a 300-nm-thick section shows that COPI-coated structures are packed around a 500- to 1,000-nm (x and y) by 500-nm (z) area devoid of COPI labeling, presumably containing a Golgi stack (Figures 5D–5F).

Revealing Ciliary Membrane GPCR Organization

Most high-resolution studies of the primary cilium, a solitary microtubule-based organelle that protrudes from the cell surface and acts as a cellular antenna, have relied on EM (Wood and Rosenbaum, 2015). A transmission EM image typically shows only a small subsection of a cilium as the sample is a random oblique ~70- to 100-nm-thick section through the structure, which can be up to 10 µm long and ~250 nm wide. Scanning EM images can easily show an entire cilium with high resolution; however, these images completely lack information about specific protein localization. Previous nanoscopy studies on cilia relied on inferring the 3D organization from 2D datasets (Yang et al., 2013, 2015). Here, we used W-4PiSMSN to image the G-protein-coupled receptor Smoothened (SMO) on whole primary cilia in hTERT-RPE1 cells with high 3D resolution (Figure 6; Movie S5). SMO was tagged with a pH-sensitive GFP (pH-SMO), which was used as an epitope for antibody labeling with Alexa Fluor 647 (Figure 6; Supplemental Information). We observe that overexpressed pH-SMO localizes to the membranes of cilia, which form hollow cylinders 3–10 µm long (Figure 6) and vary in

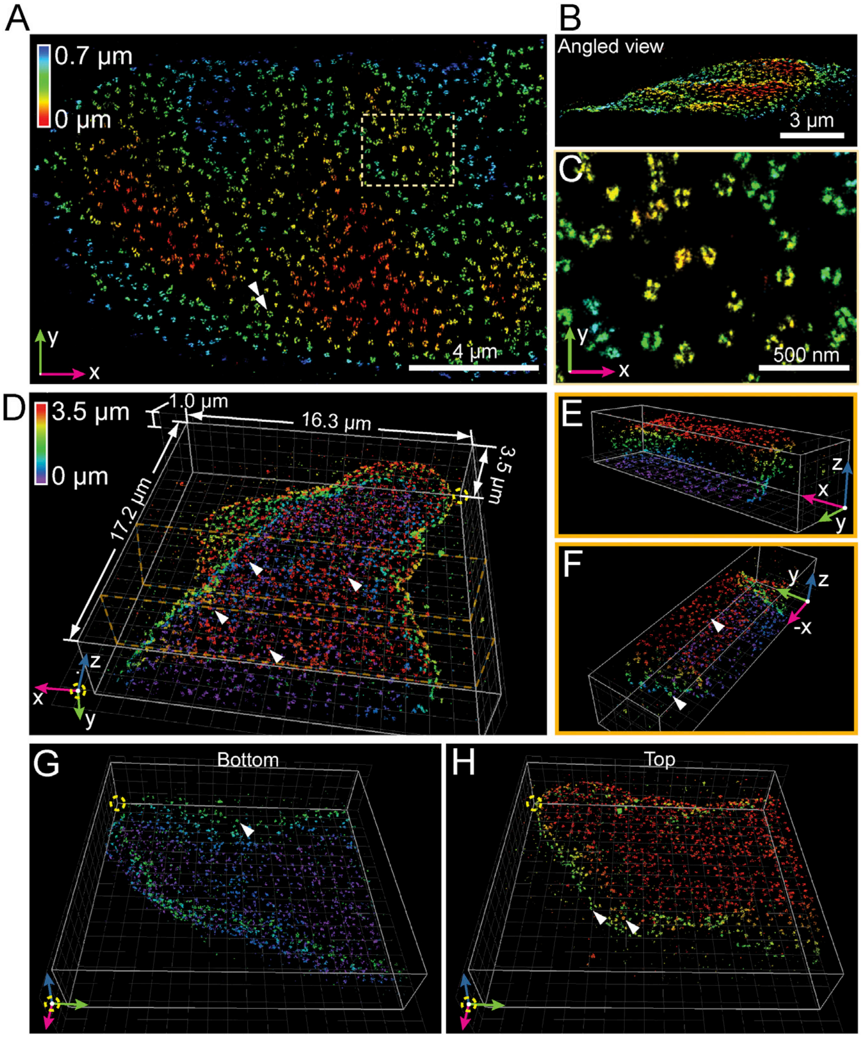

Figure 4. W-4PiSMSN Imaging of Nuclear Pore Complexes over the Thickness of a Cell Nucleus

Nup358 was Immunolabeled with Alexa Fluor 647 in hTERT-RPE1 cells.

(A) Overview of a region of the nucleus. The axial location of the nuclear pores is color coded.

(B) Side view of (A).

(C) A subregion indicated by the dashed box in (A) shows a zoomed in view of multiple nuclear pores.

(D) Overview of a 3D reconstruction of the nucleus obtained by combining nine optical sections.

(E) A section of the reconstruction in (D) confirms that the labeling is largely limited to the nuclear envelope.

(F) Different view of the same section.

(G and H) Bottom (G) and top (H) half of the nucleus shown in (D). The images reveal ring-like nuclear pores on the top and the bottom nuclear envelope as well as at the sides of the nucleus (arrowheads).

diameter from ~160 to 280 nm (Figures 6A–6E). Our W-4PiSMSN images of the ciliary membrane allow us to precisely measure the cilium's diameter along its entire length. Interestingly, we find that cilia diameters are not always constant. Rather, one example cilium shows an abrupt contraction of ~50 nm midway along its length (Figures 6C–6E; Movie S5). We speculate this change in diameter may correspond to the thinning of the 9+0 microtubule axoneme, which is known to transition from triplet microtubules, to doublets, and eventually singlets. The ciliary tip is not narrow but has a bulbous shape, consistent with structures observed in EM (He et al., 2014; Wang et al., 2013). The high-resolution reconstruction of the ciliary membrane also allowed us to "unwrap" the membrane tube into a flat surface (Figure 6H; Supplemental Information) permitting data quantification such as cluster analysis and co-localization measurement in a complex geometry. Next, we examined the local density of molecules along the ciliary membrane to identify regions with higher concentrations of pH-SMO. Higher local density is present around the base, on small bulbous protrusions, and on stripes along the cilium length (Figures 6F–6H; Figure S7). These protrusions may be vesicles (diameter ~150–200 nm) budding from the cilium (Figures 6F–6H), as ectosomes have been reported to bud from some cilia (Wood and Rosenbaum, 2015).

Resolving Synaptonemal Complexes in Whole-Mouse Spermatocytes

As a final demonstration of the capacity of our instrument to image deep into cells as thick as 10 μm, we stained synaptonemal complexes in mouse spermatocyte nuclei in the pachytene phase of meiotic prophase (Figure 7; Movies S6). While synaptonemal complexes have been imaged using structured illumination (Carlton, 2008; Qiao et al., 2012) and 4Pi microscopy at 100- to 200-nm resolution (Fritsche et al., 2012), higher resolution optical images have been limited to chromosome spreads of <1-μm thickness (Schücker et al., 2015). Here, we examined spermatocytes harvested from testes of 17- to 18-day-old mice with W-4PiSMSN and imaged the twisting band of the paired lateral elements of autosomal synaptonemal complexes, highlighted by immunolabeling synaptonemal complex protein 3 (SYCP3), a constituent component of the lateral elements (Page and Hawley, 2004). Reconstructed from a total of 126 optical sections (21 depth positions imaged in six repetition cycles), the entire 3D image spanned nearly 9 μm in depth and resolved SYCP3 substructure of the individual autosomal synaptonemal complexes with unprecedented clarity independent of their orientation or depth (Figures 7A–7E; Movie S6). Furthermore, 19 synaptonemal complexes representing pairs of individual autosomal homologs could be isolated using a Euclidian metric-based clustering algorithm on the individual single-molecule localizations (Supplemental Information). Thus, our approach promises the capacity to visualize the nanoscale spatial organization of chromosomal scaffolds in the context of

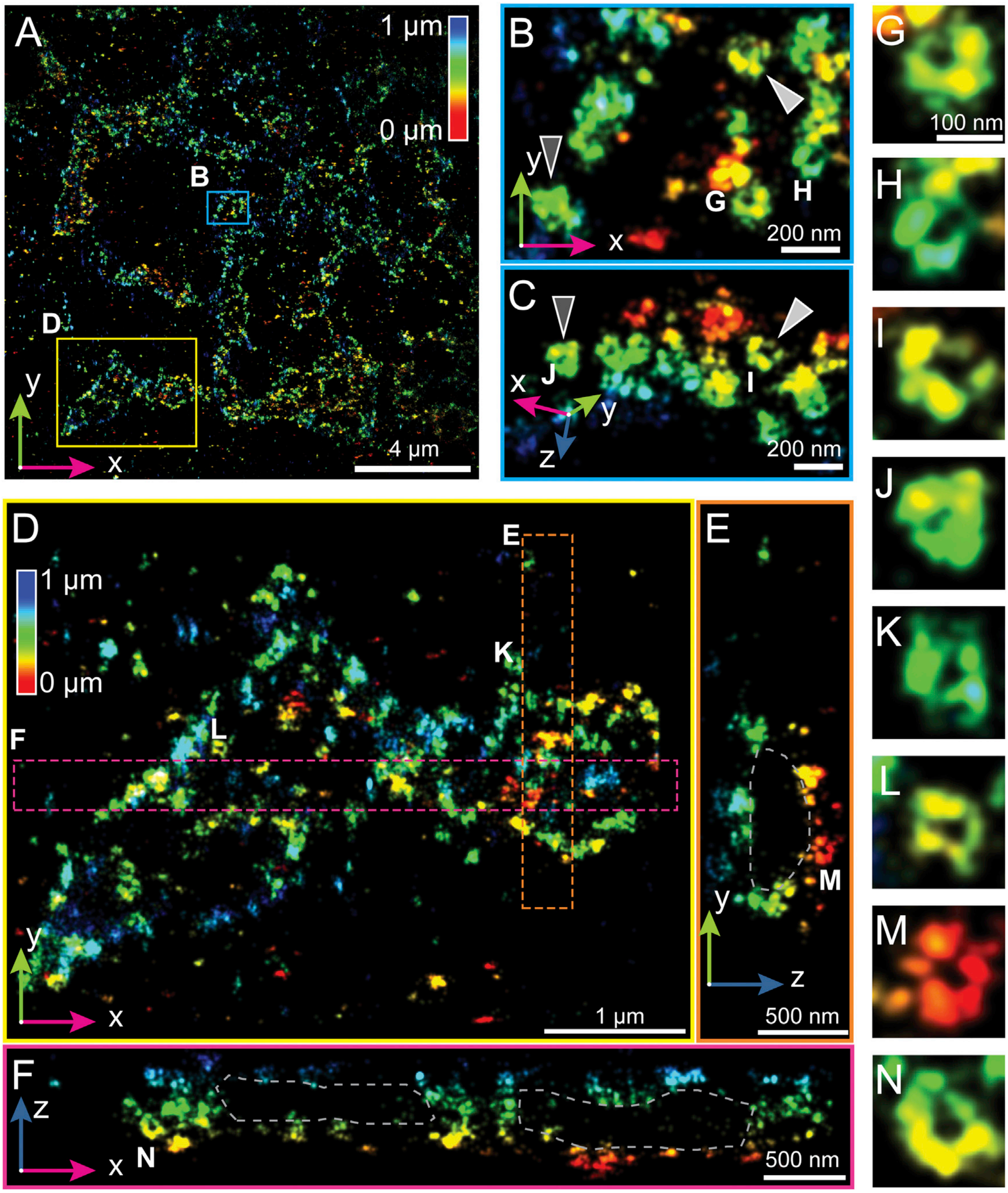

Figure 5. W-4PiSMSN Resolves Individual COPI-Coated Vesicles

COPI complexes were immunolabeled with an antibody against β′ COP and imaged with Alexa Fluor 647 in BSC-1 cells.

(A) Overview of a region of the field of view, with axial location of molecules color coded.

(B and C) Top (B) and side (C) view of the blue-boxed subregion indicated in (A) showing that COPI often forms round and hollow sphere-like structures. Dark gray and light gray arrowheads indicate the same COPI structures.

(D) x-y view of the area devoid of COPI as indicated by the yellow box in (A).

(E and F) x-z and y-z view of the orange (E) and magenta (F) boxed regions shown in (D) show that COPI surrounds an area presumably containing the Golgi cisternae.

(G–N) COPI vesicle structures as indicated by the respective labels in (B)–(F) shown at the same enlarged scale reveal circular structures.

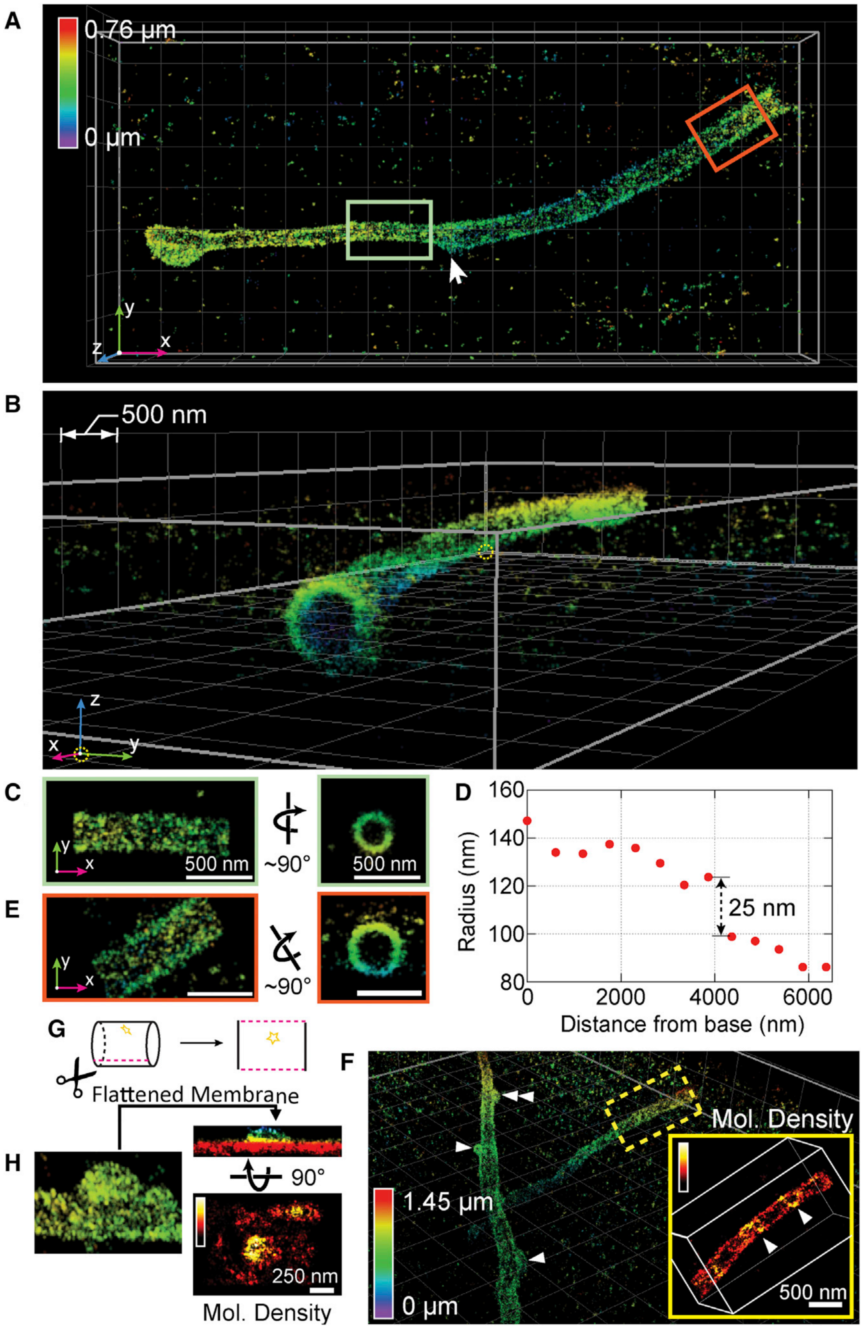

Figure 6. GPCR Smoothened on a Primary Cilium

(A and B) Top (A) and side (B) views of a primary cilium on an hTERT-RPE1 cell expressing pH-SMO, which was immunolabeled with Alexa Fluor 647.

(C and E) Views of sections close to the tip (C) and the base (E) as indicated by the light green and orange boxes in (A) show the localization of pH-SMO to the cilium membrane.

(D) Radius of different sections of the cilium as a function of their distance from the base.

(F) Overview of a cilium in another region of the sample, showing vesicle-like buds on the ciliary membrane surface (arrowheads). The inset shows the local density of the boxed region, which suggests a helical stripe organization of pH-SMO (arrowheads in inset).

(G and H) A bud-like profile shown in (F) can be unwrapped as depicted in (G), showing the height of the vesicle above the cilium membrane and the high molecular density of pH-SMO at the bud (H).

architectural elements of the nucleus, many of which are lost in commonly used spread chromatin preparations.

DISCUSSION

Through a confluence of several technological innovations, we have demonstrated that W-4PiSMSN provides unprecedented access to the ultrastructure of cells with ~10- to 20-nm isotropic resolution throughout their entire volume. This resolution is 20–50 times higher than conventional microscopy with imaging depth improved ~10-fold over state-of-the-art iPALM and 4Pi-SMSN. This development extends the application range of 4Pi-based SMSN dramatically: imaging is no longer limited to features within small sub-volumes of cells. Instead, we are capable of imaging organelles that span large volumes, exemplified by the mitochondrial network, the nuclear envelope, and synaptonemal complexes, which we capture in virtual entirety. Thus, W-4PiSMSN is a versatile and powerful tool that promises a new perspective on how proteins distribute across entire organelles throughout whole cells, a key unmet challenge in cell biology.

Is there room to further increase the spatial resolution of SMS nanoscopy? First, SMS resolution depends on the precision with which one can localize blinking molecules. The precision is approximately proportional to the sharpness of the PSF and, for negligible background noise, is inversely proportional to the square root of the number of detected photons. Our approach has focused mainly on creating the sharpest PSF and detecting as much of the emitted fluorescence light as possible. Recently, there have been promising developments that increase the number of emitted photons per molecule (Klehs et al., 2014; Ong et al., 2015; Vaughan et al., 2012), which we have not exploited here. Unfortunately, these advances have so far come at the expense of an increase in recording time. We anticipate, however, that with new generations of fluorophores or refined imaging buffers, these approaches can further improve image quality.

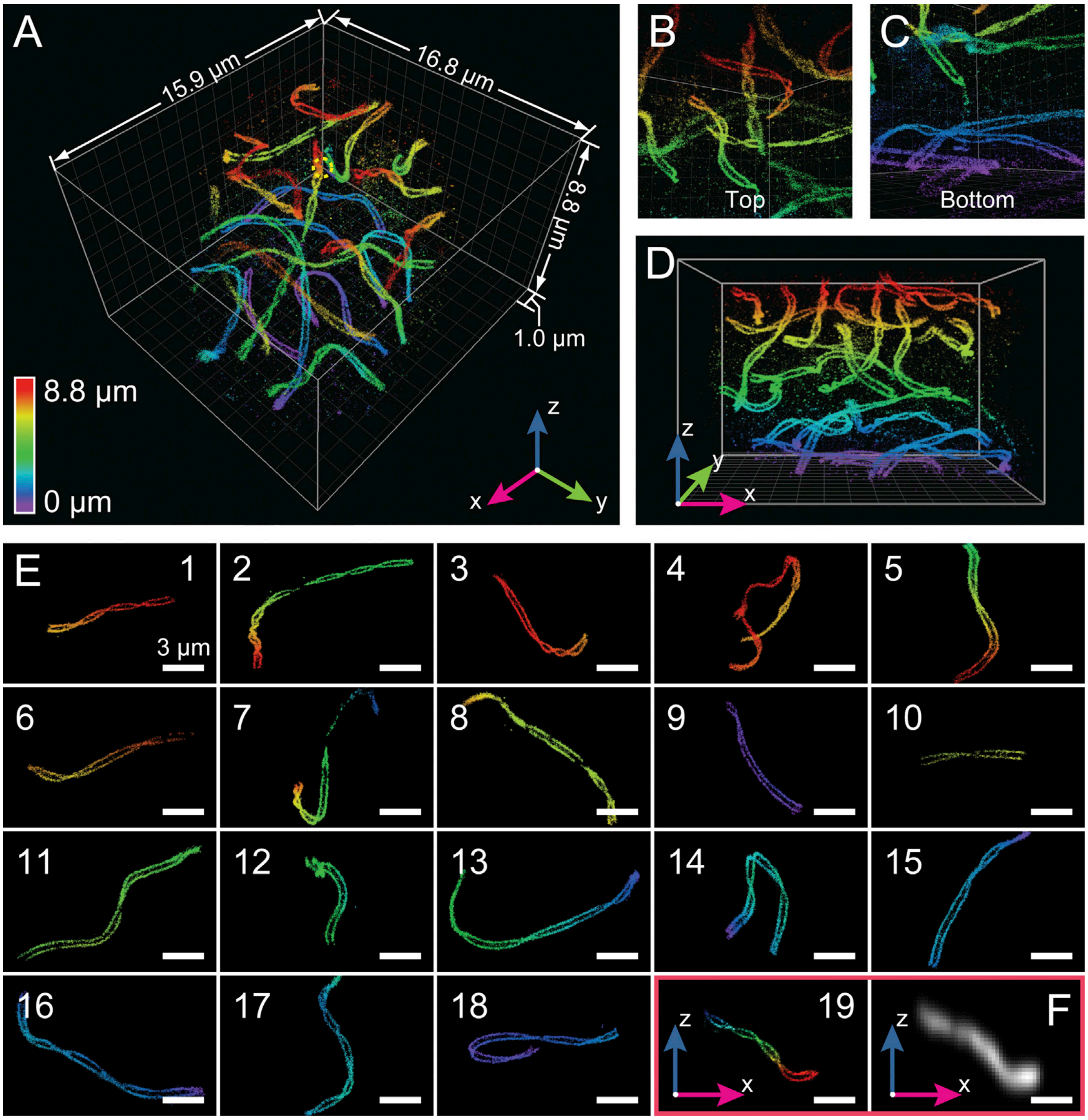

Figure 7. W-4PiSMSN of the Synaptonemal Complexes in a Whole-Mouse Spermatocyte

(A) Overview reconstructed from 21 optical sections. Lateral elements of the synaptonemal complex, spaced ~200 nm apart, are resolved throughout the ~9-µm depth of the spermatocyte at uniform resolution.

(B and C) Different views from locations inside the spermatocyte centered on top (B) and bottom (C) regions of the dataset.

(D) x-z view of (A).

(E) The 19 synaptonemal complexes from an entire mouse spermatocyte haploid genome were computationally isolated using a Euclidian distance-based clustering algorithm.

(F) A conventional image of the 19th synaptonemal complex in x-z view.

Scale bars in (E-19) and (F), 2 µm.

Second, image quality, or spatial resolution, of SMSN images depends on the density of localized molecules (Patterson et al., 2010; Shroff et al., 2008). The application examples we presented demonstrate that small features such as cylinder-shaped immunolabeled microtubules (~40-nm diameter) or ER tubules (~60-nm diameter) can now be easily resolved in 3D using light microscopy. This image interpretation is aided by the fact that the observer fills the gaps in the distribution of molecules along a tubule by mentally extrapolating from the expected tubular structure. We utilized several computational image processing techniques, including particle averaging of the bacteriophage data (Figure 1), clustering of the synaptonemal complexes (Figure 7; Movie S6), and "unwrapping" of cilia (Figure 6), to reconstruct complex structures. These approaches, which add constraints to data interpretation (e.g., the fact that cilia are tubular) and can be tailored to the cell biological question at

hand, allow us to extract structural details, which are more subtle than the labeling density suggests. Ultimately, labeling density is limited by the density of probe targets, usually proteins. The development of new labeling approaches that allow membrane targeting (Erdmann et al., 2014) overcome this restriction and additionally offer the possibility of revealing the membrane boundaries of individual organelles. Complementary approaches utilizing stochastic transient chemical binding can further address the limited pool of fluorescent labels and theoretically allow unlimited numbers of localized molecules (Giannone et al., 2010; Jungmann et al., 2014; Lew et al., 2011; Sharonov and Hochstrasser, 2006). However, even with the use of conventional labeling techniques, W-4PiSMSN is capable of visualizing otherwise inaccessible structures in a multitude of settings as demonstrated by the large range of presented applications.

In conclusion, we believe that the development of W-4PiSMSN represents the culmination of more than a decade's research on high-resolution fluorescence imaging techniques and establishes 3D biological imaging with molecular specificity and resolution in the 10-nm range as a general imaging technique.

EXPERIMENTAL PROCEDURES

Microscope Setup

A detailed description is provided as Supplemental Information. In brief, the 4Pi cavity of the system was set up vertically around two opposing high-NA objective lenses (Movie S7). Excitation light from three laser lines at wavelengths of 642, 561, and 405 nm was coupled into the upper objective for wide-field illumination. Following the concept by Aquino et al. (2011), fluorescence was collected by both objectives and passed through quarter wave plates, which enforced equal fractions of *s* and *p*-polarized light independent of the dipole emitter orientation. A custom Babinet-Soleil compensator corrected for dispersion and allowed adjusting the phase delay between the upper and lower interferometer arm independently for the two polarization components before the light was combined at a 50/50 beam splitter cube. We added deformable mirrors (Boston Micromachines, Multi-5.5) in planes conjugate to the back pupils of the objectives, which allowed for aberration correction, optimization of the PSF and introduction of astigmatism for artifact-free 3D localization. *s* and *p*-polarized fluorescence exiting the beam splitter cube at two sides was split with a polarizing beam splitter cube into four images featuring different interference phases. The four images were recorded simultaneously by a scientific complementary metal-oxide semiconductor (sCMOS) camera (Hamamatsu, ORCA-Flash 4.0v2). The entire system was controlled by a custom-written program in LabVIEW.

Aberration Correction Using Deformable Mirrors

Two deformable mirrors were independently adjusted as follows. For each interfering arm, starting from the flat voltage map (provided by the manufacturer), 28 theoretically generated membrane modes were applied sequentially with ten different voltage amplitudes per mode. The detected peak signals (0th moment Gaussian weighted sum) of a single fluorescent bead in focus were extracted for all amplitudes of the applied modes. The optimal amplitude for each mode was determined as the value that gave the maximum signal level by fitting a quadratic function to the measurements. These newly obtained amplitudes were added to the flat voltage map and serve as a starting point of another iteration. We used five iterations to achieve optimal system aberration correction. Details are provided the Supplemental Information.

Biological Sample Preparation

A complete description of cell culture, fluorescence labeling, coverslip, and buffer preparation is included in sections 15 to 26 of the Supplemental Information.

In short, 25-mm-diameter coverslips were cleaned by sonication in 1 M KOH for 15 min before use. Fluorescent 100-nm-diameter crimson beads were attached to the coverslip surface using poly-L-lysine. Cultured mammalian cells were grown on coverslips for 24–48 hr before fixation. COS-7 cells were used for microtubule, ER, and mitochondria samples. RPE-hTERT cells were used for nuclear pore complex and cilia samples. BSC1 cells were used for COPI samples. For synaptonemal complex samples, spermatocytes were isolated from the testes of mice and settled on coverslips before being fixed. T7 bacteriophages were isolated from *E. coli* cultures and labeled with Alexa Fluor 647 NHS Ester before being placed on coverslips.

Microtubule, mitochondria, and ER samples were fixed with 3% paraformaldehyde (PFA) + 0.1% glutaraldehyde before antibody labeling. A saponin pre-extraction step preceded fixation when only microtubules were labeled. Nuclear Pore Complex samples were fixed in -20°C methanol. COPI and synaptonemal samples were fixed in 4% PFA.

Antibodies against endogenous proteins were used to label microtubules (anti-α-tubulin), mitochondria (anti-TOM20), nuclear pore complexes (anti-Nup358), COPI (anti-β' COP), and synaptonemal complexes (anti-SYCP3). Overexpressed proteins were labeled with antibodies in ER samples (mEmerald-Sec61β using anti-GFP) and Cilia samples (pHlourin-mSmoothened using anti-GFP).

All one-color samples were labeled with Alexa Fluor 647, either using a commercial secondary antibody or an NHS ester. Two-color microtubule and mitochondria samples were labeled with Alexa Flour 647 and Cy3B, respectively. Cy3B-labeled secondary antibodies were made by conjugating reactive Cy3B with unlabeled antibodies. After secondary antibody labeling, COS-7 and BSC1 cell samples were post-fixed with 3% PFA + 0.1% GA.

Labeled biological samples were placed in an aluminum sample frame and covered with a second cleaned coverslip. A thin spacer and imaging buffer was placed between the two coverslips. The coverslips were held in the sample frame using an addition-curing silicone. The imaging buffer was either conventional or COT containing thiol buffer (Supplemental Information). Samples were imaged immediately after the silicone solidified.

W-4PiSMSN Data Acquisition

Four phase images are arranged along the splitting line of the sCMOS camera's upper and lower readout region. 50,000 to 320,000 camera frames were recorded at 50 or 100 fps, resulting in 10 min to 1.5 hr total acquisition time per dataset. For sample volumes thinner than 1.2 μm, the sample stage was not translated during data acquisition. For thicker samples, the stage was moved axially in 500-nm steps every 1,000–3,000 frames until the whole targeted imaging volume was covered, resulting in up to 21 z-steps and imaging volume depths of up to 9 μm. This axial scan was automatically repeated six to 19 times, and the data from the scans were combined. The laser intensity was manually adjusted during each experiment to optimize the emitter density per frame and to maximize detectable emissions starting at intensities as high as 35 kW/cm^2 to transfer emitters efficiently into dark states and decreasing to typically 5 kW/cm^2 near the end of the acquisition when the pool of blinking molecules had declined (Lin et al., 2015). Additionally, the laser intensity of the 405-nm laser was manually controlled over the course of imaging to optimize the active emitter density.

Single-Molecule Interference Phase Estimation

Raw camera frames were first isolated into four different phase images. The four phase images were then merged into a single image using a transformation matrix obtained from a combination of algorithms using log-polar and affine transformations (Supplemental Information). Single-molecule candidates from the merged frames were isolated and fitted with an elliptical Gaussian model using a maximum likelihood estimator accounting for the camera-specific noise associated with sCMOS cameras (Huang et al., 2013) (Supplemental Information). Estimates of single-molecule positions, width, total number of detected photons, background, and log-likelihood ratio were obtained. Intensities of the 0th moment Gaussian (Supplemental Information) were calculated by a weighted least-square fitting of a Gaussian with the amplitude being the only fitting parameter and weighted to take the sCMOS noise into account (Huang et al., 2013). Subsequently, the reduced moments of each polarization were extracted using a previously described approach

(Aquino et al., 2011). For two-color imaging, phase shifts between *s* and *p* polarization for the two color channels differ by 0.3 radians. These phase shift differences were measured independently from images of fluorescent beads recorded in two detection channels. With the obtained phase differences between the *s* and *p* polarization, the phase shifts between the four different phase images are known. Subsequently, the interference phases of the detected molecules were obtained by solving a set of equations describing the 0th moment intensities in *s* and *p* polarization channels as functions of an unknown offset and their relative phase shifts as characterized above (Supplemental Information).

Axial Position Analysis with Ridge-Finding Algorithm

We developed a metric *m*: $(\sigma_x^3/\sigma_y) - (\sigma_y^3/\sigma_x)$, where σ_x and σ_y are the estimated SDs of the 2D Gaussian for a single-molecule emission pattern. The metric preserves its monotonicity in the presence of aberrations, and we used it to estimate an unambiguous (but still rough) position of each single emitter, before the phase estimate was used to pinpoint the exact axial position of a molecule.

For every 3,000–5,000 camera frames, we generated a 2D histogram image from phase estimations and the metric *m* from all detected single molecules. As *m* is monotonic and the single-molecule phase is periodic with a period of 2π, the resulting 2D histogram looks like a pattern of tilted repeating stripes (Figure S3). We developed a ridge-finding algorithm to determine a series of connected vectors defining the correspondence between the determined phase values and the values of *m*. This allowed us to unwrap the periodic phase for unambiguous axial localization. A detailed description of the algorithm is provided in the Supplemental Information.

Drift Correction

3D drift correction was performed by first calculating the distance pairs between image segments (3,000–5,000 frames), and subsequently forming three sets of equations for x, y, and z, respectively (Li et al., 2013; Wang et al., 2014). A least-square solution that minimizes the overall error of the set of equations was obtained and back-substituted into all equations. Errors can be calculated from each of these equations and a specific equation within the set is removed from the stack if its error is larger than 7 nm. This process was repeated until no error was larger than 7 nm or the matrix was no longer at its full rank. This allowed us to correct system and sample induced drift in 3D with short segments of data (Supplemental Information).

Optical Segment Alignment

To image thick samples, optical sections were recorded at different axial positions of the sample by axially translating the z-piezo holding the sample stage. The localization data contain x, y, and z position estimates of different optical sections and must be aligned/stitched seamlessly to support the high precision obtained in W-4PiSMSN. Previous methods (Huang et al., 2008) that shifted each optical section by a constant in the axial direction have been prone to introduce misalignment of the optical sections and subsequently deteriorate the resolution achievable in thick samples. Here, we developed an optical alignment method based on 3D cross-correlation. In the W-4PiSMSN system, optical sections are ~1.2 μm thick. Whole-cell samples were scanned in the axial direction with 500-nm step sizes. This allowed for abundant overlapping regions between adjacent optical sections and provided critical information for precise optical section alignment using the developed 3D cross-correlation methods as described in Supplemental Information.

SUPPLEMENTAL INFORMATION

Supplemental Information includes Supplemental Experimental Procedures, seven figures, and seven movies and can be found with this article online at http://dx.doi.org/10.1016/j.cell.2016.06.016.

AUTHOR CONTRIBUTIONS

F.H., G.S., E.S.A., M.J.B., and J.B. designed the microscope. F.H., G.S., E.S.A., T.P., M.J.B., and J.B. built the instrument and developed the computer code. F.H., G.S., E.S.A., L.K.S., W.C.D., E.B.K., Y.Z., and J.B. optimized and tested the microscope. L.K.S., W.C.D., F.E.R.-M., J.R.M., I.I., M.L., M.A.H., C.J.-W., C.P.L., J.E.R., D.T., and J.B. designed biological experiments. F.H., G.S., L.K.S., W.C.D., F.E.R.-M., J.R.M., I.I., and M.L. optimized sample preparation protocols and prepared samples. F.H., G.S., E.S.A., L.K.S., E.B.K., T.P., and J.B. visualized the data. All authors contributed to writing the manuscript.

ACKNOWLEDGMENTS

We would like to thank Travis Gould (Bates College) for initial discussion of the instrument design, Hideo Takakura, Hoong Chuin Lim, Stephanie Baguley (Yale University), and Yasuhiro Fujiwara (The Jackson Laboratory) for help in sample preparation, and Wen Jiang (Purdue University) for providing purified T7 phage samples. We thank Elias Coutavas and Günter Blobel (Rockefeller University) for the Nup358 antibody. We thank Mike Mosbacher and Christopher Jost for help on early CAD design and thermal simulations of the instrument, and Markus Schwarzhuber, Florian Schroeder, Stefan Politzka, Tobias Wagner, and Robert Muth for help on mechanical part design, custom electronics, sample chamber optimization, and data visualization. We thank George Takahashi from the Purdue Envision Center for help on data visualization. We thank Michael Mlodzianoski for discussions on the manuscript. This work was primarily supported by a grant from the Wellcome Trust (095927/A/11/Z). F.H. received funding from a James Hudson Brown–Alexander Brown Coxe Postdoctoral Fellowship. E.B.K. was supported by The Denmark-America Foundation (Coloplast), Civilingeniør Frants Allings Legat, Knud Højgaards Fond, Reinholdt W. Jorcks Fond, Berg Nielsens Legat, and Ingeniør Alexandre Haynman og Hustru Nina Haynmans Fond. G.S. and E.S.A. were supported by the Wellcome Trust (092096) and Cancer Research UK (C6946/A14492). F.E.R-M. and D.T. were supported by a grant from the NIH (R21 HD078851-01). I.I. was supported by a grant from the NIH (R01 GM065835) to C.J.-W. C.J.-W. is an investigator of the Howard Hughes Medical Institute. M.A.H was supported by a grant from NIH (P01 GM99640). J.R.M. and C.P.L. were supported by grants from the NIH (R01 GM105672 and R01 HL124402). J.B. declares significant financial interest in Bruker, which manufactures SMSN instruments. F.H. and J.B. have filed a patent application for sCMOS-related data analysis (WO 2014144443 A2). The invention and software is licensed to Hamamatsu Photonics K.K.

Received: February 29, 2016
Revised: May 2, 2016
Accepted: June 3, 2016
Published: July 7, 2016

REFERENCES

Aquino, D., Schönle, A., Geisler, C., Middendorff, C.V., Wurm, C.A., Okamura, Y., Lang, T., Hell, S.W., and Egner, A. (2011). Two-color nanoscopy of three-dimensional volumes by 4Pi detection of stochastically switched fluorophores. Nat. Methods *8*, 353–359.

Betzig, E., Patterson, G.H., Sougrat, R., Lindwasser, O.W., Olenych, S., Bonifacino, J.S., Davidson, M.W., Lippincott-Schwartz, J., and Hess, H.F. (2006). Imaging intracellular fluorescent proteins at nanometer resolution. Science *313*, 1642–1645.

Bewersdorf, J., Schmidt, R., and Hell, S.W. (2006). Comparison of I5M and 4Pi-microscopy. J. Microsc. *222*, 105–117.

Briggs, J.A.G. (2013). Structural biology in situ–the potential of subtomogram averaging. Curr. Opin. Struct. Biol. *23*, 261–267.

Broeken, J., Johnson, H., Lidke, D.S., Liu, S., Nieuwenhuizen, R.P.J., Stallinga, S., Lidke, K.A., and Rieger, B. (2015). Resolution improvement by 3D particle averaging in localization microscopy. Methods Appl. Fluoresc. *3*, 014003.

Brown, T.A., Tkachuk, A.N., Shtengel, G., Kopek, B.G., Bogenhagen, D.F., Hess, H.F., and Clayton, D.A. (2011). Superresolution fluorescence imaging of mitochondrial nucleoids reveals their spatial range, limits, and membrane interaction. Mol. Cell. Biol. *31*, 4994–5010.

Burke, D., Patton, B., Huang, F., Bewersdorf, J., and Booth, M.J. (2015). Adaptive optics correction of specimen-induced aberrations in single-molecule switching microscopy. Optica *2*, 177.

Carlton, P.M. (2008). Three-dimensional structured illumination microscopy and its application to chromosome structure. Chromosome Res. *16*, 351–365.

Case, L.B., Baird, M.A., Shtengel, G., Campbell, S.L., Hess, H.F., Davidson, M.W., and Waterman, C.M. (2015). Molecular mechanism of vinculin activation and nanoscale spatial organization in focal adhesions. Nat. Cell Biol. *17*, 880–892.

Chojnacki, J., Staudt, T., Glass, B., Bingen, P., Engelhardt, J., Anders, M., Schneider, J., Müller, B., Hell, S.W., and Kräusslich, H.-G. (2012). Maturation-dependent HIV-1 surface protein redistribution revealed by fluorescence nanoscopy. Science *338*, 524–528.

Erdmann, R.S., Takakura, H., Thompson, A.D., Rivera-Molina, F., Allgeyer, E.S., Bewersdorf, J., Toomre, D., and Schepartz, A. (2014). Super-resolution imaging of the Golgi in live cells with a bioorthogonal ceramide probe. Angew. Chem. Int. Ed. Engl. *53*, 10242–10246.

Frey, T.G., and Mannella, C.A. (2000). The internal structure of mitochondria. Trends Biochem. Sci. *25*, 319–324.

Fritsche, M., Reinholdt, L.G., Lessard, M., Handel, M.A., Bewersdorf, J., and Heermann, D.W. (2012). The impact of entropy on the spatial organization of synaptonemal complexes within the cell nucleus. PLoS ONE *7*, e36282.

Giannone, G., Hosy, E., Levet, F., Constals, A., Schulze, K., Sobolevsky, A.I., Rosconi, M.P., Gouaux, E., Tampé, R., Choquet, D., and Cognet, L. (2010). Dynamic superresolution imaging of endogenous proteins on living cells at ultra-high density. Biophys. J. *99*, 1303–1310.

Gould, T.J., Hess, S.T., and Bewersdorf, J. (2012a). Optical nanoscopy: from acquisition to analysis. Annu. Rev. Biomed. Eng. *14*, 231–254.

Gould, T.J., Burke, D., Bewersdorf, J., and Booth, M.J. (2012b). Adaptive optics enables 3D STED microscopy in aberrating specimens. Opt. Express *20*, 20998–21009.

He, M., Subramanian, R., Bangs, F., Omelchenko, T., Liem, K.F., Jr., Kapoor, T.M., and Anderson, K.V. (2014). The kinesin-4 protein Kif7 regulates mammalian Hedgehog signalling by organizing the cilium tip compartment. Nat. Cell Biol. *16*, 663–672.

Hell, S.W. (2007). Far-field optical nanoscopy. Science *316*, 1153–1158.

Hell, S.W., and Wichmann, J. (1994). Breaking the diffraction resolution limit by stimulated emission: stimulated-emission-depletion fluorescence microscopy. Opt. Lett. *19*, 780–782.

Hess, S.T., Girirajan, T.P.K., and Mason, M.D. (2006). Ultra-high resolution imaging by fluorescence photoactivation localization microscopy. Biophys. J. *91*, 4258–4272.

Hu, B., Margolin, W., Molineux, I.J., and Liu, J. (2013). The bacteriophage t7 virion undergoes extensive structural remodeling during infection. Science *339*, 576–579.

Huang, B., Jones, S.A., Brandenburg, B., and Zhuang, X. (2008). Whole-cell 3D STORM reveals interactions between cellular structures with nanometer-scale resolution. Nat. Methods *5*, 1047–1052.

Huang, F., Hartwich, T.M.P., Rivera-Molina, F.E., Lin, Y., Duim, W.C., Long, J.J., Uchil, P.D., Myers, J.R., Baird, M.A., Mothes, W., et al. (2013). Video-rate nanoscopy using sCMOS camera-specific single-molecule localization algorithms. Nat. Methods *10*, 653–658.

Juette, M.F., Gould, T.J., Lessard, M.D., Mlodzianoski, M.J., Nagpure, B.S., Bennett, B.T., Hess, S.T., and Bewersdorf, J. (2008). Three-dimensional sub-100 nm resolution fluorescence microscopy of thick samples. Nat. Methods *5*, 527–529.

Jungmann, R., Avendaño, M.S., Woehrstein, J.B., Dai, M., Shih, W.M., and Yin, P. (2014). Multiplexed 3D cellular super-resolution imaging with DNA-PAINT and Exchange-PAINT. Nat. Methods *11*, 313–318.

Kanchanawong, P., Shtengel, G., Pasapera, A.M., Ramko, E.B., Davidson, M.W., Hess, H.F., and Waterman, C.M. (2010). Nanoscale architecture of integrin-based cell adhesions. Nature *468*, 580–584.

Klehs, K., Spahn, C., Endesfelder, U., Lee, S.F., Fürstenberg, A., and Heilemann, M. (2014). Increasing the brightness of cyanine fluorophores for single-molecule and superresolution imaging. ChemPhysChem *15*, 637–641.

Lew, M.D., Lee, S.F., Ptacin, J.L., Lee, M.K., Twieg, R.J., Shapiro, L., and Moerner, W.E. (2011). Three-dimensional superresolution colocalization of intracellular protein superstructures and the cell surface in live Caulobacter crescentus. Proc. Natl. Acad. Sci. USA *108*, E1102–E1110.

Li, X., Mooney, P., Zheng, S., Booth, C.R., Braunfeld, M.B., Gubbens, S., Agard, D.A., and Cheng, Y. (2013). Electron counting and beam-induced motion correction enable near-atomic-resolution single-particle cryo-EM. Nat. Methods *10*, 584–590.

Lichtman, J.W., and Conchello, J.-A. (2005). Fluorescence microscopy. Nat. Methods *2*, 910–919.

Lin, Y., Long, J.J., Huang, F., Duim, W.C., Kirschbaum, S., Zhang, Y., Schroeder, L.K., Rebane, A.A., Velasco, M.G.M., Virrueta, A., et al. (2015). Quantifying and optimizing single-molecule switching nanoscopy at high speeds. PLoS ONE *10*, e0128135.

Liu, K.S.Y., Siebert, M., Mertel, S., Knoche, E., Wegener, S., Wichmann, C., Matkovic, T., Muhammad, K., Depner, H., Mettke, C., et al. (2011). RIM-binding protein, a central part of the active zone, is essential for neurotransmitter release. Science *334*, 1565–1569.

Liu, S., Kromann, E.B., Krueger, W.D., Bewersdorf, J., and Lidke, K.A. (2013). Three dimensional single molecule localization using a phase retrieved pupil function. Opt. Express *21*, 29462–29487.

McGorty, R., Schnitzbauer, J., Zhang, W., and Huang, B. (2014). Correction of depth-dependent aberrations in 3D single-molecule localization and super-resolution microscopy. Opt. Lett. *39*, 275–278.

Mlodzianoski, M.J., Schreiner, J.M., Callahan, S.P., Smolková, K., Dlasková, A., Santorová, J., Ježek, P., and Bewersdorf, J. (2011). Sample drift correction in 3D fluorescence photoactivation localization microscopy. Opt. Express *19*, 15009–15019.

Nieuwenhuizen, R.P.J., Lidke, K.A., Bates, M., Puig, D.L., Grünwald, D., Stallinga, S., and Rieger, B. (2013). Measuring image resolution in optical nanoscopy. Nat. Methods *10*, 557–562.

Ong, W.Q., Citron, Y.R., Schnitzbauer, J., Kamiyama, D., and Huang, B. (2015). Heavy water: a simple solution to increasing the brightness of fluorescent proteins in super-resolution imaging. Chem. Commun. (Camb.) *51*, 13451–13453.

Orci, L., Stamnes, M., Ravazzola, M., Amherdt, M., Perrelet, A., Söllner, T.H., and Rothman, J.E. (1997). Bidirectional transport by distinct populations of COPI-coated vesicles. Cell *90*, 335–349.

Page, S.L., and Hawley, R.S. (2004). The genetics and molecular biology of the synaptonemal complex. Annu. Rev. Cell Dev. Biol. *20*, 525–558.

Patterson, G., Davidson, M., Manley, S., and Lippincott-Schwartz, J. (2010). Superresolution imaging using single-molecule localization. Annu. Rev. Phys. Chem. *61*, 345–367.

Pavani, S.R.P., Thompson, M.A., Biteen, J.S., Lord, S.J., Liu, N., Twieg, R.J., Piestun, R., and Moerner, W.E. (2009). Three-dimensional, single-molecule fluorescence imaging beyond the diffraction limit by using a double-helix point spread function. Proc. Natl. Acad. Sci. USA *106*, 2995–2999.

Pellett, P.A., Dietrich, F., Bewersdorf, J., Rothman, J.E., and Lavieu, G. (2013). Inter-Golgi transport mediated by COPI-containing vesicles carrying small cargoes. eLife *2*, e01296.

Qiao, H., Chen, J.K., Reynolds, A., Höög, C., Paddy, M., and Hunter, N. (2012). Interplay between synaptonemal complex, homologous recombination, and centromeres during mammalian meiosis. PLoS Genet. *8*, e1002790.

Ram, S., Prabhat, P., Chao, J., Ward, E.S., and Ober, R.J. (2008). High accuracy 3D quantum dot tracking with multifocal plane microscopy for the study of fast intracellular dynamics in live cells. Biophys. J. *95*, 6025–6043.

Rust, M.J., Bates, M., and Zhuang, X. (2006). Sub-diffraction-limit imaging by stochastic optical reconstruction microscopy (STORM). Nat. Methods *3*, 793–795.

Schücker, K., Holm, T., Franke, C., Sauer, M., and Benavente, R. (2015). Elucidation of synaptonemal complex organization by super-resolution imaging with isotropic resolution. Proc. Natl. Acad. Sci. USA *112*, 2029–2033.

Sharonov, A., and Hochstrasser, R.M. (2006). Wide-field subdiffraction imaging by accumulated binding of diffusing probes. Proc. Natl. Acad. Sci. USA *103*, 18911–18916.

Shroff, H., Galbraith, C.G., Galbraith, J.A., and Betzig, E. (2008). Live-cell photoactivated localization microscopy of nanoscale adhesion dynamics. Nat. Methods *5*, 417–423.

Shtengel, G., Galbraith, J.A., Galbraith, C.G., Lippincott-Schwartz, J., Gillette, J.M., Manley, S., Sougrat, R., Waterman, C.M., Kanchanawong, P., Davidson, M.W., et al. (2009). Interferometric fluorescent super-resolution microscopy resolves 3D cellular ultrastructure. Proc. Natl. Acad. Sci. USA *106*, 3125–3130.

Van Engelenburg, S.B., Shtengel, G., Sengupta, P., Waki, K., Jarnik, M., Ablan, S.D., Freed, E.O., Hess, H.F., and Lippincott-Schwartz, J. (2014). Distribution of ESCRT machinery at HIV assembly sites reveals virus scaffolding of ESCRT subunits. Science *343*, 653–656.

Vaughan, J.C., Jia, S., and Zhuang, X. (2012). Ultrabright photoactivatable fluorophores created by reductive caging. Nat. Methods *9*, 1181–1184.

von Appen, A., Kosinski, J., Sparks, L., Ori, A., DiGuilio, A.L., Vollmer, B., Mackmull, M.-T., Banterle, N., Parca, L., Kastritis, P., et al. (2015). In situ structural analysis of the human nuclear pore complex. Nature *526*, 140–143.

von Diezmann, A., Lee, M.Y., Lew, M.D., and Moerner, W.E. (2015). Correcting field-dependent aberrations with nanoscale accuracy in three-dimensional single-molecule localization microscopy. Optica *2*, 985–993.

von Middendorff, C., Egner, A., Geisler, C., Hell, S.W., and Schönle, A. (2008). Isotropic 3D Nanoscopy based on single emitter switching. Opt. Express *16*, 20774–20788.

Wang, W.-J., Tay, H.G., Soni, R., Perumal, G.S., Goll, M.G., Macaluso, F.P., Asara, J.M., Amack, J.D., and Tsou, M.-F.B. (2013). CEP162 is an axoneme-recognition protein promoting ciliary transition zone assembly at the cilia base. Nat. Cell Biol. *15*, 591–601.

Wang, Y., Schnitzbauer, J., Hu, Z., Li, X., Cheng, Y., Huang, Z.-L., and Huang, B. (2014). Localization events-based sample drift correction for localization microscopy with redundant cross-correlation algorithm. Opt. Express *22*, 15982–15991.

Wood, C.R., and Rosenbaum, J.L. (2015). Ciliary ectosomes: transmissions from the cell's antenna. Trends Cell Biol. *25*, 276–285.

Xu, K., Babcock, H.P., and Zhuang, X. (2012). Dual-objective STORM reveals three-dimensional filament organization in the actin cytoskeleton. Nat. Methods *9*, 185–188.

Xu, K., Zhong, G., and Zhuang, X. (2013). Actin, spectrin, and associated proteins form a periodic cytoskeletal structure in axons. Science *339*, 452–456.

Yang, T.T., Hampilos, P.J., Nathwani, B., Miller, C.H., Sutaria, N.D., and Liao, J.-C. (2013). Superresolution STED microscopy reveals differential localization in primary cilia. Cytoskeleton *70*, 54–65.

Yang, T.T., Su, J., Wang, W.-J., Craige, B., Witman, G.B., Tsou, M.F., and Liao, J.-C. (2015). Superresolution pattern recognition reveals the architectural map of the ciliary transition zone. Sci. Rep. *5*, 14096.

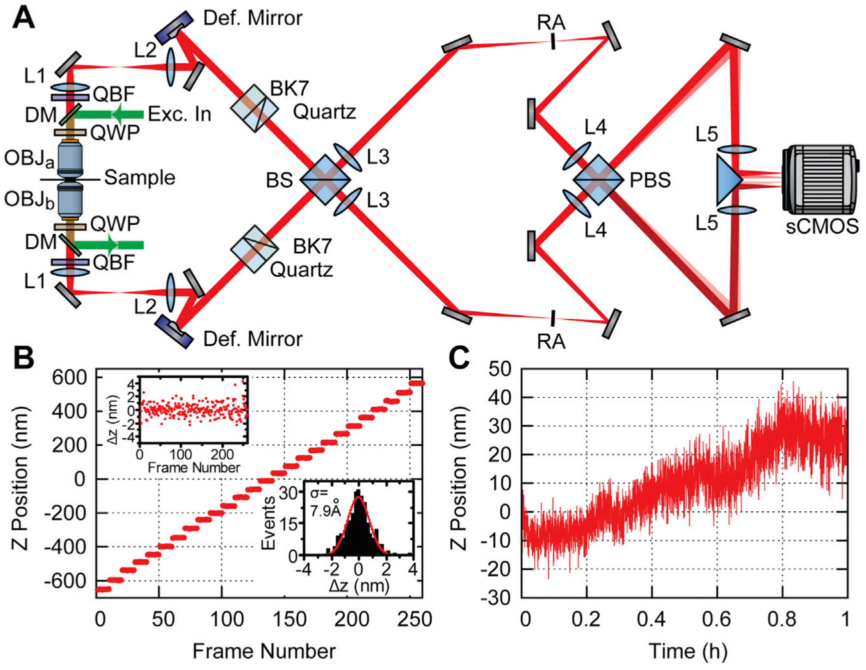

Figure S1. W-4PiSMSN Setup and System Characterization, Related to Figure 1

(A) Simplified system diagram of W-4PiSMSN.

(B) Localization results of W-4PiSMSN of a fluorescent bead imaged with 50 nm steps over an axial range of 1.2 μm. Inserts show residual errors displayed for each step and in a histogram.

(C) Instrumental drift along the axial direction over 1 hr.

L1-L5: Lenses, OBJ: Objective, QWP: Quarter-Wave Plate, DM: Dichroic Mirror, QBF: Quad-Band Bandpass Filter, Def. Mirror: Deformable Mirror, BS: Beam Splitter Cube, PBS: Polarizing Beam Splitter Cube, RA: Rectangular Aperture.

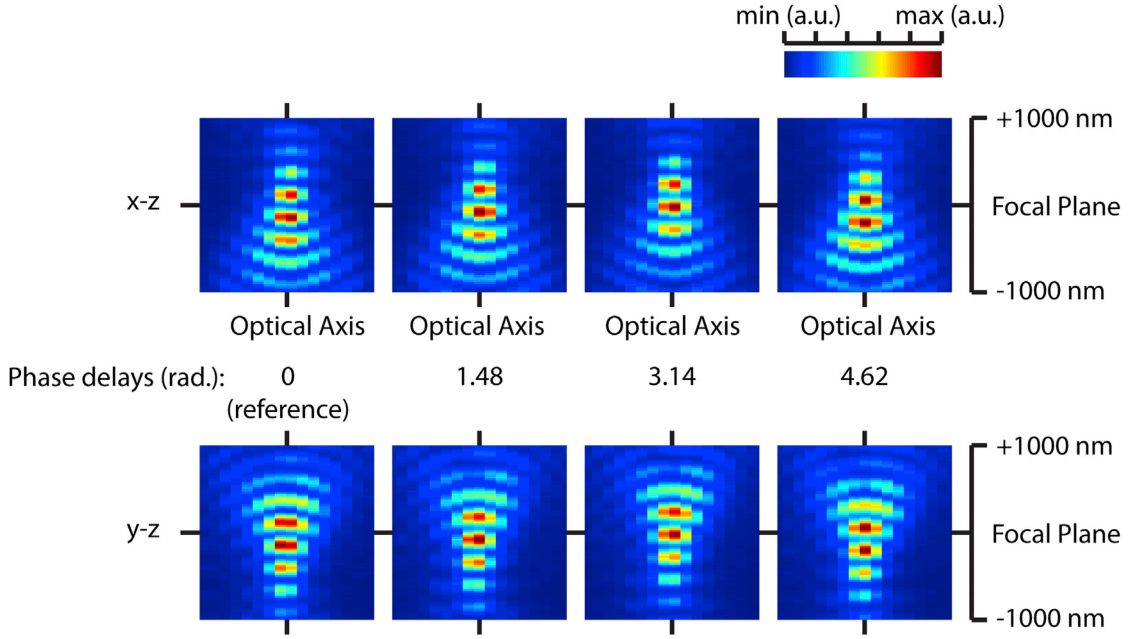

Figure S2. W-4PiSMSN Point Spread Function, Related to Figure 1

Central x-z and y-z sections of W-4PiSMSN point-spread functions in the four images recorded by the sCMOS camera demonstrating interference and astigmatism introduced by the coherent detection cavity and deformable mirrors.

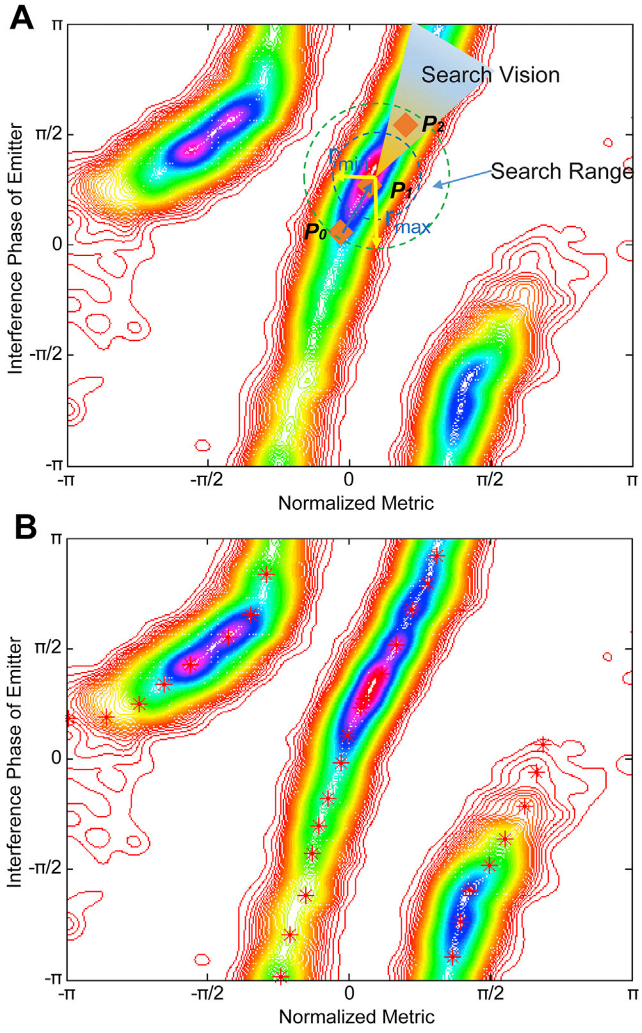

Figure S3. Concept of Ridge-Finding Algorithm, Related to Figure 1

(A) Ridge-finding algorithm concept including demonstrations of vision field, jump range, and directions of the current step. Contour plot of the 2D histogram generated from single-molecule interference phase values and normalized metric values.

(B) Identified monotonic ridge of metric versus phase curve before phase unwrapping (red stars).

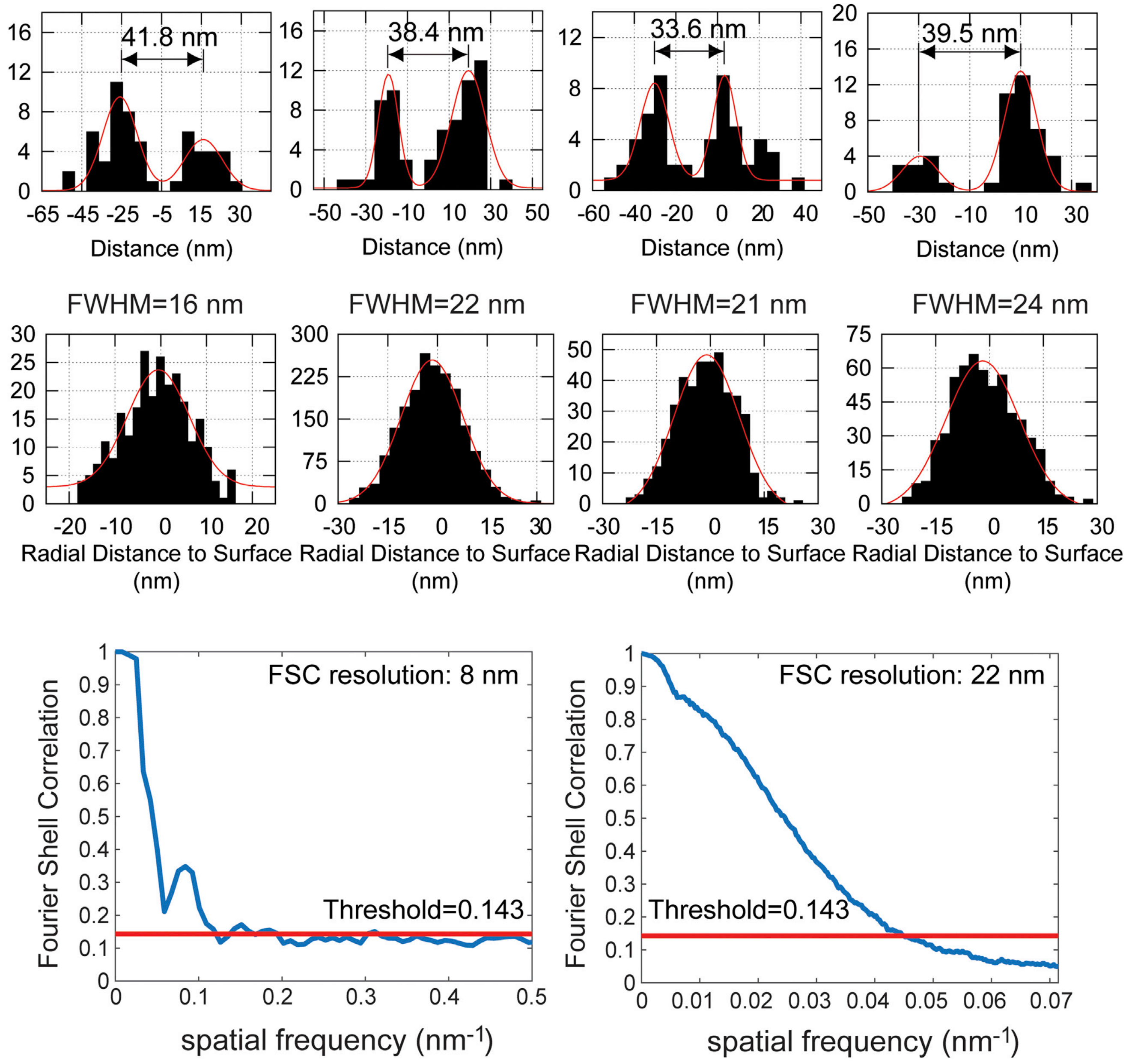

Figure S4. Line Profiles, Residue Plots, and Fourier Shell Correlation Resolution, Related to Figure 1

Top: four line profiles across x-y slices of microtubules shown in Figures 1D–1F. Middle: residual distances from single-molecule localizations to cylinder surface fit to four segments of the microtubule data. Bottom: Fourier shell correlation (FSC) measurement of resolution in a sub-region of ER data (right) (Figures 1B and 1C) and the combined phage data (left) (Figures 1I–1L) based on custom-written software extended to 3D from previously demonstrated Fourier ring correlation on SMSN datasets (Nieuwenhuizen et al., 2013).

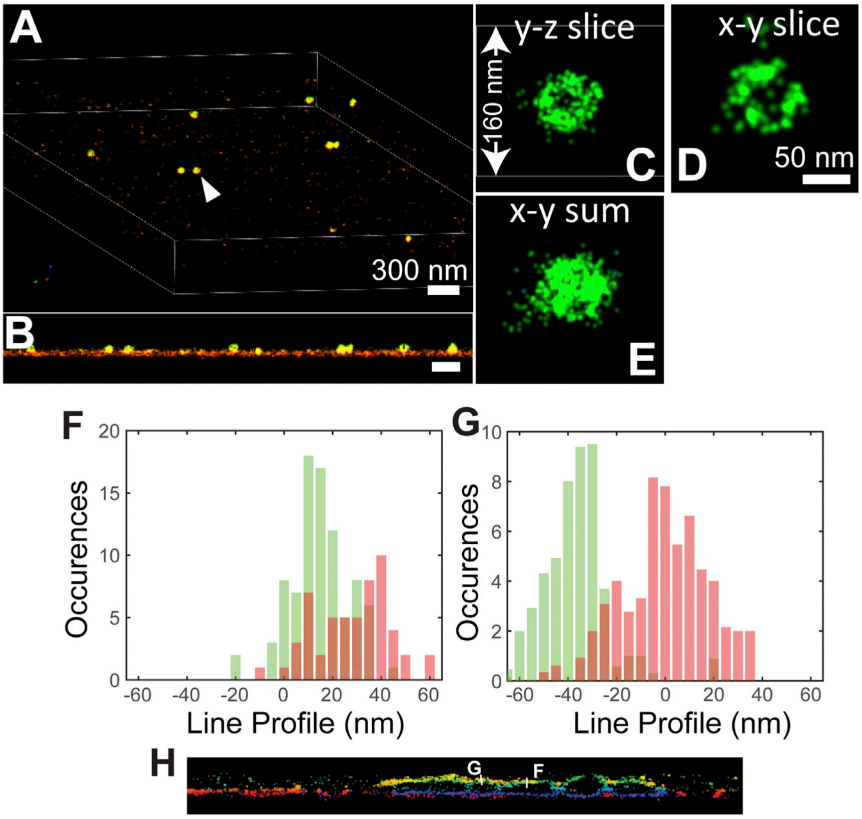

Figure S5. Overview of an Individual Phage and Examples of Line Profiles from Figure 2, Related to Figures 1 and 2

(A) Overview of Alexa Fluor 647 labeled phages imaged by W-4PiSMSN.

(B) x-z view of the entire sample showing coverslip surface and individual phages.

(C–E) Cross-sections and projection of an isolated phage (arrowhead in A).

(F and G) Examples of line profiles (integrated over a width of 200 nm) of the two-color image shown in Figure 2.

(H) Line profile positions in Figure 2C.

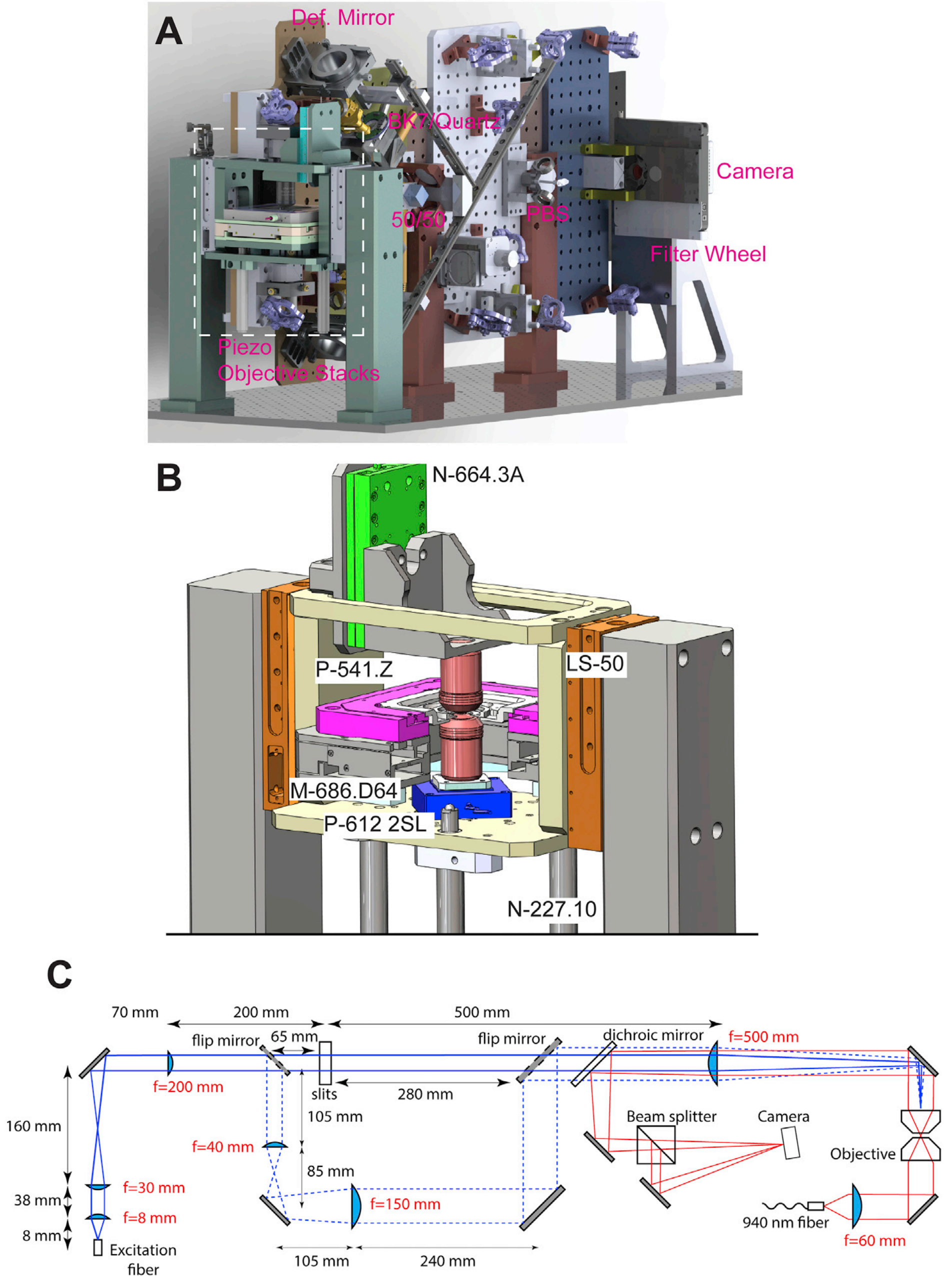

Figure S6. Instrument CAD Renderings and Layout of the Excitation and Diagnostic Beam, Related to Figure 1

(A and B) CAD renderings of the W-4PiSMSN instrument and the piezo-objective stack. 50/50: 50/50 beam splitter cube, PBS: polarizing beam splitter cube, Def. Mirror: deformable mirror. Please see Movie S7 for animation and more details.

(C) Excitation and diagnostic beam layout. The excitation light from a polarization-maintaining single-mode fiber (solid blue line) is first collimated by an aspheric lens (f = 8 mm) and further expanded ~6.6X to a size of ~12 mm. This beam passes through a pair of square apertures of ~5x5mm that cropped the center-most uniform part of the beam. An f = 500 mm lens focuses the cropped beam to the back focal plane of the top objective, uniformly illuminating an ~18x18 μm area in the focal plane. For overview, a pair of flip mirrors route the beam through an alternative path (dashed blue line) that bypasses the apertures. The overview beam is further expanded ~4X before being focused by the f = 500 mm lens to the back focal plane of the objective and illuminates a ~100-μm diameter area in the focal plane. To lock the relative position of the two objectives, the laser light from a 940 nm diode laser (red solid line) is collimated by a lens to overfill the back focal plane of the bottom objective, which focuses the light to a spot in the common focal plane. This focus is imaged by the top objective producing a collimated beam propagating in the opposite direction of the excitation light. The f = 500 mm lens focuses the beam through a biplane geometry to a camera.

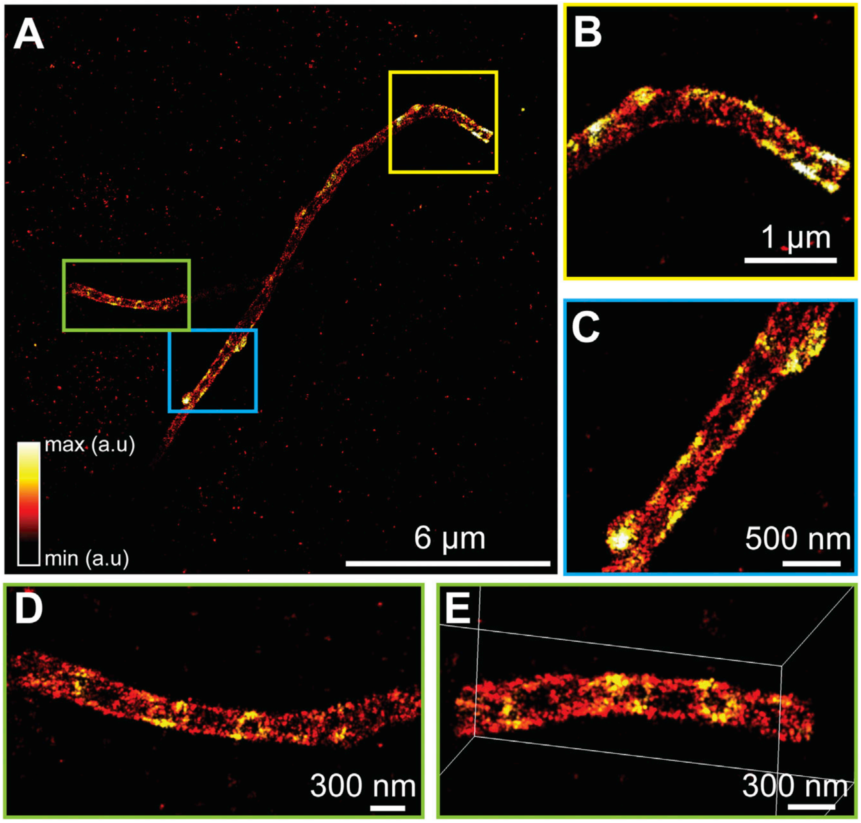

Figure S7. Molecular Density of the Cilia Membrane Protein GPCR Smoothened, Related to Figure 6

(A) Overview of a cilium, color-coded by molecular density. Density was calculated by counting the number of localizations surrounding each localization within a 100-nm radius.

(B–E) Zoomed and rotated views show increased molecular density at the base of the cilium (B), at positions with potential budding vesicles (C), and in bands along the length of the cilium (D and E) which suggest potential functional arrangements of SMO.

Cell, Volume *166*

Supplemental Information

Ultra-High Resolution 3D Imaging of Whole Cells

Fang Huang, George Sirinakis, Edward S. Allgeyer, Lena K. Schroeder, Whitney C. Duim, Emil B. Kromann, Thomy Phan, Felix E. Rivera-Molina, Jordan R. Myers, Irnov Irnov, Mark Lessard, Yongdeng Zhang, Mary Ann Handel, Christine Jacobs-Wagner, C. Patrick Lusk, James E. Rothman, Derek Toomre, Martin J. Booth, and Joerg Bewersdorf

Supplemental Materials

ULTRA-HIGH RESOLUTION 3D IMAGING OF WHOLE CELLS

Fang Huang[1,2,†], George Sirinakis[1,3†], Edward S. Allgeyer[1], Lena K. Schroeder[1], Whitney C. Duim[1,4], Emil B. Kromann[1,5], Thomy Phan[1], Felix E. Rivera-Molina[1], Jordan R. Myers[1], Irnov Irnov[6,7], Mark Lessard[8], Yongdeng Zhang[1], Mary Ann Handel[8], Christine Jacobs-Wagner[6,7,9,10], C. Patrick Lusk[1], James E. Rothman[1,11], Derek Toomre[1,11], Martin J. Booth[12,13], Joerg Bewersdorf[1,5,11,*]

1. Department of Cell Biology, School of Medicine, Yale University, New Haven, CT 06520, USA
2. Weldon School of Biomedical Engineering, Purdue University, West Lafayette, IN 47907, USA
3. The Gurdon Institute, University of Cambridge, CB2 1QN, UK
4. Department of Chemistry, Harvey Mudd College, Claremont, CA 91711, USA
5. Department of Biomedical Engineering, Yale University, CT 06520, USA
6. Microbial Sciences Institute, Yale University, West Haven, CT 06477, USA
7. Department of Molecular, Cellular and Developmental Biology, Yale University, New Haven, CT 06520, USA
8. The Jackson Laboratory, Bar Harbor, ME 04609, USA
9. Howard Hughes Medical Institute, Yale University, New Haven, CT 06520, USA
10. Department of Microbial Pathogenesis, Yale School of Medicine, New Haven, CT 06520, USA
11. Nanobiology Institute, Yale University, West Haven, CT 06477, USA
12. Department of Engineering Science, University of Oxford, Oxford, OX1 3PJ, UK
13. Centre for Neural Circuits and Behaviour, University of Oxford, OX1 3SR, UK

† These authors contributed equally to the work

*To whom correspondence should be addressed:
Joerg.Bewersdorf@yale.edu

Supplemental Experimental Procedures

1. Microscope Setup

A summary of the used components is provided at the end of this section. The microscope was built around a vertical bread board and a piezo/linear stage assembly (the white box in **Supplemental Figure S6A**) mounted on an air damped optical table (1200 by 1800 by 300 mm, 784 Performance Series, Technical Manufacturing Corporation). The piezo/linear stage assembly forms the first module of the interference cavity and holds the sample and both objective lenses and allows the sample position, interference cavity path length, and objective alignment to be adjusted remotely via computer control independent of the detection beam path (**Supplemental Figure S6**, **Supplemental Movie S7**). The vertical breadboard holds the second module of the interference cavity, deformable mirrors, and detection optics on its front surface and the excitation beam path on the back. Three excitation laser lines at wavelengths of 642 nm (MPB Communications, 2W), initially 561 nm (Coherent Genesis MX 56, 500 mW), later 560 nm (MPB communications, 2W) and 405 nm (Coherent OBIS 405 LX, 50 mW) reside on the optical table and are coupled into a polarization-maintaining single-mode fiber after passing through an acousto-optical tunable filter for wavelength selection and power modulation. The fiber delivers the excitation light to the back surface of the vertical breadboard where one of two optical paths may be selected via a pair of computer-controlled motorized mirrors (**Supplemental Figure S6C**). The first optical path illuminates an ~18 by 18 μm square area in the sample plane with nearly uniform illumination over the excitation field via an over-illuminated adjustable rectangular aperture. The fiber tip is conjugated to the objective back focal plane of the top objective lens and can be translated sideways for switching between epi-illumination and highly inclined and laminated optical sheet (HILO) imaging modalities. The second excitation path illuminates a ~100-μm diameter area for overview imaging and sample positioning.

Imaging is performed with two opposing high-NA oil immersion objective lenses (Olympus UPLSAPO 100XO PSF 1.4NA). The objective residing below the sample is mounted on a two-axis piezo stage (**Supplemental Figure S6B, blue stage**) (Physik Instrumente, P-612 2SL) which allows for lateral objective alignment with 5 nm resolution. The axial position of the upper objective is controlled with a nanopositioning stage (**Supplemental Figure S6B, green stage**) (Physik Instrumente, N-664.3A) with 0.5-nm resolution over a 16-mm travel range. Thus, the upper objective may be axially translated away from the lower objective for sample loading and then returned to the co-focal position with nanometer-accuracy. Additionally, a 940-nm diagnostic laser line is passed through the objective pair to facilitate real-time monitoring and correction of the axial and lateral objective alignment.

The axial position of the sample is controlled by a piezo stage (**Supplemental Figure S6B, magenta stage**) (Physik Instrumente, P-541.Z) with 0.5-nm resolution for precise and repeatable imaging of optical sections at various depths. This stage is, in turn, supported by a piezo-driven XY translation stage (**Supplemental Figure S6B, gray stage**) (Physik Instrumente, M-686.D64) with 100-nm resolution for coarse lateral sample positioning. The axial and XY sample stages are resting on a custom plate supported at three points of contact by three DC-Mike linear actuators (**Supplemental Figure S6B, gray columns**) (Physik Instrumente, N-227.10) with 100-nm repeatability for coarse axial positioning and tip/tilt sample adjustment. Two linear stages (**Supplemental Figure S6B, orange stages**) (ASI, LS-50) translate the entire objective-sample stage stack described above along the optical axis with a resolution of 10 nm allowing the optical path lengths of the two interference arms to be adjusted and maintained via computer control without affecting alignment in other areas of the system. **Supplemental Movie S7** shows an animation of the entire objective-sample stage stack assembly.

The back pupil planes of the upper and lower objectives are imaged onto two respective deformable mirrors (Boston Micromachines, Multi-5.5). 0.94x telescopes reduce the size of the objective pupils to match the active areas of the deformable mirrors. The deformable mirrors allow independent aberration compensation through both detection arms and facilitate aberration-free W-4PiSMSN imaging by correcting system and sample-induced aberrations.

A custom-made Babinet–Soleil compensator (UVisIR, custom BK7 and quartz blocks and wedges) allows system-level dispersion compensation and independent adjustment of the relative phase between the s- and p-polarizations in the two arms of the interference cavity (Aquino et al., 2011). The upper arm of the interference cavity includes a BK7 window bonded to a quartz wedge. A second quartz wedge is mounted adjacent to the first one on a motorized linear translation stage, allowing for relative phase adjustment between the two polarizations in the two cavity arms. The lower cavity arm includes a complementary quartz window bonded to a BK7 wedge. A matching BK7 wedge is also mounted on a motorized stage for dispersion compensation across the visible spectrum. Both cavity arms include electronically controlled shutters to allow recording PSFs through a single objective (either top or bottom) for independent aberration measurement and compensation. The final element in the interference cavity is a 50/50 beam splitter cube which is mounted on a motorized goniometer and rotation stage allowing the cube's reflective surface to be tipped and tilted without translation. This is critical for achieving uniform interference across the entire field of view and allows the beam splitter (BS) cube to be adjusted without perturbing alignment in other areas. In an arrangement similar to Aquino et al. (2011), relay optics direct the fluorescence exiting the 50/50 beam splitter cube along two separate paths to a single camera, in our case an sCMOS camera (Hamamatsu, ORCA-Flash 4.0v2) capable of recording 800 frames per second at 2048 x 256 pixels. This design allows us to project the four images (with ~$\pi/2$ phase delays between the images) along the center splitting line of the upper and lower sCMOS rolling readout regions and therefore allows uncompromised camera frame rates. A motorized filter wheel (84889, Edmund Optics) was mounted in front of the camera to allow sequential two-color imaging.

In addition, a motorized flip mirror and an overview camera (PCO, pco.pixelfly usb) provide ~100 µm diameter field of view overview images of the sample, which help in finding and positioning areas of interest in the sample. Table below lists vendors and part numbers of the major components.

Part	Vendor	Part number/names
561 nm Laser	MPB Communications	2RU-VFL-P-2000-560-B1R
560 nm Laser	Coherent	Genesis MX 56 500 mW
642 nm Laser	MPB Communications	2RU-VFL-P-2000-642-B1R
405 nm Laser	Coherent	OBIS 405nm LX 50mW Laser
Deformable Mirrors	Boston Micromachines	Multi-5.5
Quartz and BK7 window	UVISIR	Custom made (call #: W-4PiSMSN)
AOTF	AA OPTO-ELECTRONIC	AOTFnC-400.650-TN
Detection filters	Semrock	FF01-607/70-25
Detection filters	Chroma	ET700/75m
Piezo stages	PI	M-686.D64 XY Stage

Piezo stages	PI	P-541.ZCD Z Stage
Piezo stages	PI	P-612.2SL XY Stage
Piezo stages	PI	N-664.3A Linear Stage
Linear Actuator	PI	M-227.10
Vertical Translational Stage	ASI	LS-50 (FTP mode)
Camera	Hamamatsu	ORCA-Flash4.0 V2 Digital CMOS Camera 22CU
Quad bandpass filter	Semrock	FF01-446/523/600/677-25
Dichroic mirrors	Semrock	405/488/561/635 BrightLine Laser Dichroic
Objectives	Olympus	UPLSAPO 100XO
Analysis software	MathWorks	MATLAB
GPU	Nvidia	GeForce GTX 580
Visualization software	Multiple	Vutara (Bruker), Avizo (FEI Software), Maya 2015 (Autodesk)

2. Characterization of Deformable Mirrors

Deformation of the reflective membrane on the BMC Multi-5.5 deformable mirror (DM) is induced by a set of 140 actuators positioned in a grid-patterned array beneath the membrane. Each actuator adds a degree of freedom to the shape of the deformable mirror. As previously shown (Wang and Booth, 2009), the possible mirror shapes can be decomposed into an orthogonal set of so-called mirror deformation eigenmodes (in short, **mirror modes**), resembling Zernike modes while accounting for the stiffness of the membrane and the spatial distribution of the actuators. One mirror mode is essentially a set of displacements applied to the actuator array. To establish the actual shape of the mirror when applying a given mirror mode, a DM characterization process was followed as detailed previously (Burke et al., 2015). This process relies on a phase-retrieval scheme (Hanser et al., 2004) (using the Gerchberg-Saxton algorithm) and takes as input a set of images of a point emitter at known axial positions near the objective focal plane. We used a sub-diffraction sized fluorescent bead (100 nm crimson, Life Technologies) and imaged it at five known axial positions: z = -1 µm, -0.5 µm, 0 µm, 0.5 µm and 1 µm, where z = 0 µm corresponds to the objective focal plane. Given one such three-dimensional image set, the phase retrieval scheme (Hanser et al., 2004) provides an estimate of the pupil function, i.e. the wave-front shape at the objective pupil plane.

For each mirror mode, we retrieved pupil functions for five different mirror mode amplitudes. Subsequently, the pupil functions were each decomposed into a set of Zernike modes. By fitting a first-order polynomial through the Zernike mode coefficients as a function of mirror mode amplitudes, we determined the first 55 Zernike mode coefficients for each of the applied 28 mirror modes. The resulting coefficients were used to form an underdetermined system of 28 linear equations each describing the Zernike mode constituents of a single mirror mode. By solving this system of linear equations in the least square sense, each Zernike mode can be expressed as a linear combination (weighted sum) of mirror modes. This calibration process was carried out separately for the top and bottom interference arms.

3. System Aberration Correction

System aberrations for the upper and lower beam paths were corrected separately. The corresponding deformable mirrors were independently adjusted as follows. For each interference arm, starting from the flat voltage map (provided by the manufacturer) of the deformable mirror, 28 mirror modes (Wang and Booth, 2009) were applied sequentially. For each mirror mode, 10 different amplitudes were applied while recording the corresponding fluorescence signal from a 100-nm crimson bead sample. To extract the fluorescence signal from individual beads, the symmetry center of each imaged bead was obtained using the radial symmetry method (Parthasarathy, 2012). Subsequently, a symmetric 2D Gaussian was generated at the symmetry center and was multiplied by the isolated emission pattern from the fluorescent bead, generating a Gaussian-masked image, and then the total intensity of the masked image was calculated to extract the center peak signal of the beads in focus. For each mirror mode, images of the bead were acquired at ten different mirror mode amplitudes and the corresponding center peak signals of the bead were extracted as described above. The optimal amplitude (i.e. the amplitude providing the highest center peak signal from the beads) was determined from a quadratic fit of these ten signal measurements vs. mirror mode amplitudes. After identifying optimal amplitudes for each of the 28 modes, these amplitudes were added to the flat voltage map (provided by the manufacturer), serving as a starting point for another iteration. This iterative process was repeated five times to achieve optimal system aberration correction.

4. Channel Registration

Each sCMOS camera frame contains four images (arranged next to each other) that represent the same field of view of the sample at different interference phase delays (Aquino et al., 2011). In the following, we refer to these images as ***phase images***. One of the phase images was taken as the reference, and all the other three phase images were merged (added) into the reference image using three affine transformations (one for each phase image) where the transformations were obtained from a calibration bead data set taken prior or after the imaging session (200 frames of approximately 10-15 beads in focus imaged with a single objective). To ensure accurate merging of the four phase images, our estimation of each transformation matrix (including magnification, translation and rotation) followed the following steps: first, a Fourier-Mellin transform (implemented as “fmmatch” using the MATLAB dipimage toolbox, www.diplib.org) was used to obtain an initial estimation of the transform. Second, the affine transform was then obtained using the previous result from Fourier-Mellin transform as an initial guess (implemented as “find_affine_trans” using the dipimage toolbox). These two sequential steps took advantage of the fact that the log-polar transformation is invariant with translation in the image and the least-square approach to find affine transformations works well when the relative shifts between two images are small.

5. Lateral Position Localization

To estimate the sCMOS camera characteristics (including readout noise, offset and gain for each pixel) in the combined frame described in the section above, noise statistics maps from all four phase images were merged in the same way as the phase images and subsequently used as the noise map in the subsequent fitting process (Huang et al., 2013).

As described previously (Huang et al., 2013), a series of uniform and maximum filters were used to find isolated single molecules and sub-regions were cropped around these fitting candidates. The isolated sub-regions were then fit using the Maximum Likelihood Estimator (MLE) with the sCMOS noise model (Huang et al., 2013) to an elliptical 2D Gaussian (Huang et al., 2008) and estimates of single-molecule positions (x, y), standard deviations (σ_x, σ_y), total number of detected photons, background photon counts and log-likelihood ratio values were obtained. The threshold of the log-likelihood ratio metric (Huang et

al., 2011) (used as the goodness of fit test) was set to a relatively large value with the intention to filter out sub-regions containing multiple emitters.

6. Extraction of Single-molecule Phase

To estimate the phase of the single-molecule interference (a prerequisite to determine the z-position of the molecule), the estimated lateral single-molecule positions (x, y) were inverse-transformed (affine) back to the four separate phase images. Each of these positions pinpoints the center location of the single molecule in the four phase images in W-4PiSMSN. Using these center locations of single molecules in the four phase images, the 0th moment intensities (Aquino et al., 2011) were calculated by a weighted least-square fit of a Gaussian. As the center location of the molecule is already known, the weighted least-square fit was used to estimate the amplitude. Due to the pixel-dependent readout noise of sCMOS cameras, the weight for each pixel i is assigned as $D_i + \frac{\sigma_i^2}{g_i^2}$, where D_i is the pixel count, and σ_i^2 and g_i are the pixel-dependent readout noise variance and gain of the sCMOS sensor, respectively. In this way, we take the sCMOS-specific pixel-dependent noise into account (Huang et al., 2013).

The phase shift between s and p-polarization (Aquino et al., 2011) was adjusted such that it is close to π/2 for both detection channels. However, due to dispersion, this phase shift differed by ~0.3 radians in our system for our two imaging channels (607 nm and 700 nm center wavelengths). We calibrated the phase shifts for the two color channels independently using a bead sample which could be observed in both color channels (100 nm crimson beads).

To extract the interference phase of each single molecule, similar to the method previously described by Aquino et al., 2011, we extracted the 0th moment amplitudes of each single molecule from the four phase images and subsequently the reduced moments (Aquino et al., 2011), RM_s and RM_p, were calculated. Using the previously calibrated phase shifts, we obtained the phase of the single-molecule interference PSF by solving the set of equations (1).

$$\begin{cases} A_0 \cos(\varphi_0) - RM_s = 0 \\ A_0 \cos(\varphi_0 + \varphi_{shift\_ch}) - RM_p = 0 \end{cases} \qquad \text{Eq. 1}$$

A_0 and φ_0 are the unknowns representing the amplitude and single molecule phase, respectively. φ_{shift_ch} was previously obtained through the calibration using the bead sample for a specific channel and RM_s and RM_p are the reduced moments of 0th order (Aquino et al., 2011). For two-color imaging, the values of φ_{shift_ch} were different for the two different wavelength channels and were obtained for each color channel through the calibration.

7. Axial Localization Based on Monotonic Metric and Local Ridge Detection

Accurate and precise axial position estimation can be challenging when based only on astigmatism because even a small amount of sample-induced aberrations creates image distortions and artifacts which cause the PSF to deviate from the calibration curve (Liu et al., 2013; McGorty et al., 2014). This effect is increasingly problematic when imaging deep into a sample. Single-molecule interference, however, only relies on the path-length differences between the two interference arms and thus provides the opportunity for accurate large volume super-resolution imaging. Due to these concerns, we did not use the astigmatic shape information for our axial localization, but solely to unwrap the single-molecule phase and thus avoid distortions and artifacts as described below.

We started with a segment of W-4PiSMSN data (usually 3,000-5,000 frames, 10-200 segments per dataset) containing single-molecule emissions events. For each single-molecule emission, the localization methods described above allowed us to extract the interference phase φ_0 and standard deviations of the

2D Gaussian σ_x and σ_y. Thus, we obtained a list of these values for all single-molecule detection events in the data segment. Next, we introduced a metric, $m = \frac{{\sigma_x}^3}{\sigma_y} - \frac{{\sigma_y}^3}{\sigma_x}$, which describes the overall shape of the emission events and preserves its monotonicity in the presence of a small amount of aberrations. For numerical convenience, we normalized $\boldsymbol{m}$ by 2π/40. From this list of $\{m\}$ and $\{\varphi_0\}$ values for all single-molecule detection events in the data segment, we generated a 2D histogram image. **Supplemental Figure S3 A** shows a contour plot of such a histogram. As m is monotonic against the axial position and φ_0 is periodic with a period of 2π, the resulting 2D histogram resembles tilting stripes which are repeated over the range of the (normalized) metric m (**Supplemental Figure S3 A**).

Using the histogram, the problem is now reduced to a phase unwrapping problem. We seek to find a continuous phase variation, φ_0, with respect to m in an ensemble collection of single-molecule detection events**.** To do this, we developed a ridge finding algorithm that incrementally follows the peak of these stripes with the following three properties: first, it self-adapts to the shape and curvature of the stripes (**Supplemental Figure S3 B**). These shapes and curvatures are sample and depth-dependent and also vary between data segments within a single dataset. Second, the algorithm finds a continuous ridge through the histogram without jumping to the adjacent ridge (**Supplemental Figure S3 B**). This is important because errors in this aspect cause errors in the unwrapping step that lead to localization artifacts and ghost images in the final image (**see paragraph below**). Third, the algorithm is monotonic in the sense that no two points on the growing path share the same value of m to ensure unambiguous position assignment of single molecules.

To find the ridge along the wrapping histogram stripes, we started with an initial peak-finding process: the maximum peak spot was first identified (P_0) and the second peak (P_1) was then identified by searching within a radius range defined by r_{min} and r_{max} (**Supplemental Figure S3A**) from P_0. The search range restriction was used to allow the generation of an initial vector ($\overrightarrow{P_0P_1}$) that points along the direction of the running ridge (**Supplemental Figure S3A**). Next, we searched for the next peak starting from P_1. We assigned the search radius range as r_{min} and r_{max} centering around P_1 and also assigned the direction (or vision) of the search (**Supplemental Figure S3A**) in the direction of $\overrightarrow{P_0P_1}$ with ~0.2 rad angle width (adjustable parameter) forming a cone shaped "search vision". The next peak, P_2, was identified by finding the pixel with maximum value on the 2D histogram within the search range and search vision centered around P_1. Subsequently, P_2 served as the starting point for the next search, and so on. In this way, the path grew up to P_n where the value of the 2D histogram at the next peak P_{n+1} was less than a threshold. Therefore, the path $P_0P_1 \dots P_n$ identified half of the ridge of the histogram. Next, we used the same approach to find the path in the opposite direction (starting with P_0 with a initial direction of ($\overrightarrow{P_1P_0}$)) and generated the other half of the path $P_1P_0 \dots P_{-k}$. The two paths (forward and backward) were subsequently combined ($P_{-k} \dots P_0P_1 \dots P_n$) providing a piece-wise monotonic, self-adapting and unambiguous curve through the phase-metric plot.

With the piece-wise monotonic path, we can now un-wrap it (Judge and Bryanston-Cross, 1994) into a monotonic path (implemented using "unwrap" in MATLAB). For each single-molecule detection event, with a corresponding m and φ_0 pair, we determined the point P_{path} on the piece-wise monotonic path that gives the minimum distance to point (m, φ_0). Subsequently, we unwrapped the (m, φ_0) together with P_{path} by adding the same integer multiplication of 2π to (m, φ_0) as it was added during the unwrapping process for P_{path}. Therefore, with the help of our shape metric m, this process unwrapped $\{\varphi_0\}$ and resolved the ambiguity due to its periodicity. To allow accurate translation of phase-unwrapped values $\{\varphi_0\}$ to axial position estimates $\{z_{est}\}$, we simulated W-4PiSMSN PSFs using a pupil function-based approach (Hanser et al., 2004). W-4PiSMSN PSFs were simulated for both detection channels (607 nm and 700 nm). Modulation frequencies (unit: radians (φ_0, phase) per nm (z_{est}, axial position)) were

identified in these PSFs and were used to translate the phase into axial positions in both single-color and two-color measurements.

8. 3D Drift Correction with Redundancy

Based on the idea of the previously published redundancy-based drift correction method in 2D (Li et al., 2013; Wang et al., 2014), we developed a 3D drift correction. First, similar to other drift correction algorithms (Mlodzianoski et al., 2011), the entire dataset was split into n segments (usually 3,000-5,000 frames each, resulting in n = 10 to 200 segments per dataset). For each data segment, a volume image with (25 nm)^3 voxel size was reconstructed as a 3D histogram where the count for each pixel in the histogram equals the number of localization estimates falling into the voxel. 3D cross-correlation was then used to calculate the shift distance between each pair of data segment volumes. To find the correlation peak from the 3D cross-correlation image, Fourier interpolation was used to identify the peak with an effective pixel size of 1.2 nm in the final interpolated image. This process pinpoints the shift distance $(x_{shift}^{i\to j}, y_{shift}^{i\to j}, z_{shift}^{i\to j})$ between two data segment volumes (i and j). For a total of n data segments, there are $\frac{1}{2}n(n+1)$ of such shift measurements (which are not independent) forming an overdetermined system to determine independent shifts between adjacent data segments, $\{(x_{shift}^{0\to 1}, y_{shift}^{0\to 1}, z_{shift}^{0\to 1}), \ldots, (x_{shift}^{i\to i+1}, y_{shift}^{i\to i+1}, z_{shift}^{i\to i+1}), \ldots, (x_{shift}^{n-1\to n}, y_{shift}^{n-1\to n}, z_{shift}^{n-1\to n})\}$, including a total of $n-1$ unknown independent shifts. For each coordinate (x, y and z), a system of linear equations was established as described in Li et al., 2013. Least-square solutions that minimize the overall error of the equation stacks were calculated and substituted back into all equations. Residual errors can be calculated for each of these equations and an equation is removed from the system of linear equations if its error is larger than 7 nm. This process was repeated until no single equation gave an error larger than 7 nm or the system of linear equations was no-longer at its full rank.

9. Data Analysis for Multi-optical Section Data

To image thick samples, optical sections were recorded at different axial positions of the sample by axially translating the z-piezo holding the sample stage. The localization data contains x, y, and z position estimates of different optical sections and must be aligned/stitched seamlessly to support the high precision obtained in W-4PiSMSN. Previous methods (Huang et al., 2008) that shift each optical section by a constant in the axial direction have been prone to introduce misalignment of the optical sections and subsequently deteriorate the resolution achievable in thick samples. Here, we developed an optical alignment method based on 3D cross-correlation. In the W-4PiSMSN system, optical sections are ~1.2 µm thick. Whole-cell samples were scanned in the axial direction with 500-nm step sizes which allowed for abundant overlapping regions between adjacent optical sections. This overlapping information between optical sections is critical for precise optical section alignment using the cross-correlation methods described below.

Similar to **Supplemental Information 8**, for each data segment (an optical section in this case), a volume image with (25 nm)^3 voxel size was reconstructed as a 3D histogram where the count for each pixel in the histogram equals the number of localization estimates within the voxel. Given a 500-nm axial step size of the sample stage, we observed an effective shift of only ~400 nm between adjacent reconstructed optical sections. This inconsistency is explained by the index of refraction mismatch between the sample (~1.33-1.37) and the immersion media (~1.51). Therefore, expecting a peak around 400 nm in our correlation volume (generated as described in **Supplemental Information 8**), we isolated a small 3D sub-volume around this expected center and determined the local peak within this sub-volume. In this way, we isolated the desired peak from the noise-induced peaks that are usually located in the center of the correlation volume. The noise induced center peak is especially strong and thus problematic when two images (in our case volumes) only partially overlap, which is here the case. As described above, to find

the local correlation peak from the 3D cross-correlation sub volume, Fourier interpolation was used to identify the peak with an effective pixel size of 1.2 nm in the final interpolated image (Li et al., 2013). The resulting peak identifies shifts between the two adjacent optical sections.

10. Multi-color Data Alignment

Two-color imaging was performed sequentially, first in the Alexa Fluor 647 (AL647) channel and then the Cy3B channel. Specifically for two-color imaging, we shifted all the AL647 data segment volumes in the drift correction step (**Supplemental Information 8**) to align with the *last* data volume at the end of the AL647 imaging session. For the Cy3B channel, we aligned all Cy3B data segment volumes with the *first* data segment volume in the beginning of the Cy3B imaging session. There was a 2-4 s pause between the recording of the two color channels and we assumed system drift and sample drift during this interval to be negligible.

To align 3D volumes after drift correction from one color channel to another, we obtained a 3D affine transformation from a training dataset using double-stained (AL647 and Cy3B) mitochondria (implemented as “imregtform” in MATLAB) from 3D histogram image as described in **Supplemental Information 8** and **9** with a voxel size of $(25\ \text{nm})^3$. The obtained registration matrix (affine) was then applied to all single-molecule position estimates from one channel to allow 3D color registration of two color volumes.

11. Cilia Membrane Flattening

First, a manually selected region of interest (ROI) is fit (least square) with a cylinder model. Then the region was aligned such that the cylinder axis became the z-axis and the ROI was centered at the origin of the coordinate system. Based on the new coordinate system, each single-molecule localization was transformed into a modified version of cylindrical coordinates (ρ, φ, z). Surface plots were obtained by generating a 2D histogram of the cylindrical coordinates of single molecules as ρ - r and φ, where r is the radius of the fitted cylinder model.

12. Template-Free Point Cloud Registration for T7 Phage Data

A total of 115 T7 phages, each represented by a point cloud, were automatically identified from 14 W-4PiSMSN datasets. The orientation and the structural center were obtained by fitting the point cloud to a straight line and sphere, respectively. All phages were then centered at the origin of a Cartesian coordinate system and rotated such that their orientation (icosahedron capsid) aligned with the z-axis. These pre-aligned phages were then aligned to each other using Gaussian kernel correlation registration (Tsin and Kanade, 2004), with a simplified version of the pyramid scheme described previously (Broeken et al., 2015), by allowing it to rotate around the z-axis. This second alignment step created an initial guess for the final phage averaged structure. The final averaged structure was obtained by aligning all original phage results to the initial guess. To improve performance of the registration algorithm, a fast Gauss transform (Jian and Vemuri, 2011) was implemented.

13. Point Cloud Clustering Algorithm to Isolate Synaptonemal Complexes

Paired strands of synaptonemal complexes (**Figure 7**) were isolated using a clustering algorithm (Klasing et al., 2008) where single-molecule localization estimates located within a 500-nm distance from each other were assigned to the same cluster. The algorithm terminated when all points were processed and assigned to clusters. To avoid over-counting caused by unspecific labeling, final clusters with fewer than 750 points were discarded.

14. Noise Reduction

To allow robust and precise feature detection and alignment in point cloud data, the point clouds were first processed through a noise filter where all points whose number of neighbor points within a given distance was below a user-defined threshold were discarded. While this noise filter was only used to improve feature detection and model construction, the raw single-molecule localization results (without de-noising) were used after the alignment process to provide quantifiable final results. This method was applied to help feature extraction in phage, cilia and synaptonemal complex datasets and to generate the shown phage averaged reconstruction (**Figure 1I-L**) and images of isolated strands of synaptonemal complex (**Figure 7 E and F**).

15. Coverslip Preparation and Cell Culture

25 mm diameter round precision glass cover slips (Bioscience Tools, San Diego, CA) were immersed in 1M KOH and sonicated for 15 min in an ultrasonic cleaner (2510 Branson, Richmond, VA). The glass was then generously rinsed with Milli-Q water (EMD Millipore, Billerica, MA) and sterilized with 70% ethanol. The glass was dried and poly-L-lysine coated before 100-nm Crimson beads (Life Technologies, Grand Island, NY) were attached to the top surface. Before cells were plated on the beads, the surface was rinsed three times with Phosphate-buffered saline (**PBS**). Cells were grown on coverslips for 2-24 hours before fixation.

COS-7 cells (ATCC, CRL-1651) were grown in DMEM (Gibco, 21063-045) with 10% Fetal Bovine Serum (FBS) and 1% Penicillin-Streptomycin (Gibco, 15140-122) at 37 °C with 5% CO_2. BSC1 cells (ATCC, CCL-26) were grown in DMEM (Gibco, 21063-045) with 10% FBS at 37 °C and 5% CO_2. RPE-hTERT cells were grown in DMEM/F12 (Gibco 11330-032) with 10% FBS and 1% Antibiotic-Antimycotic (Gibco, 15240-062) at 37 °C and 5% CO_2.

16. Secondary Antibody Labeling

Except where noted otherwise, primary antibodies were labeled with Alexa Fluor 647-conjugated goat anti-mouse or goat anti-rabbit secondary antibodies (Thermo Fisher Scientific, A21236, A21245, Waltham, MA). Secondary antibodies labeled with Cy3B were made by reacting Cy3B NHS Esters (GE Healthcare, Malborough, MA) with unlabeled secondary antibodies (Jackson ImmunoResearch Laboratories, Inc., West Grove, PA) according to the manufacturer's protocol. Free dye was separated from labeled antibody by gel filtration using an illustra NAP-5 column (GE Healthcare). Samples were labeled with secondary antibodies at a dilution between 1:1000 and 1:200 for 30 to 60 min at room temperature. Where noted, a post-fixation step of 3% paraformaldehyde (PFA, Electron Microscopy Sciences, 15710, Hatfield, PA) + 0.1% glutaraldehyde (GA, Electron Microscopy Sciences, 16019, Hatfield, PA) was performed after secondary antibody labeling. Samples were rinsed three times with PBS and stored in PBS until they were imaged.

17. Endoplasmic Reticulum Samples

COS-7 cells were grown on prepared coverslips and then transfected with *mEmerald-Sec61-C-18*, a gift from Michael Davidson (Addgene plasmid # 54249), using Lipofectamine2000 (Thermo Fisher Scientific). 12-24 h later, cells were fixed using 3% PFA + 0.1% GA in PBS for 15 min. Cells were permeabilized for 3 min at room temperature with 0.3% IGEPAL-630 (Sigma-Aldrich) + 0.05% Triton X-100 (Sigma-Aldrich) + 0.1% BSA in PBS. Samples were blocked with blocking buffer (5% normal Goat serum, 0.05% IGEPAL-630, 0.05% Triton X-100 in PBS). Rabbit anti-GFP (Thermo Fisher Scientific, A-11122) was used at 1:500 to label mEmerald-Sec61β overnight at 4 °C. Antibodies were diluted in blocking buffer. Samples were washed in wash buffer (WB, 0.2% BSA, 0.05% IGEPAL-630, 0.05% Triton X-100 in PBS) for 5 min three times before labeling with secondary antibody for 1 h at

room temperature. Samples were then washed again in WB for 5-min incubations three times before they were post-fixed with 3%PFA+0.1%GA.

18. Microtubule Samples

Microtubule samples were prepared similar to our previous report (Huang et al. 2013). COS-7 cells were grown on prepared coverslips. Cells were rinsed three times with 37 °C PBS before a 1-min pre-extraction incubation in pre-warmed 0.2% saponin in cytoskeleton buffer (CBS, 10 mM MES pH 6.1, 138 mM NaCl, 3 mM $MgCl_2$, 2 mM EGTA, 320 mM sucrose) to remove tubulin monomers from the cell cytoplasm. Immediately following the pre-extraction step, the cells were fixed for 15 min at room temperature in 3% PFA and 0.1% GA diluted in CBS. After fixation, the cells were rinsed three times in PBS before being permeabilized and blocked in blocking buffer (3% bovine serum albumin (BSA, Jackson ImmunoResearch) and 0.2% Triton X-100 in PBS) for 30 min at room temperature. Mouse anti-α-tubulin antibody (Sigma-Aldrich, T5168, St. Louis, MO) was used at 1:1000 dilution for a 4 °C overnight incubation. Antibodies were diluted in 1% BSA and 0.2% Triton X-100 in PBS. Cells were washed three times for 5 min each in wash buffer (WB, 0.05% Triton X-100 in PBS). Secondary antibodies were used to label cells for 1 h at room temperature. Cells were washed again in WB for 5-min incubations three times and then post-fixed with 3% PFA + 0.1% GA for 10 min. Samples were rinsed three times with PBS.

19. T7 Bacteriophage Samples

T7 bacteriophage lysate was prepared from 100 ml of *E. coli* MG1655 cultures grown in liquid broth (LB) at 30 °C. The phage lysate was subjected to PEG precipitation and cesium chloride-gradient centrifugation as described in Chan et al., 2005 with some modifications. Following the PEG precipitation step, ~300 µl of phage in borate buffer (50 mM borate, pH 8.5) was incubated with 100 µg of Alexa Fluor 647 NHS Ester (4 µg/µl in DMSO; Thermo Fisher Scientific) for 30 min at room temperature. The labeled phage particles were first purified using a Bio-Spin P30 column (Bio-Rad Laboratories, Hercules, CA) to remove most of the free dye and then subjected to cesium chloride gradient centrifugation. Cesium chloride was removed using a Bio-Spin P30 column and phage particles were eluted in T7 storage buffer (10 mM Tris pH 7.5, 10 mM $MgCl_2$).

Coverslips were cleaned as described in (Lim et al., 2014). Briefly, the coverslips were cleaned by sonication in 1M KOH, double-distilled H_2O, and 70% ethanol for 15 min each at room temperature. 0.1% poly-L-lysine was added to the cleaned coverslip, incubated for 30 min at room temperature, washed with T7 storage buffer, and then dried with pressured air. For imaging, 20 µl of the phage lysate was spotted onto cleaned coverslip, incubated for 1 min at room temperature, and then washed extensively with T7 storage buffer. The coverslip was air-dried before imaging.

20. Mitochondria Samples and Two-color Mitochondria & Microtubule Samples

COS-7 cells were grown on prepared coverslips and fixed using 3% PFA + 0.1% GA in PBS for 15 min. Cells were permeabilized for 3 min at room temperature with 0.3% IGEPAL-630 + 0.05% Triton X-100 + 0.1% BSA in PBS. Samples were blocked with blocking buffer (5% normal Goat serum, 0.05% IGEPAL-630, 0.05% Triton X-100 in PBS). Rabbit anti-TOM20 (Santa Cruz Biotechnology sc-11415, Dallas, TX) was used at 1:500 and mouse anti-α-tubulin (Sigma-Aldrich, T5168) was used at 1:1000 and they were incubated with samples overnight at 4 °C. Antibodies were diluted in blocking buffer. Cells were washed in wash buffer (WB, 0.2% BSA, 0.05% IGEPAL-630, 0.05% Triton X-100 in PBS) for 5-min incubations three times. Cells were labeled with secondary antibodies for 1 h at room temperature. Then samples were washed again with WB for 5 min three times before they were post-fixed with 3% PFA + 0.1% GA.

21. Nuclear Pore Complexes Samples

hTERT-RPE1 cells were grown to 100 percent confluence on prepared coverslips. Cells were pre-permeabilized with pre-warmed 0.1% Saponin in PBS for 1 min. Cells were then rinsed with pre-warmed PBS and fixed with methanol at -20 °C for 5 min. Samples were blocked with 5% BSA + 0.1% Triton X-100 in PBS. After blocking, cells were labeled with goat anti-rabbit Nup358 for 1 h at room temperature. Antibodies were diluted in 1% BSA + 0.1% Triton X-100 in PBS. Cells were washed three times with PBS for 10 min each. Primary antibodies were labeled with secondary antibodies for 1 h at room temperature. Cells were washed three times in PBS for 10 min each. After washing, cells were post-fixed with 2% PFA in PBS for 2 minutes.

22. COPI Samples

BSC1 cells were grown on prepared coverslips and fixed with 4% paraformaldehyde in PBS for 15 min. Cells were permeabilized for 3 min at room temperature with 0.3% IGEPAL-630 + 0.05% Triton X-100 + 0.1% BSA in PBS. Samples were blocked in blocking buffer (5% normal Goat serum, 0.05% IGEPAL-630, 0.05% Triton X-100 in PBS). Mouse anti- ’ COP (Palmer et al., 1993) was used to label COPI at 1:2000 dilution in blocking buffer overnight at 4 °C. Cells were washed in wash buffer (WB, 0.2% BSA, 0.05% IGEPAL-630, 0.05% Triton X-100 in PBS) three times for 5 min each before labeling with secondary antibody for 1 h at room temperature. Samples were then washed with WB for 5 min three times before being post-fixed with 3% PFA + 0.1% GA.

23. Cilia Samples

hTERT-RPE1 cells that stably express pHlourin-Smoothened (pH-SMO) were grown on prepared coverslips. To induce ciliogenesis, the cells were incubated in DMEM/F12 media with 0.5% FBS and 100 nm Cytochalasin D for 48 h.

After ciliogenesis induction, cells were washed twice in PBS and fixed for 10 min with 4% PFA + 0.2% GA + 0.1% Triton X-100 in PBS. Fixed cells were then washed twice with PBS + 0.05% Tween20 followed by a 30 min incubation in blocking buffer (5% BSA + 0.05% Tween20 in PBS). Cells were incubated with the primary rabbit anti-GFP antibody (Thermo Fisher Scientific, A11122) at 1:500 dilution in blocking buffer for 1 h at room temperature. Then cells were washed with three 5 min incubations in wash buffer (0.05% Tween20 in PBS). Cells were labeled with secondary antibody diluted in blocking buffer for 30 min at room temperature. Samples were washed three times for 5 min each in wash buffer, and followed by two rinses in PBS.

24. Synaptonemal Complex Samples

Testes were removed from 17-18 day old euthanized mice. The protocols for the care and use of mice at suitable ages were approved by the Institutional Animal Care and Use Committee (IACUC) of The Jackson Laboratory. Each testis was disrupted in PBS supplemented with protease inhibitors using a razor blade. The cell pellet was collected after centrifugation at 9,000 rpm for 10 min. The cells were resuspended and allowed to settle on prepared coverslips. They were then fixed with 4% PFA for 15 min, rinsed with PBS three times, and permeabilized with 0.5% Triton X-100 in PBS for 10 min. Before incubating with primary antibody, spermatocytes were treated with Image-iT signal Enhancer (Thermo Fisher Scientific) and blocked with MAXblock (Active Motif). Cells were stained with anti-SYCP3 (Abcam, ab15093, Cambridge, MA) overnight at 4 °C. Cells were then washed three times for 5-min incubations in wash buffer (WB, 0.1% Triton X-100 in PBS) before labeling with secondary antibodies for 3 h at 37 °C. Cells were washed again three times for 5 min each in WB.

25. Imaging Buffers

Two different imaging buffers were used.

The conventional β-mercaptoethanol imaging buffer was prepared as previously reported (Huang et al 2013). The imaging buffer was made immediately before use where catalase and glucose oxidase were diluted in base buffer (50 mM Tris pH 8.0, 50 mM NaCl, 10% glucose).

The imaging buffer containing cyclooctatetraene (COT) was prepared according to a previously published report (Olivier et al., 2013). Mercaptoethylamine (MEA, Sigma-Aldrich, 30070) was dissolved in deionized water as 1M stock solution, and then adjusted to pH 8 by glacial acetic acid (Avantor Performance Materials). The stock solution was stored at 4 °C and used within a week. β-mercaptoethanol (BME, Sigma-Aldrich, 63689) was used without dilution as 14.3 M solution. Cyclooctatetraene (COT, Sigma-Aldrich, 138924) was diluted in DMSO as 200 mM stock solution and stored at 4 °C. Protocatechuic acid (PCA, Sigma-Aldrich, 37580) was dissolved in deionized water as 100 mM stock solution, then adjusted to pH 9 by KOH aq. The stock solution was stored at 4 °C and used within a month. Protocatechuate 3,4-dioxygenase from *Pseudomonas* sp. (PCD, Sigma-Aldrich, P8279) was dissolved in 100 mM Tris-HCl (pH 8) containing 50 mM KCl, 1 mM EDTA and 50% glycerol as 5 μM stock solution, and stored at −20 °C. The imaging buffer consists of base buffer (50 mM Tris pH 8.0, 50 mM NaCl, 10% glucose) with the addition of 10 mM MEA, 50 mM BME, 2 mM COT, 2.5 mM PCA and 50 nM PCD. The buffer was prepared immediately before use.

26. Sample-mounting in W-4PiSMSN

Prepared sample coverslips were drained and subsequently mounted on a custom-designed sample holder. A custom-made spacer ring (9513K111, McMaster-Carr, Princeton, NJ) was put on top of the sample coverslip and then 50 μL imaging buffer as described above, was added to the center of the coverslip. Another coverslip was put on top and excess imaging buffer was drained. The samples were then sealed with two-component silicone putty (Picodent Twinsil, Picodent, Wipperfürth, Germany). After solidification of the silicone, the samples were transferred to the W-4PiSMSN microscope for imaging.

Supplemental References

Aquino, D., Schönle, A., Geisler, C., Middendorff, C. V, Wurm, C.A., Okamura, Y., Lang, T., Hell, S.W., and Egner, A. (2011). Two-color nanoscopy of three-dimensional volumes by 4Pi detection of stochastically switched fluorophores. Nat. Methods *8*, 353–359.

Broeken, J., Johnson, H., Lidke, D.S., Liu, S., Nieuwenhuizen, R.P.J., Stallinga, S., Lidke, K.A., and Rieger, B. (2015). Resolution improvement by 3D particle averaging in localization microscopy. Methods Appl. Fluoresc. *3*, 014003.

Burke, D., Patton, B., Huang, F., Bewersdorf, J., and Booth, M.J. (2015). Adaptive optics correction of specimen-induced aberrations in single-molecule switching microscopy. Optica *2*, 177.

Chan, L.Y., Kosuri, S., and Endy, D. (2005). Refactoring bacteriophage T7. Mol. Syst. Biol. *1*, 2005.0018.

Hanser, B.M., Gustafsson, M.G.L., Agard, D.A., and Sedat, J.W. (2004). Phase-retrieved pupil functions in wide-field fluorescence microscopy. J. Microsc. *216*, 32–48.

Huang, B., Jones, S.A., Brandenburg, B., and Zhuang, X. (2008). Whole-cell 3D STORM reveals interactions between cellular structures with nanometer-scale resolution. Nat. Methods *5*, 1047–1052.

Huang, F., Schwartz, S.L., Byars, J.M., and Lidke, K.A. (2011). Simultaneous multiple-emitter fitting for single molecule super-resolution imaging. Biomed. Opt. Express *2*, 1377–1393.

Huang, F., Hartwich, T.M.P., Rivera-Molina, F.E., Lin, Y., Duim, W.C., Long, J.J., Uchil, P.D., Myers, J.R., Baird, M.A., Mothes, W., et al. (2013). Video-rate nanoscopy using sCMOS camera-specific single-molecule localization algorithms. Nat. Methods *10*, 653–658.

Jian, B., and Vemuri, B.C. (2011). Robust point set registration using Gaussian mixture models. IEEE Trans. Pattern Anal. Mach. Intell. *33*, 1633–1645.

Judge, T.R., and Bryanston-Cross, P.J. (1994). A review of phase unwrapping techniques in fringe analysis. Opt. Lasers Eng. *21*, 199–239.

Klasing, K., Wollherr, D., and Buss, M. (2008). A clustering method for efficient segmentation of 3D laser data. In 2008 IEEE International Conference on Robotics and Automation, (IEEE), pp. 4043–4048.

Li, X., Mooney, P., Zheng, S., Booth, C.R., Braunfeld, M.B., Gubbens, S., Agard, D.A., and Cheng, Y. (2013). Electron counting and beam-induced motion correction enable near-atomic-resolution single-particle cryo-EM. Nat. Methods *10*, 584–590.

Lim, H.C., Surovtsev, I. V., Beltran, B.G., Huang, F., Bewersdorf, J., and Jacobs-Wagner, C. (2014). Evidence for a DNA-relay mechanism in ParABS-mediated chromosome segregation. Elife *2014*.

Liu, S., Kromann, E.B., Krueger, W.D., Bewersdorf, J., and Lidke, K.A. (2013). Three dimensional single molecule localization using a phase retrieved pupil function. Opt Express *21*, 29462–29487.

McGorty, R., Schnitzbauer, J., Zhang, W., and Huang, B. (2014). Correction of depth-dependent aberrations in 3D single-molecule localization and super-resolution microscopy. Opt. Lett. *39*, 275–278.

Mlodzianoski, M.J., Schreiner, J.M., Callahan, S.P., Smolková, K., Dlasková, A., Šantorová, J., Ježek, P., and Bewersdorf, J. (2011). Sample drift correction in 3D fluorescence photoactivation localization microscopy. Opt. Express *19*, 15009.

Nieuwenhuizen, R.P.J., Lidke, K.A., Bates, M., Puig, D.L., Grünwald, D., Stallinga, S., and Rieger, B. (2013). Measuring image resolution in optical nanoscopy. Nat. Methods *10*, 557–562.

Olivier, N., Keller, D., Gönczy, P., and Manley, S. (2013). Resolution Doubling in 3D-STORM Imaging through Improved Buffers. PLoS One *8*.

Palmer, D.J., Helms, J.B., Beckers, C.J.M., Orci, L., and Rothman, J.E. (1993). Binding of coatomer to Golgi membranes requires ADP-ribosylation factor. J. Biol. Chem. *268*, 12083–12089.

Parthasarathy, R. (2012). Rapid, accurate particle tracking by calculation of radial symmetry centers. Nat. Methods *9*, 724–726.

Tsin, Y., and Kanade, T. (2004). A correlation-based approach to robust point set registration. In Computer Vision-ECCV 2004, (Springer), pp. 558–569.

Wang, B., and Booth, M.J. (2009). Optimum deformable mirror modes for sensorless adaptive optics. Opt. Commun. *282*, 4467–4474.

Wang, Y., Schnitzbauer, J., Hu, Z., Li, X., Cheng, Y., Huang, Z.-L., and Huang, B. (2014). Localization events-based sample drift correction for localization microscopy with redundant cross-correlation algorithm. Opt. Express *22*, 15982–15991.
